# Gas-Phase Reactions of Cationic Vanadium-Phosphorus Oxide Clusters with C_2_H_*x*_ (*x=*4, 6): A DFT-Based Analysis of Reactivity Patterns

**DOI:** 10.1002/chem.201203050

**Published:** 2013-01-15

**Authors:** Nicolas Dietl, Xinhao Zhang, Christian van der Linde, Martin K Beyer, Maria Schlangen, Helmut Schwarz

**Affiliations:** [a] Institut für Chemie, Technische Universität BerlinStraße des 17. Juni 135, 10623 Berlin (Germany), Fax: (+49) 30-314-21102 E-mail: Maria.Schlangen@mail.chem.tu-berlin.deHelmut.Schwarz@mail.chem.tu-berlin.de; [b] Institut für Physikalische Chemie, Christian-Albrechts-Universität zu KielOlshausenstraße 40, 24098 Kiel (Germany); [c] Lab of Computational Chemistry and Drug Design, Laboratory of Chemical Genomics, Peking University Shenzhen Graduate SchoolShenzhen 518055 (P. R. China); [d] Chemistry Department, Faculty of Science, King Abdulaziz UniversityJeddah 21589 (Saudi Arabia) E-mail: HSchwarz@kau.edu.sa

**Keywords:** C–H activation, cluster compounds, density functional calculations, gas-phase reactions, reaction mechanisms

## Abstract

The reactivities of the adamantane-like heteronuclear vanadium-phosphorus oxygen cluster ions [V_*x*_P_4−*x*_O_10_]^.+^ (*x*=0, 2–4) towards hydrocarbons strongly depend on the V/P ratio of the clusters. Possible mechanisms for the gas-phase reactions of these heteronuclear cations with ethene and ethane have been elucidated by means of DFT-based calculations; homolytic C–H bond activation constitutes the initial step, and for all systems the P–O^.^ unit of the clusters serves as the reactive site. More complex oxidation processes, such as oxygen-atom transfer to, or oxidative dehydrogenation of the hydrocarbons require the presence of a vanadium atom to provide the electronic prerequisites which are necessary to bring about the 2e^−^ reduction of the cationic clusters.

In memoriam Detlef Schröder

## Introduction

**Scheme 1:**
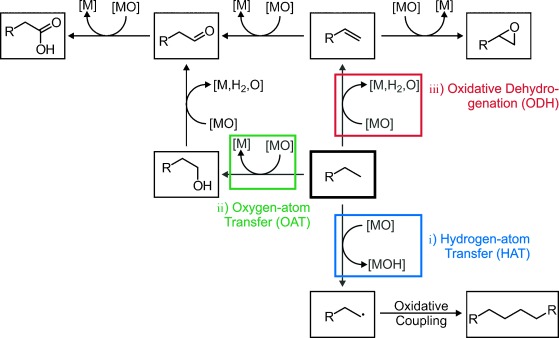
Schematic description of three basic oxidation processes of hydrocarbons by metal oxides [MO], all commencing with C–H bond activation.

Selective oxidation of hydrocarbons continues to constitute one of the major challenges in contemporary chemistry to solve global problems, such as an environmentally benign and economically feasible conversion of natural gas into value-added products.^1^ In this context, oxygen-based catalysts, especially metal oxides, play a particular role as powerful oxidation reagents.^2^ Despite the apparently large number of different pathways and mechanistic scenarios, formation of the various oxidation products of hydrocarbons by metal oxides can be classified in terms of three general reaction types (Scheme 1): i) hydrogen-atom transfer (HAT) from the hydrocarbon to the metal oxide to bring about oxidative coupling, ii) oxygen-atom transfer (OAT) to the organic substrate, and iii) oxidative dehydrogenation (ODH) of the latter. While there exist numerous effective homogeneous and heterogeneous catalysts which cover this rather broad spectrum of oxidation reactivity, it is no exaggeration to state the paucity of detailed knowledge about the intrinsic properties of many of the catalysts which, after all, control also the chemoselectivity of the three competing oxidation processes depicted in Scheme 1.^3^

To further improve the understanding on how such complex systems, for example, surfaces in heterogeneous catalysis may operate, often model systems have been studied as they can provide insights into what happens at the molecular level.^4^ One approach in the elucidation of mechanistic aspects employs reactivity studies which are conducted under near single-collision conditions in the gas phase. In fact, in the last two decades numerous state-of-the-art, mass-spectrometry based experiments with small metal-oxo clusters in combination with computational studies have revealed important details of the elementary steps for many chemical reactions.^5^ For example, hydrogen-atom abstraction from CH_4_ to generate CH_3_^.^ is viewed as the decisive step in the oxidative dehydrogenation and dimerization of methane,^6^ and many studies suggest the crucial role of oxygen-centered radicals to bring about homolytic C–H bond scission by (doped) metal oxides.2b, 7 The perhaps most compelling experimental evidence for this concept has been provided by the reactions of free aluminum-oxide cluster ions with methane:^8^ aluminum-oxide cluster cations with an odd number of aluminum atoms, thus lacking an oxygen-centered radical, do not react with methane at ambient conditions, in spite of a substantial thermodynamic driving force; in distinct contrast, oligomeric [(Al_2_O_3_)_*x*_]^.+^ (*x*=3, 4, 5) cluster ions possessing an even number of aluminum atoms and an oxygen-centered radical are remarkably reactive towards methane even at room temperature.

**Scheme 2:**
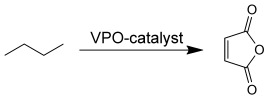
Industrial synthesis of maleic anhydride from *n*-butane.

In addition to the large number of homonuclear metal and non-metal oxides investigated,^8^, ^9^ more recently heteronuclear oxide clusters have been brought into focus, for example, [Al_*x*_V_*y*_O_*z*_]^+/−^ (*x*+*y*=2, 3, 4; *z*=3–10),^10^ [Ce_*x*_V_*y*_O_*z*_]^+^ (*x*+*y*=2, 3; *z*=4, 5, 6),^11^ or [(V_2_O_5_)_*x*_(SiO_2_)_*y*_]^+/−^ (*x*=1, 2; *y*=1–4),^12^ respectively. The considerable interest in studying these systems as model catalysts can be derived from their chemically close resemblance with practical heterogeneous catalysts. Bulk metal-oxide catalysts are often composed of multiple components, that is, mixed-metal oxo-frameworks, or employ metal oxides as non-innocent support materials.^13^ The transformation of *n*-butane to maleic anhydride by the so-called VPO-catalysts (Scheme 2), may serve as a well-known example for such a multi-component system in heterogeneous catalysis; this reaction involves the abstraction of eight hydrogen atoms from as well as the transfer of three oxygen atoms to C_4_H_10_—complexity at its best.^14^ Since the first commercial use of VPO catalysts for the synthesis of maleic anhydride in the early 80s, many experimental and theoretical studies have been carried out to investigate this remarkable transformation.^15^ However, the underlying reaction mechanisms are still only poorly understood and many conflicting hypotheses exist. For example, it was conjectured that structurally ill-defined phosphate species work as catalytically innocent, chemically inert linkers between the active vanadium-oxide sites in these mixed metal-oxide phosphates;14a however, this assumption was questioned by a gas-phase study on the open-shell oxide cation [P_4_O_10_]^.+^, in which the efficient activation even of methane, the most inert of all hydrocarbons, at ambient conditions by this metal-free oxide was demonstrated.9d Obviously, the central question about the actual nature of the active site in heteronuclear oxo-clusters and the particular role of the cluster components was once more raised.

Additional gas-phase studies supported the idea that also phosphorus sites may be active by demonstrating an increased reactivity of a polynuclear metal-free cluster towards various small hydrocarbons, for example, ethane, propane, and ethene.^16^ This phosphorus-oxide system is electronically and structurally closely related to the isostructural transition-metal oxide cluster [V_4_O_10_]^.+^, which had been thoroughly investigated earlier; it seems to be an appropriate model for surface-mediated C–H bond activation processes.^17^ Common to both cluster-oxide cations is the existence of a terminal oxygen-centered radical which proved to be crucial for the reactivity observed towards methane and higher hydrocarbons.9a,d, 16, 18 While [P_4_O_10_]^.+^ and [V_4_O_10_]^.+^ bring about thermal hydrogen-atom abstraction from methane, the reactivity patterns with ethane, propane and ethene differ quite remarkably. For example, [P_4_O_10_]^.+^ reacts with all substrates predominantly by homolytic C–H bond cleavage (HAT), while [V_4_O_10_]^.+^ gives rise to OAT to the higher hydrocarbons as well as to ODH.^19^ These differences in oxidation reactivities suggested a more systematic investigation at a molecular level, that is, the studies of mixed vanadium-phosphorus oxygen cluster ions [V_*x*_P_4−*x*_O_10_]^.+^ (*x*=1–3).^20^ While only the mixed cluster ions [V_2_P_2_O_10_]^.+^ and [V_3_PO_10_]^.+^ could be generated in the experiment, density functional calculations revealed for all three mixed clusters [VP_3_O_10_]^.+^, [V_2_P_2_O_10_]^.+^, and [V_3_PO_10_]^.+^ a phosphorus-bound oxygen-centered radical as the most stable isomer on the potential-energy surfaces (PESs). Thus, all C–H bond activation processes are initiated solely by the terminal phosphorus-bound oxygen atom. Not entirely unexpected with regard to the activation of methane by both oxides [P_4_O_10_]^.+^ and [V_4_O_10_]^.+^, the reactivities of [V_3_PO_10_]^.+^ and [V_2_P_2_O_10_]^.+^ towards methane are similar in that hydrogen-atom abstraction is observed with comparable efficiencies.9a,d, 20b However, the reactions of [V_3_PO_10_]^.+^ and [V_2_P_2_O_10_]^.+^ with C_2_ hydrocarbons give rise to new product distributions for the couples [V_3_PO_10_]^.+^/C_2_H_*x*_ and [V_2_P_2_O_10_]^.+^/C_2_H_*x*_ (*x*=4, 6). While homolytic C–H bond scission, according to Equations (1a) and (2a), and oxidative dehydrogenation, [Eqs. (1c) and (2c)], are observed, oxygen-atom transfer does not take place [Eqs. (1b) and (2b)].


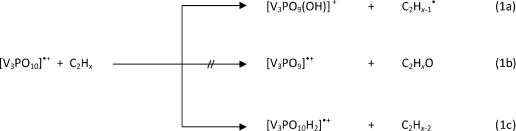



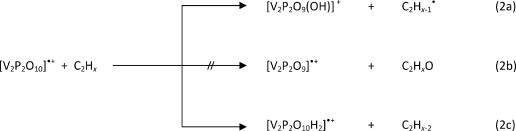


The questions raised by the experimental data (Table 1) can be summarized as follows: i) What are the reasonable reaction mechanisms for the various VPO-cluster ions in their thermal reactions with ethene and ethane? ii) Why does only the [V_4_O_10_]^.+^/C_2_H_4_-couple undergo exclusively oxygen-atom-transfer, but as soon as one phosphorus atom is present in the tetranuclear oxide cluster, oxygen-atom transfer to the hydrocarbon is no longer observed? iii) Why does [P_4_O_10_]^.+^ not permit the oxidative dehydrogenation of the C_2_ -hydrocarbons?

**Table 1:** Branching ratio of the product distributions in the reactions of [V_*x*_P_4−*x*_O_10_]^.+^ (*x*=0, 2–4) (OAT=oxygen-atom transfer, HAT=hydrogen-atom transfer, ODH=oxidative dehydrogenation).^16^, 18b, 20b

	C_2_H_4_			C_2_H_6_		
	OAT	HAT	ODH	OAT	HAT	ODH
[V_4_O_10_]^.+^	100	–	–	–	–	100^[a]^
[V_3_PO_10_]^.+^	–	36	64	–	79	21
[V_2_P_2_O_10_]^.+^	–	38	62	–	83	17
[P_4_O_10_]^.+^	–	100^[b]^	–	–	100	–

[a] According to our experimental results.^19^ [b] Minor contributions of single-electron transfer to produce [C_2_H_4_]^.+^ have not been taken into account.

Herein, we aim at providing a DFT-based explanation for the different reactivities exhibited by [V_*x*_P_4−*x*_O_10_]^.+^ (*x*=0, 2–4), or to put it bluntly “why composition counts!”.20b,c

## Results and Discussion

**Intrinsic properties of the [V**_***x***_**P_4−*x*_O_10_]**^.**+**^ **clusters (*x*=0, 2–4)**: Before discussing the relative energies of the intermediates and transition structures as well as the mechanistic details of the three reaction channels for the individual [V_*x*_P_4−*x*_O_10_]^.+^/C_2_H_4_ and [V_*x*_P_4−*x*_O_10_]^.+^/C_2_H_6_ systems (*x*=0, 2–4), some of the intrinsic properties of the individual clusters are addressed; this may be helpful in the discussion of the experimentally observed reactivity patterns.^21^ For example, to explain the preference for OAT observed in the reaction of [V_4_O_10_]^.+^ with ethene, and its complete absence for the phosphorus-containing systems, a closer look at the bond-dissociation energies (BDEs) for the terminal V–O_*t*_ and P–O_*t*_ bonds, respectively, is instructive (O_*t*_=terminal oxygen).^22^

**Figure 1:**
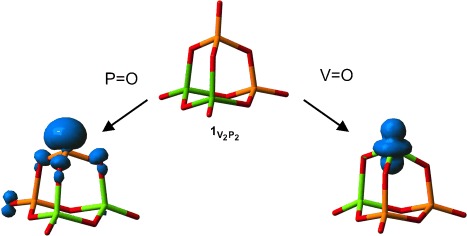
Two possible DFT-derived [V_2_P_2_O_9_]^.+^ isomers resulting from dissociation of a P–O^.^ or V–O^.^ bond of [V_2_P_2_O_10_]^.+^ (green V, yellow P, red O). The blue isosurfaces indicate the spin density within the respective cluster.

As shown in Table 2, BDE(V–O_*t*_) increases when phosphorus atoms are present in the cluster: In [V_4_O_10_]^.+^ (**1
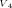
**), BDE(V–O_*t*_) amounts to only 280 kJ mol^−1^, which is 76 kJ mol^−1^ and 71 kJ mol^−1^ lower compared to the values obtained for the [V_3_PO_10_]^.+^ (**1
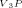
**) and [V_2_P_2_O_10_]^.+^ (**1
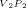
**) clusters, respectively.^23^ In contrast, the BDEs of the terminal P–O bonds for all phosphorus-containing clusters investigated are not affected by the vanadium/phosphorus ratio and lie around 400 kJ mol^−1^. These features can be explained by the fact that the most stable structures of all phosphorus-containing clusters possess a P–O_*t*_^.^ bond; a V–O_*t*_^.^ bond is only present in [V_4_O_10_]^.+^. Thus, energy in addition to BDE(V–O_*t*_)=280 kJ mol^−1^ as determined for [V_4_O_10_]^.+^ is necessary for [V_3_PO_10_]^.+^ and [V_2_P_2_O_10_]^.+^ to cleave the inert V=O bond.^24^ While for the heteronuclear clusters it is energetically favored to remove an electron from the P=O bond, generating a P–O^.^ instead of a V–O^.^ unit, the binding energy of the P–O^.^ versus the V–O^.^ bond is higher. An analysis of the nature of the singly occupied molecular orbitals (SOMOs) of the dissociation products [V_*x*_P_4−*x*_O_9_]^.+^ (*x*=2, 3) is quite helpful to understand the origin of these thermochemical differences; these SOMOs are utilized to form a single bond to oxygen in [V_*x*_P_4−*x*_O_10_]^.+^. As exemplified in Figure 1 for [V_2_P_2_O_10_]^.+^, loss of a terminal O atom from vanadium leads to a vanadium-centered radical having a d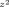 orbital as a SOMO. In contrast, cleavage of the P–O_*t*_^.^ bond in [V_2_P_2_O_10_]^.+^ gives rise to a phosphorus-centered SOMO possessing a s/p character (Figure 1); the latter is in comparison to the d orbital of the vanadium atom, more appropriate to form a single bond to the oxygen atom due to the better overlap with its sp^*x*^ orbital; similar properties of the SOMO were obtained for all P-containing cluster ions.

**Table 2:** BDEs (kJ mol^−1^) of the terminal V–O and P–O bonds in [V_*x*_P_4−*x*_O_10_]^.+^ (*x*=0, 2–4), calculated at the B3LYP/aug-cc-pVTZ//B3LYP/TZVP level of theory

	[V_4_O_10_]^.+^ (1 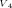 )	[V_3_PO_10_]^.+^ (1 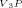 )	[V_2_P_2_O_10_]^.+^ (1 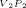 )	[P_4_O_10_]^.+^ (1 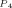 )
BDE (V–O_*t*_)	280	356	351	–
BDE (P–O_*t*_)	–	397	396	399

The thermochemistry of the competing HAT and ODH reaction channels depends on the relative energies of the [V_*x*_P_4−*x*_O_10_H]^+^ and [V_*x*_P_4−*x*_O_10_H_2_]^.+^ product ions, respectively. While the first hydrogen-atom abstraction results in the formation of closed-shell product ions concomitant with spin transfer to the emerging hydrocarbon radicals, respectively, open-shell systems are regenerated in the course of the second hydrogen-atom transfer under reduction of a vanadium or phosphorus atom, respectively, to the formal oxidation state +IV. As will be discussed later, the reduction to the oxidation state +IV is disfavored for phosphorus. Thus, [P_4_O_10_]^.+^ gives rise to the closed-shell HAT product [P_4_O_9_(OH)]^+^; formation of the open-shell product [P_4_O_10_H_2_]^.+^ via ODH is not possible due to thermochemical constraints. In contrast, the presence of redox-active vanadium opens up the latter reaction channel. The relative stabilities of the open*-* versus the closed-shell cations are indicated by the adiabatic ionization energies (*IE*s) of the respective neutral clusters (Table 3). The formation of open-shell cluster cations, that is, [V_*x*_P_4−*x*_O_10_]^.+^ (**1**), [V_*x*_P_4−*x*_O_9_]^.+^, and [V_*x*_P_4−*x*_O_10_H_2_]^.+^, is rather energy demanding in the case of the homonuclear phosphorus systems (*x*=0, Table 3); in contrast, the ionization energy required to produce the closed-shell cluster cation [P_4_O_10_H]^+^ is significantly lower (7.2 eV) than the *IE*s of the respective vanadium-containing systems (>8.5 eV).

**Table 3:** Adiabatic ionization energies (in eV) of V_*x*_P_4−*x*_O_10_, V_*x*_P_4−*x*_O_9_, V_*x*_P_4−*x*_O_10_H and V_*x*_P_4−*x*_O_10_H_2_ as calculated at the B3LYP/aug-cc-pVTZ//B3LYP/TZVP level of theory

	*x*=4	*x*=3	*x*=2	*x*=0
V_*x*_P_4−*x*_O_10_	11.2	10.7	11.1	12.2
V_*x*_P_4−*x*_O_9_	8.6^[a]^	9.7	10.2	11.4
V_*x*_P_4−*x*_O_10_H	8.7	8.5	8.8	7.2
V_*x*_P_4−*x*_O_10_H_2_	8.2^[a]^	8.0	7.9	9.7

[a] The ground state of the neutral cluster is a triplet, while the ground states of all other neutral clusters in the respective row are closed-shell singlets.

**Figure 2:**
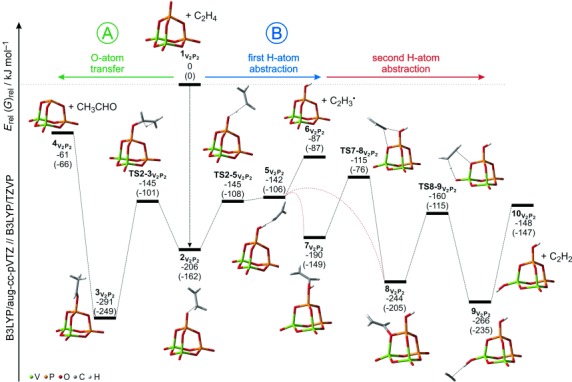
Potential-energy surface (PES) for the reactions of [V_2_P_2_O_10_]^.+^ with C_2_H_4_, calculated at the B3LYP/aug-cc-pVTZ//B3LYP/TZVP level of theory (green V, yellow P, red O, gray C, white H). The electronic energies and relative Gibbs free energies (in parenthesis) are given in kJ mol^−1^ and corrected for unscaled zero-point energy contributions.

**Ion/molecule reactions with C_2_H_4_**: Having discussed some of the thermochemical properties of the educt and product ions, next we will outline the mechanistic details for the reactions of the [V_2_P_2_O_10_]^.+^/ethene system; this may serve as a representative example for all the VPO-cluster ions investigated (the Cartesian coordinates of all structures can be found in the Supporting Information, electronic energies and the relative free energies are summarized in Table 4), and the corresponding potential-energy profile is shown in Figure 2.

**Table 4:** Relative energies and free energies (in parenthesis), given in kJ mol^−1^, of the transition states and intermediates for oxygen-atom transfer (OAT), hydrogen-atom transfer (HAT), and oxidative dehydrogenation (ODH) for the reactions of [V_*x*_P_4−*x*_O_10_]^.^ (*x*=0, 2–4) with C_2_H_4_, respectively, calculated at the B3LYP/aug-cc-pVTZ//B3LYP/TZVP level of theory

	[V_4_O_10_]^.+^	[V_3_PO_10_]^.+^	[V_2_P_2_O_10_]^.+^	[P_4_O_10_]^.+^
**2**	−203 (−167)	−192 (−152)	−206 (−165)	−244 (−207)
**TS2**-**3** (OAT-TS)	−190 (−148)	−134 (−92)	−145 (−101)	−102 (−70)
**3**	−342 (−330)	−279 (−237)	−291 (−249)	−191 (−189)
**4** (OAT-product)	−176 (−179)	−59 (−64)	−61 (−66)	−57 (−60)
**2**	−203 (−167)	−192 (−152)	−206 (−165)	−244 (−207)
**TS2**-**5** (HAT-TS)	−125 (−90)	−127 (−92)	−145 (−108)	–
**5**	−123 (−90)	−129 (−94)	−142 (−106)	−224 (−193)
**6** (HAT-product)	−68 (−71)	−82 (−83)	−87 (−87)	−100 (−101)
**7**	−292 (−249)	−180 (−140)	−190 (−149)	−92 (−58)
**TS7**-**8**	−119 (−72)	−96 (−57)	−115 (−76)	–
**8**	−310 (−268)	−236 (−190)	−244 (−205)	–
**TS8**-**9** (ODH-TS)	−159 (−109)	−148 (−105)	−160 (−115)	−33^[a]^ (5)
**9**	−268 (−236)	−262 (−232)	−266 (−235)	−146 (−119)
**10** (ODH-product)	−239 (−234)	−239 (−236)	−148 (−147)	−105 (−103)

[a] Directly linking intermediates **7
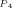
** and **9
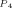
**.

In the first step, a carbon atom of ethene coordinates in a radicophilic fashion to the terminal phosphorus-bound oxygen atom of the cluster to form intermediate **2** in which the spin density is mainly located at the terminal carbon atom for all of the vanadium-containing intermediates **2
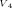
**, **2
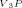
**, and **2
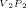
** (0.866, 0.893, and 0.866, respectively); in contrast, the spin is distributed over the two carbon atoms in **2
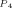
** (0.310 at the proximal and 0.608 at the terminal carbon atom). A comparison of the C–O, C–C, and P–O bond lengths in **2** for the individual clusters also reveals a notable effect due to the presence of vanadium. For all systems investigated, *r*(C–O) in **2** is longer compared to a C–O bond in for example, ethanol (1.431 Å); however, while a smooth increase of *r*(C–O) is observed in going from **2
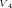
** via **2
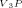
** to **2
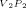
** (1.517 Å, 1.583 Å, and 1.619 Å, respectively), *r*(C–O) in the pure phosphorus intermediate **2
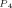
** is with 2.021 Å much more elongated. Though less pronounced, decreasing trends in the same direction are observed for the C–C and P–O bond lengths of the four systems. The addition of ethene to the cluster shortens the P–O bond from 1.599 Å in **1
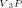
** to 1.539 Å in **2
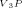
**, and from 1.593 Å in **1
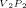
** to 1.530 Å in **2
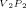
**, respectively; the C–C bonds of **2** are lengthened from 1.326 Å in free C_2_H_4_ to 1.461 Å in **2
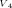
**, 1.455 Å in **2
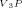
**, and 1.449 Å in **2
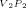
**, respectively. In contrast, the shortening of the P–O bond is the highest for the [P_4_O_10_]^⋅+^/C_2_H_4_ cluster (from 1.569 Å in **1**_P4_ to 1.493 Å in **2
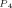
**) while the elongation of the C–C bond is the smallest for this particular system (from 1.326 Å in free ethene to 1.408 Å, respectively); thus, the P–O and the C–C bonds are the shortest for the vanadium-free intermediate **2
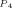
**.

**Scheme 3:**
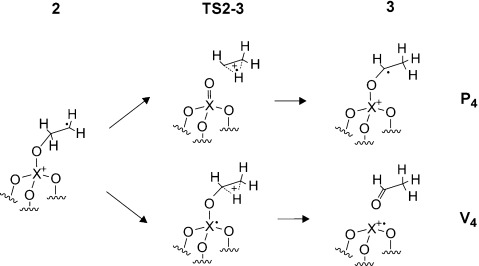
Schematic description of the process **2→TS2**-**3→3** for the homonuclear [X_4_O_10_]^.+^/C_2_H_4_ couples (X=P, V).

Starting from intermediate **2**, two different reactions channels A (OAT) and B (HAT and ODH) are accessible (Figure 2). In pathway A, which has already been identified by Castleman and co-workers for the reaction of [V_4_O_10_]^.+^ with C_2_H_4_,18b a hydrogen atom from the oxygen-bound methylene unit undergoes a 1,2 migration to the terminal carbon atom via transition structure **TS2**-**3** to form intermediate **3**. The binding situation of the newly generated acetaldehyde building block to the cationic cluster fragment in **3** differs significantly for the pure phosphorus cluster as compared with the vanadium-containing systems: While the C–O bond lengths in **3
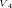
**, **3
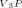
**, and **3
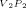
** (1.242 Å, 1.262 Å, and 1.266 Å, respectively) are similar to that of *r*(C–O) in free acetaldehyde (1.205 Å), *r*(C–O) in **3
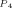
** is with 1.472 Å relatively long; these structural characteristics are inversely mirrored in the shortening of the P–O bond lengths to 1.745 Å, 1.733 Å, and 1.526 Å in **3
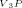
**, **3
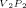
**, and **3
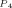
**, respectively. Remarkable differences among the clusters investigated also concern the spin distributions of the intermediates and transition structures for the intramolecular hydrogen migration, that is, **2**→**TS2**-**3**→**3**. In line with avoiding a high spin density at a P atom and the energetic disadvantage of an associated reduction to the formal oxidation state P^+V^→P^+IV^ as described above, the spin in **3
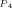
** is located at the C2 unit, that is, at the proximal carbon atom of the ligand (Scheme 3); here, the transfer of the unpaired electron takes place only during the reductive elimination of acetaldehyde, **3
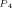
**→**4
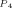
**. The bonding situation is different for the vanadium-containing couples [V_*x*_P_4−*x*_O_10_]^•+^/C_2_H_4_ (*x*=2–4): In **TS2-3** the spin density has already been shifted to a vanadium atom of the cluster; thus, the actual hydrogen transfer **2**→**TS2-3**→**3** corresponds rather to a 1,2 hydride shift within a cationic, closed-shell C2 unit (the C2 units in transition structures **TS2**-**3** possess a positive charge for all systems investigated), as exemplified for **TS2**-**3
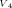
** in Scheme 3; this process is energetically favored as compared to the energy-demanding 1,2 H-atom migration within the radical-cation C2 unit in **TS2**-**3
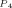
**.

The alternative to the OAT pathway shown in Figure 2, that is, the transfer of an oxygen atom from a vanadium-bound oxygen of [V_2_P_2_O_10_]^.+^ is thermodynamically favored; the same holds true for [V_3_PO_10_]^.+^ (see Table 2). Formation of the resulting products **4
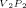
**–**VO** and **4
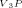
**–**VO** are more exothermic (exergonic) by −45 (−43) kJ mol^−1^ and −41 (−41) kJ mol^−1^, respectively, than the corresponding product ions of the OAT mechanism depicted in Figure 2. However, a rather energy demanding intramolecular spin-density transfer from the P=O to the V=O site is required,20a and the associated adduct complexes **2
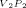
**–**VO** and **2
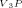
**–**VO**, in which ethene is coordinated to the VO side of the cluster, are 59 (56) kJ mol^−1^ (**2
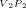
**–**VO**) and 59 (54) kJ mol^−1^ (**2
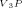
**–**VO**) less stable than the P=O-bound isomers **2
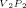
** and **2
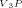
**, respectively. The transition structures **TS2**-**3
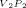
**–**VO** and **TS2**-**3
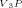
**–**VO** are with −141 (−101) kJ mol^−1^ and −120 (−81) kJ mol^−1^ (relative to the reactants, respectively) comparable in energy to the barriers of the OAT pathway A depicted in Figure 2. Thus, both OAT variants are accessible at thermal conditions for the mixed clusters; note, however, that a kinetic barrier caused by the intramolecular spin-density transfer might be involved in the initial coordination of C_2_H_4_ to a non-radical V=O site of the cluster, as reported for the reaction of [V_3_PO_10_]^.+^ with methane.20a Regarding the electronic structures of transition states **TS2**-**3
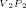
**–**VO** and **TS2**-**3
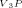
**–**VO**, the depicted situation for **TS2**-**3
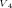
** in Scheme 3 holds true also for these systems; the process corresponds rather to a 1,2 hydride shift within the cationic hydrocarbon fragment as the spin is mainly localized at the proximal vanadium atom.

Pathway B results either in HAT or ODH and commences with a hydrogen-atom transfer from the oxygen-bound methylene unit in **2** to this very oxygen atom (**TS2**-**5**) thus generating a oxo-hydroxo cluster with an ethenyl radical ligand; the latter is loosely bound to the cluster via the hydrogen atom of the newly generated OH group. In the energy profile, Figure 2, the energy of **TS2**-**5** is slightly lower than the energy of the resulting intermediate **5**; this is due to zero-point energy and free-energy corrections. The fact that transition state **TS2**-**5** and intermediate **5** are very close in energy is reasonable, since the only structural reorganization in this step corresponds to the widening of the binding angle of the C_2_H_3_ fragment to the cluster. This HAT-reaction path has been located for all vanadium-containing clusters, while **TS2**-**5
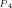
** could not be located for the [P_4_O_10_]^.+^/C_2_H_4_-couple on the potential-energy surface (PES). Furthermore, the nature of intermediate **5
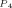
** differs remarkably from the structures of **5
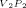
**, **5
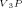
**, and **5
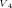
**. In the latter intermediates, the C–H bond is already strongly elongated (*r*(C–H) 1.675 Å for **5
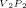
**, 1.756 Å for **5
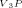
**, and 1.643 Å for **5
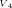
**) and the newly formed O–H bond is rather short (*r*(O–H) 1.053 Å for **5
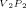
**, 1.030 Å for **5
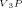
**, and 1.051 Å for **5
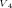
**). In **5
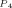
**, however, the C–H bond is only slightly elongated (*r*(C–H)=1.102 Å) and the O–H distance amounts to 1.858 Å. Thus, **5
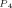
** rather corresponds to a hydrogen-bridged^25^ adduct complex [P_4_O_10_⋅⋅⋅H⋅⋅⋅C_2_H_3_]^.+^ directly generated from the educts instead of being formed via **2
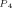
**→**TS2**-**5
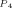
**→**5
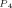
**; similar adduct complexes could not be located for the vanadium-containing clusters with ethene. According to a relaxed scan of the C–H bond, the ethenyl radical C_2_H_3_^.^ is liberated from **5
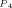
** without the involvement of any further intermediates or transition structures. In addition to the structural differences of **5
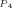
** as compared with **5
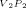
**, **5
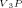
**, and **5
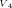
**, also the formation of **5
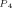
** is with −224 kJ mol^−1^ significantly more exothermic than that of the other HAT-intermediates (−142 kJ mol^−1^ for **5
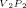
**, **−**129 kJ mol^−1^ for **5
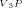
**, and −123 kJ mol^−1^ for **5
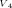
**). In line with these findings, the energy necessary for the liberation of the C_2_H_3_^.^ radical from **5** to form the hydrogen-atom transfer (HAT) product **6** amounts to 124 kJ mol^−1^ for [P_4_O_9_(OH)]^+^ (**6
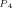
**), compared to only 55 kJ mol^−1^ for both [V_2_P_2_O_9_(OH)]^+^ (**6
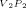
**) and [V_4_O_9_(OH)]^+^ (**6
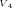
**), and 47 kJ mol^−1^ for [V_3_PO_9_(OH)]^+^ (**6
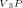
**), respectively.

**Figure 3:**
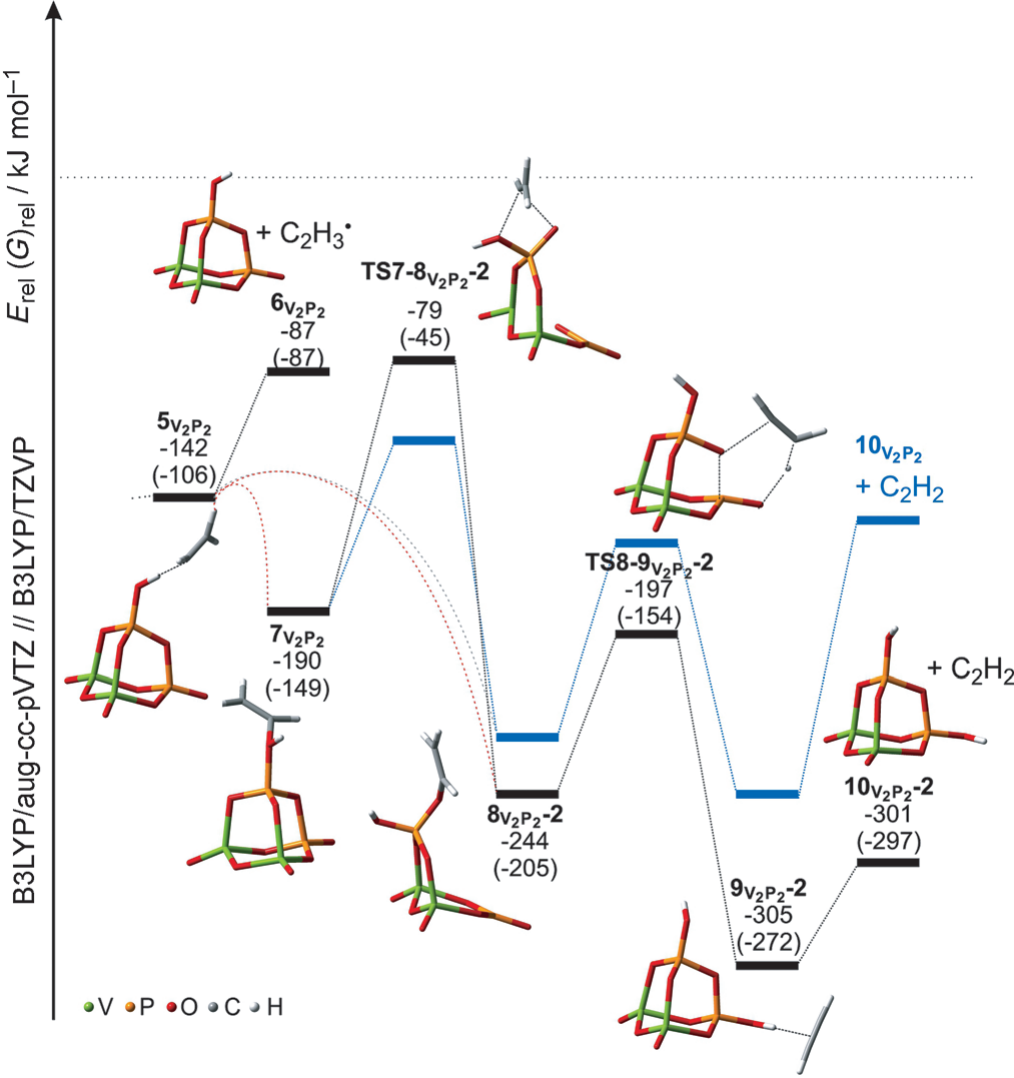
Alternative mechanism for the ODH-reaction of [V_2_P_2_O_10_]^.+^ with C_2_H_4_, calculated at the B3LYP/aug-cc-pVTZ//B3LYP/TZVP level of theory (green V, yellow P, red O, gray C, white H). The electronic energies and relative Gibbs free energies (in parenthesis) are given in kJ mol^−1^ and corrected for unscaled zero-point energy contributions. The blue line shows the pathway from Figure 2.

Structure **5
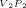
** in Figure 2 constitutes to a common intermediate for both the HAT and the ODH pathways. In competition with the formation of the HAT product **6**, the incipient C_2_H_3_^.^ radical can rebind to the oxygen atom of the newly formed hydroxyl group thus producing structure **7
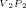
**; due to the rather shallow nature of the PES in this region, a transition state **TS5**-**7** could not be located on the PES. However, the unsuccessful attempts to locate this transition state suggest that the barrier for this rebound step is lower than the liberation of the hydrocarbon fragment resulting in the HAT product **6**. Regarding the structure of clusters **7**, all vanadium-containing cations **7
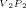
**, **7
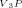
**, and **7
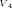
** possess the bonding pattern depicted in Figure 2 with the spin density exclusively located at one of the vanadium atoms within the cluster skeleton. For the pure phosphorus system instead, the rebound step is associated with an opening of the cluster cage thus leading to a distorted structure of **7
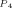
** (see the Supporting Information). For the vanadium-containing clusters the reaction continuous via transfer of the C_2_H_3_ fragment to a bridging, vanadium-bound oxygen atom. In the case of [V_2_P_2_O_10_]^.+^, the C_2_H_3_ fragment can alternatively be transferred to the bridging, phosphorus-bound oxygen atom; this pathway is described further below. Note also that starting from **5** the direct migration of the C_2_H_3_^.^ radical to a bridging oxygen atom also results in the formation of **8** and represents an alternative mechanistic scenario; however, locating the corresponding transition structure was not successful. From **8**, transfer of a hydrogen atom from the terminal CH_2_ unit to the adjacent V=O site, thus generating a V–OH group, completes the ODH process. The barrier height of the step **8**→**TS8**-**9**→**9** depends strongly on the system, ranging from 84 and 88 kJ mol^−1^ for [V_2_P_2_O_10_]^.+^ and [V_3_PO_10_]^.+^, respectively, to 151 kJ mol^−1^ for [V_4_O_10_]^.+^. For the latter, the high intrinsic barrier is due to the relative low energy of intermediate **8
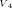
**, in which the bond length of bridging μ-OCHCH_2_ ligand to the V(OH) and VO_*t*_ units, respectively, are quite similar (*r*((HO)V–OCHCH_2_)=2.003 Å, *r*(O_*t*_V–OCHCH_2_)=1.918 Å); in contrast, the μ-OCHCH_2_ ligand in **8
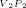
** and **8
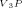
** is more strongly bound to the (HO)P unit at the expense of the CH_2_CHO–VO_*t*_ bond, respectively (**8
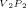
**: *r*((HO)P–OCHCH_2_)=1.691 Å, *r*(O_*t*_V–OCHCH_2_)=2.069 Å; **8
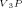
**: *r*((HO)P–OCHCH_2_)=1.708 Å, *r*(O_*t*_V–OCHCH_2_)=2.071 Å). The relative energies of the respective transition structures **TS8**-**9** are however comparable (within a range of 12 kJ mol^−1^) when related to the entrance channel, respectively. As to the homonuclear phosphorus cluster [P_4_O_10_]^.+^, intermediates **7** and **9** are linked via a single transition state; here, the second hydrogen atom is transferred directly to a terminal P=O unit (see the Supporting Information); however, this process is 67 kJ mol^−1^ higher in energy compared to the generation of the HAT product. For all vanadium-containing clusters, the spin distribution remains by and large unchanged along the reaction sequence **7**→**TS7**-**8**→**8**→**TS8**-**9**→**9**, carrying most of the spin density by a vanadium atom which is remote from the reactive sites. From **9**, the weakly bound acetylene ligand can easily be liberated to yield the experimentally observed oxidative-dehydrogenation (ODH) product [V_2_P_2_O_10_H_2_]^.+^ (**10**).^26^ As already mentioned above, an alternative reaction pathway has been identified for the ODH-process of the [V_2_P_2_O_10_]^.+^ cluster in which a structural isomer of **10
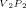
**, that is, **10
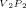
**–**2** in Figure 3, is generated bearing two P–OH moieties instead of the P–OH/V–OH groups as in **10
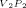
**. The relative energy (free energy) of **10
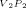
**–**2** is 153 (150) kJ mol^−1^ lower compared to the one of **10
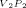
**; however, the corresponding reaction mechanism generating the former product ion (Figure 3) is kinetically more energy demanding than the HAT exit channel as well as the ODH pathway shown in Figure 2, that is, the hydrocarbon migration from the terminal- to the bridging oxygen atom (**TS7**-**8
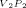
–2**) is 8 (42) kJ mol^−1^ and 36 (31) kJ mol^−1^ higher in (free) energy compared to the HAT product **6
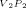
** and to **TS7**-**8
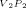
**–**2**, respectively. Alternatively, starting from **5
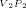
** with a direct rebound of the C_2_ fragment to the bridging oxygen atom of the P-O-P unit via **5
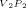
**→**8
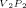
**–**2** might be possible but also here the PES is rather flat and the respective transition structure could not be located. Intermediate **8
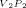
**–**2** corresponds to an open-cage cluster with a planar PO_3_ unit generated by the cleavage of the internal P-O-P bond. Next, the transfer of a second hydrogen atom completes the ODH reaction, and the process is accompanied with the regeneration of the closed-cage structure **9
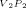
**–**2**; the intrinsic barrier **8
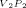
**–**2**→**TS8**-**9
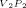
**–**2** is about 40 kJ mol^−1^ lower than the related barrier **8
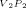
**→**TS8**-**9
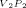
** shown in Figure 2.

As to the well-studied homonuclear clusters [V_4_O_10_]^.+^ and [P_4_O_10_]^.+^, the reactivity patterns observed in the reaction with ethene are consistent with the relative energies of the competing pathways calculated in this study. For [V_4_O_10_]^.+^, OAT is kinetically and thermochemically favored; the same holds true for the HAT pathway in the case of [P_4_O_10_]^.+^ for which no barrier exists along the reaction path. The latter process gains in importance in going from [V_4_O_10_]^.+^ via [V_3_PO_10_]^.+^ and [V_2_P_2_O_10_]^.+^ to [P_4_O_10_]^.+^ indicating that i) the hydrogen-atom affinity of the P–O_*t*_^.^ unit in [P_4_O_10_]^.+^ is higher compared to that of the V–O_*t*_^.^ entity in [V_4_O_10_]^.+^ and that ii) the newly generated PO_*t*_–H bond increases in strength with the number of phosphorus atoms being present in the cluster. In contrast, the reaction energies for an oxygen-atom transfer from [V_3_PO_10_]^.+^, [V_2_P_2_O_10_]^.+^, and [P_4_O_10_]^.+^ to C_2_H_4_ are similar and OAT is most exothermic for [V_4_O_10_]^.+^ (Table 4). Note that the HAT and OAT reaction channels are different with respect to the redox chemistry: While in the OAT process [V_*x*_P_4−*x*_O_10_]^.+^+C_2_H_4_→[V_*x*_P_4−*x*_O_9_]^.+^+C_2_H_4_O a reduction from +V to +IV takes place for V (*x*=4) and P (*x*=0, 2, 3), respectively, the formal oxidation states of the V and P atoms do not change in the course of HAT.

The branching ratio of OAT versus HAT/ODH is mainly determined by the relative energies of the barriers **TS2**-**3** versus **TS2**-**5** in Figure 2, respectively; both are lower in energy compared to the entrance channel for all clusters investigated, Table 4. As mentioned above, the number of P atoms in the [V_*x*_P_4−*x*_O_10_]^.+^ (*x*=0, 2, 3) clusters has a negligible effect on the strength of the corresponding BDEs- (P–O_*t*_) and thus affects the energies of the OAT products [V_*x*_P_4−*x*_O_9_]^.+^ (*x*=0, 2, 3) only marginally. However, the relative energies of the corresponding OAT transition structures **TS2**-**3** increase discontinuously with the number of P atoms (Table 4). With respect to the HAT channel, the replacement of one vanadium atom by phosphor, that is, [V_4_O_10_]^.+^ versus [V_3_PO_10_]^.+^, has only a minor effect on the relative energies of the corresponding **TS2**-**5** while the increasing thermochemical preference for HAT in going from [V_2_P_2_O_10_]^.+^ to [P_4_O_10_]^.+^ is even more pronounced when looking at the respective transition structures. As a result, the difference between the relative energies (free energies) of **TS2**-**3** and **TS2**-**5** amount to −65 (−58) kJ mol^−1^, −7 (0) kJ mol^−1^, and 0 (7) kJ mol^−1^, for the [V_4_O_10_]^.+^/C_2_H_4_, [V_3_PO_10_]^.+^/C_2_H_4_, and [V_2_P_2_O_10_]^.+^/C_2_H_4_ systems, respectively; for the [P_4_O_10_]^.+^/C_2_H_4_-system there is no barrier for the HAT process. Thus, the calculations are consistent with the experimentally observed selectivities for OAT and HAT in the gas-phase reactions of [V_4_O_10_]^.+^ and [P_4_O_10_]^.+^ with C_2_H_4_, respectively. Although OAT in the reaction of [V_3_PO_10_]^.+^ and [V_2_P_2_O_10_]^.+^ with C_2_H_4_ is exergonic and kinetically allowed at room temperature, the thermodynamic preference of the HAT and ODH products may account for the absence of OAT in the experiments. The non-observation of the second hydrogen transfer leading to ODH in the case of the homonuclear system [P_4_O_10_]^.+^/C_2_H_4_, however, is in line with the calculations since the corresponding transition structure **TS8**–**9
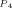
** is much higher in energy compared to the HAT exit channel so that ODH cannot compete and is thus not observable under the experimental conditions.

**Figure 4:**
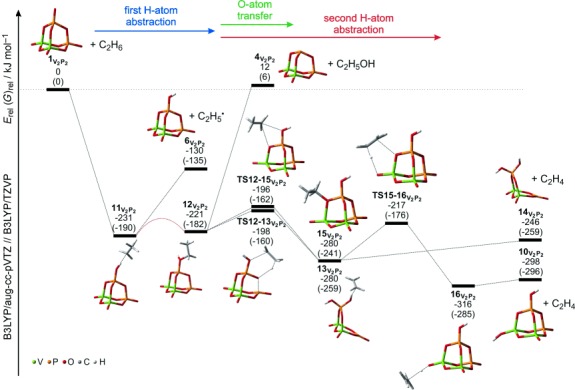
Potential-energy surface for the reactions of [V_2_P_2_O_10_]^.+^ with C_2_H_6_, calculated at the B3LYP/aug-cc-pVTZ//B3LYP/TZVP level of theory (green V, yellow P, red O, gray C, white H). The electronic energies and relative Gibbs free energies (in parenthesis) are given in kJ mol^−1^ and corrected for unscaled zero-point energy contributions.

**Ion/molecule reactions with C_2_H_6_**: [V_2_P_2_O_10_]^.+^ has also been chosen as cluster prototype to demonstrate the reactions of the various cluster ions [V_*x*_P_4−*x*_O_10_]^.+^ (*x*=0, 2–4) with ethane, and the potential-energy profile is shown in Figure 4 (Cartesian coordinates of all structures can be found in the Supporting Information and the relative electronic and free energies are summarized in Table 5).

**Table 5:** Relative energies and free energies (parenthesis), given in kJ mol^−1^, for the OAT, the HAT and the ODH channels calculated for the reactions of [V_*x*_P_4−*x*_O_10_]^.+^ (*x*=0, 2–4) with C_2_H_6_, at the B3LYP/aug-cc-pVTZ//B3LYP/TZVP level of theory

	[V_4_O_10_]^.+^	[V_3_PO_10_]^.+^	[V_2_P_2_O_10_]^.+^	[P_4_O_10_]^.+^
**11**	–	−171 (−141)	−231 (−190)	−213 (−185)
**6** (HAT-product)	−112 (−118)	−124 (−130)	−130 (−135)	−143 (−148)
**12**	−272 (−235)	−209 (−171)	−221 (−182)	−111 (−78)
**4** (OAT-product)	−103 (−108)	14 (7)	12 (6)	16 (11)
**12**	−272 (−235)	−209 (−171)	−221 (−182)	−111 (−78)
**TS12**-**13** (ODH-TS)	−158 (−115)	−169 (−129)	−198 (−160)	23 (50)
**13**	−163 (−141)	−180 (−159)	−280 (−259)	−81 (−62)
**14** (ODH-product)	−133 (−144)	−149 (−105)	−246 (−259)	−35 (−51)
**12**	−272 (−235)	−209 (−171)	−221 (−182)	−111 (−78)
**TS12**-**15**	−186 (−146)	−168 (−132)	−196 (−162)	–
**15**	−312 (−270)	−251 (−214)	−280 (−241)	–
**TS15**-**16** (ODH-TS)	−211 (−167)	−204 (−162)	−217 (−176)	−87^[a]^ (−53)
**16**	−318 (−286)	−312 (−282)	−316 (−285)	−195 (−170)
**10** (ODH-product)	−287 (−289)	−287 (−291)	−298 (−296)	−153 (−158)

[a] Directly linking intermediates **12
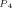
** and **16
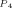
**.

Similar to the reactions of these cluster-cation radicals with methane, coordination of C_2_H_6_ to the clusters [V_*x*_P_4−*x*_O_10_]^.+^ (*x*=0, 2, 3) also results in a barrierless generation of intermediate **11
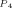
**, **11
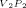
**, and **11
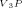
**, respectively; once more, the P–O_*t*_^.^ unit constitutes the reactive site for the hydrogen-atom transfer; in contrast to the reactions with ethene, no encounter complex has been located on the potential-energy surface. With respect to the homonuclear [V_4_O_10_]^.+^ cluster, the corresponding intermediate **11
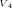
** could not be located as a local minimum on the PES; instead, the hydrocarbon fragment migrates in the course of the HAT reaction around the terminal oxygen atom and rebinds to it thus forming directly intermediate **12
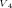
** bearing an intact ethanol ligand. Quite recently, the formation of CH_3_OH has also been described as a combination of HAT and CH_3_^.^ rebound in the reaction of [Al_2_O_3_]^.+^ with CH_4_ under thermal conditions; in addition, this system also gives rise to the direct conversion of methane to formaldehyde.^27^

For the phosphorus-containing clusters, **11
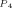
**, **11
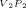
**, and **11
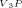
** serve as common intermediates to branch out in three different reaction paths: first, the weakly-bound ethyl group can be eliminated yielding the cationic HAT product **6**. As an alternative, rebound of the ethyl group to the newly generated OH group may occur to form C_2_H_5_OH which upon liberation of the alcohol gives rise to the OAT product **4**. Here too, the transition state could not be located, the smooth PES in this region and the non-existing barrier for the **11
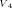
**→**12
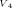
** process indicate however a low activation energy for the rebound step. The C–O bond lengths in **12
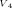
**, **12
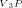
**, and **12
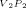
** increase with the number of phosphorus atoms (1.517 Å, 1.567 Å, and 1.588 Å, respectively) and all three are elongated compared to free ethanol (1.430 Å); the C–O bond length in **12
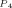
** amounts to 1.559 Å. While **12
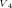
**, **12
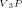
**, and **12
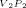
** correspond to closed-cage structures, a distorted open-cage structure was found for **12
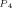
** as the local minimum with one (C_2_H_5_OH)PO–P being broken, thus forming a bend PO_3_ moiety with a threefold coordinated phosphorus atom at which 50 % of the spin is located.

The third reaction path starting from phosphorus-containing intermediates **12** comprises two variants of ODH reactions. In the first scenario, the open-cage structure **13** can be formed from intermediate **12** via **TS12**-**13**; this transition state corresponds to the second HAT from the terminal methyl group to the bridging P–O_*b*_ unit thus leading to a (O_*b*_)_2_P(OH)_2_ moiety to which the newly generated ethene molecule is only weakly coordinated. Regarding the electronic structures of the vanadium-containing clusters, the spin density is located at one of the vanadium atoms throughout the whole reaction sequence. In contrast, the spin in **TS12**-**13
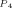
** is transferred from the bent PO_3_ unit to an adjacent phosphorus atom which is accompanied with a second P–O bond scission (*r*(P–O)=1.607 Å and 2.943 Å in **12
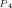
** and **TS12**-**13
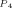
**, respectively), thus resulting in a planar PO_3_ unit possessing two terminal oxygen atoms. The latter process requires 138 kJ mol^−1^ activation energy and is thus 23 kJ mol^−1^ higher in energy compared to the entrance channel. In all transition structures, one hydrogen atom of the terminal CH_3_ group is transferred to the oxygen-atom and the respective C–H bond is elongated (*r*(C–H) 1.125 Å for **TS12**-**13
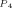
**, 1.118 Å for **TS12**-**13
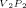
**, 1.130 Å for **TS12**-**13
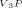
**, and 1.191 Å for **TS12**-**13
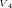
**, respectively). Liberation of C_2_H_4_ yields the product ion **14**.

In the second ODH-mechanism, the reaction sequence **12**→**TS12**-**15**→**15**→**TS15**-**16**→**16**→**10** parallels the pathway for the oxidative dehydrogenation of ethene as discussed above (Figure 2). Briefly, for all vanadium-containing clusters the hydrocarbon fragment is first transferred to a bridging oxygen atom via the isomerization sequence **12**→**TS12**-**15**→**15**; the barrier of this process is almost isoenergetic to the alternative transition state **TS12**-**13** for [V_2_P_2_O_10_]^.+^ (+2 kJ mol^−1^) and [V_3_PO_10_]^.+^ (+1 kJ mol^−1^), respectively, while it is distinctly lower for [V_4_O_10_]^.+^ (−28 kJ mol^−1^). Subsequently, the second HAT process occurs from intermediate **15** via **TS15**-**16** in which the hydrogen atom is transferred to the adjacent V–O_*t*_ unit. Again, the homonuclear phosphorus cluster represents an exception in that the second C–H bond activation occurs directly from **12
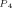
** at one of the terminal P–O_*t*_ units, without a prior migration of the hydrocarbon to a bridging oxygen atom. From **16**, the ethene molecule is easily liberated, leading to the formation of the thermochemically preferred closed-cage product **10** (Table 5).

**Figure 5:**
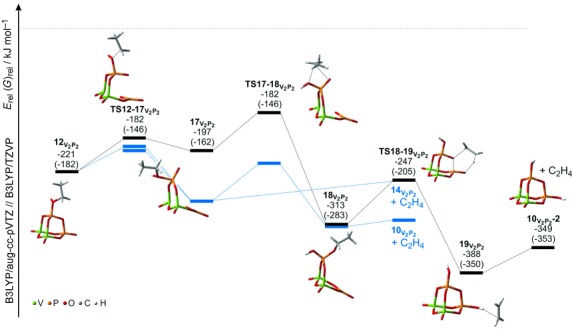
Alternative mechanism for the ODH-reaction of [V_2_P_2_O_10_]^.+^ with C_2_H_6_, calculated at the B3LYP/aug-cc-pVTZ//B3LYP/TZVP level of theory (green V, yellow P, red O, gray C, white H). The electronic energies and relative Gibbs free energies (in parenthesis) are given in kJ mol^−1^ and corrected for unscaled zero-point energy contributions. The blue lines show the initial pathways from Figure 4.

Similar to the [V_2_P_2_O_10_]^.+^/C_2_H_4_ couple, an alternative pathway for the oxidative dehydrogenation in the reaction of [V_2_P_2_O_10_]^.+^ with C_2_H_6_ has been found in which the more stable product ion **10
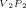
**–**2** (Figure 5) is generated bearing two P–OH units instead of one V–OH and one P–OH group. However, this process is kinetically more demanding compared to the two ODH pathways described above. Starting from intermediate **12
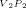
**, the cage structure is opened via **TS12**-**17
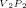
**, before the transfer of the ethyl radical to the newly formed P–O_*t*_ unit via **TS17**-**18
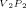
** takes place; the latter corresponds to the transition structure with the highest relative energy (−182 kJ mol^−1^). The second C–H bond activation of the oxidative dehydrogenation concomitant with the regeneration of the closed-cage structure **19
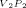
** occurs in the sequence **18
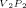
**→**TS18**-**19
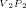
**→**19
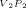
**. Finally, elimination of the weakly-bound ethene brings about formation of **10
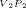
**–**2**; the overall process is associated with a significant thermochemical driving force of −349 kJ mol^−1^.

While the reaction channels discussed represent the energetically favored pathways for ODH, many other isomeric intermediates are possible as well, depending on the coordination of the hydrocarbon, the hydrogen atom, etc. However, the rate-determining activation barriers of the two ODH pathways determined for the [V_3_PO_10_]^.+^/C_2_H_6_ couple are **TS12**-**13
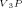
** and **TS12**-**15
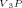
**. While these transition structures with relative energies of −169 and −168 kJ mol^−1^, respectively, are almost isoenergetic, the associated product ions are quite different energetically with the formation of **10
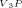
** being significantly more exothermic, that is, 138 kJ mol^−1^, compared to **14
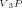
**. For the [V_4_O_10_]^.+^/C_2_H_6_ system, **10
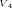
** is not only thermochemically favored by approximately 154 kJ mol^−1^, but also the respective transition state **TS12**-**15
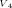
** is 28 kJ mol^−1^ lower in energy than the alternative transition state **TS12**-**13
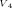
** leading to **14
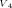
**. Moreover, formation of **10
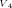
** has been demonstrated in the reaction of [V_4_O_10_]^.+^ with C_3_H_8_ in an elegant combined experimental/computational study including infrared spectroscopic characterization of the ionic species.18d

In line with our experiments (Table 1), formation of the OAT product **4** has been calculated to be endothermic by 14, 12, and 16 kJ mol^−1^ for heteronuclear [V_3_PO_10_]^.+^ and [V_2_P_2_O_10_]^.+^ and homonuclear [P_4_O_10_]^.+^, respectively. In contrast, OAT is exothermic by −103 kJ mol^−1^ and thus accessible for the homonuclear vanadium [V_4_O_10_]^.+^/C_2_H_6_ system under thermal conditions. However, the ODH reaction channel for this system is even more exothermic by 183 kJ mol^−1^, and the highest barrier of this pathway, that is, **TS12**-**15
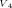
**, has been calculated to be about 83 kJ mol^−1^ lower in energy compared to the exit channel of the oxygen-atom transfer. Accordingly, consistent with theory and our experimental results,^19^ oxidative dehydrogenation corresponds to the main reaction channel in the reaction of [V_4_O_10_]^.+^ with C_2_H_6_.

## Conclusion

We have analyzed the reactions of various hetero- and homonuclear phosphorus-vanadium oxygen-cluster ions [V_*x*_P_4−*x*_O_10_]^.+^ (*x*=0, 2–4) with ethene and ethane using DFT. These calculations, conducted at the B3LYP/aug-cc-pVTZ//B3LYP/TZVP level of theory, reveal some interesting aspects concerning the underlying reaction mechanisms which are operative and which explain the puzzling reactivity patterns observed in the gas phase under ambient conditions. While [V_4_O_10_]^.+^ reacts with ethene exclusively in terms of oxygen-atom transfer to the hydrocarbon, the presence of already one phosphorus atom inhibits this pathway due to the strong P–O^.^ bond. In contrast, [P_4_O_10_]^.+^ is much more reactive with respect to homolytic C–H bond cleavage to form the closed-shell HAT product [P_4_O_9_(OH)]^+^. Further, while the first C–H bond scission is initiated by an active P–O^.^ site, the presence of a vanadium atom in the cluster is essential to enable the second hydrogen-atom transfer from the hydrocarbon, that is, oxidative dehydrogenation. Obviously, while the presence of vanadium is crucial a direct interaction of the redox-active transition metal with the hydrocarbon is not required to bring about ODH reactivity under thermal conditions. Thus, cooperative effects of V and P atoms in the cluster are operative in the occurrence of ODH; neither [V_4_O_10_]^.+^ nor [P_4_O_10_]^.+^ alone possess the electronic properties necessary to bring about the dehydrogenation of ethene.

The redox activity of the P and V atoms, that is, accessibility of the oxidation state +IV, is also of importance in the reaction of the ionic cluster with ethane. Due to the preferred oxidation state +V, [P_4_O_10_]^.+^ is much more reactive in terms of homolytic C–H bond activation during which the formal oxidation state does not change, in contrast to OAT in which a phosphorus atom is necessarily reduced. On the other hand, vanadium can be reduced from +V to +IV and can therefore easier stabilize the radical site formed in the course of reductive processes. Furthermore, the +V→+IV reduction of vanadium is associated with rather small structural rearrangements, causing only a insignificant elongation of the internal V–O bonds of the cluster; accordingly, [V_4_O_10_]^.+^ favors the formation of the open-shell products [V_4_O_9_]^.+^ and [V_4_O_10_H_2_]^.+^. Recalling the initial questions, some crucial aspects about the intrinsic features of the mixed-systems with respect to C–H bond activation have been revealed based on these characteristic features of phosphorus and vanadium. Thus, the combination of both elements gives rise to new product distributions and illustrates the cooperative effects between different metals and non-metals in complex oxo-frameworks. Although the charge of the gas-phase clusters can affect the energetics of the investigated processes compared to the neutral systems,^28^ the investigation of model systems can help to elucidate reaction mechanisms and give hints with respect to the structure of reactive intermediates in heterogeneous catalysis;^4^, ^29^ these insights should be considered relevant for real catalytic systems.

## Computational Methods

All calculations were performed by using the Gaussian09 package.^30^ Geometries were optimized at the unrestricted UB3LYP level of theory^31^ with the triple-ζ plus polarization basis sets TZVP.^32^ B3LYP was shown previously to describe vanadium-oxide clusters in good agreement with available experimental data as well as quantum chemical methods that explicitly include electron correlation.^33^ For example, B3LYP reproduced experimental and CCSD(T) results for the dissociation of [VO_2_]^+^ into [VO]^+^ and 1/2 O_2_;29c good agreement between multi-reference calculations and B3LYP results has also been found for the molecular structures of V_2_O_4_ and the relative energies of its open shell singlet and triplet.^34^ Thus, this method proved reliable in numerous studies of cationic oxo-cluster ions, containing vanadium and/or phosphorus.9d, 18c, 20a,b, 29c, 33–35 Vibrational frequency analyses have been carried out at the same level of theory to characterize the nature of stationary points as minima or transition structures, and to derive the zero-point energy corrections (ZPE). Further, the energy of all stationary points was computed also by single-point calculations, using the larger, correlation-consistent basis set aug-cc-pVTZ of Dunning and coworkers, to derive more accurate values for the apparent activation barriers.^36^ All relative energies (corrected for ZPE contributions) and Gibbs free energies (at STP) are reported in kJ mol^−1^. Intrinsic reaction coordinate (IRC)^37^ calculations or manual displacement along the reaction trajectory of the imaginary frequency were performed to link transition-state structures with the respective intermediates.

## Supplementary material

As a service to our authors and readers, this journal provides supporting information supplied by the authors. Such materials are peer reviewed and may be re-organized for online delivery, but are not copy-edited or typeset. Technical support issues arising from supporting information (other than missing files) should be addressed to the authors.
