# Supplementary material for: Gas-Phase Reactions of Cationic Vanadium-Phosphorus Oxide Clusters with C2Hx (x=4, 6): A DFT-Based Analysis of Reactivity Patterns
Source: Chemistry. 2013 Jan 15;19(9):3017–28. doi: 10.1002/chem.201203050 (PMC3743165; doi:10.1002/chem.201203050)
Supplement: Supplementary file 1 [file chem0019-3017-SD1.pdf]

CHEMISTRY
A EUROPEAN JOURNAL

Supporting Information

© Copyright Wiley-VCH Verlag GmbH & Co. KGaA, 69451 Weinheim, 2013

Gas-Phase Reactions of Cationic Vanadium-Phosphorus Oxide Clusters with C_2H_x ($x = 4, 6$): A DFT-Based Analysis of Reactivity Patterns

Nicolas Dietl,[a] Xinhao Zhang,[a, c] Christian van der Linde,[b] Martin K. Beyer,[b]
Maria Schlangen,*[a] and Helmut Schwarz*[a, d]

chem_201203050_sm_miscellaneous_information.pdf

Geometries and Structures:

Phosphorous = yellow
Vanadium = green
Oxygen = red
Carbon = gray
Hydrogen = white

Structures of $[P_4O_{10}]^{•+}$ 5

1_{P4}: 5
2_{P4}: 6
3_{P4}: 7
4_{P4}: 8
5_{P4}: 9
6_{P4}: 10
7_{P4}: 11
9_{P4}: 12
10_{P4}: 13
11_{P4}: 14
12_{P4}: 15
13_{P4}: 16
14_{P4}: 17
16_{P4}: 18
TS2-3_{P4}: 19
TS7-9_{P4}: 20
TS12-13_{P4}: 21
TS12-16_{P4}: 22

Structures of $[V_2P_2O_{10}]^{•+}$ 23

1_{V2P2}: 23
2_{V2P2}: 24
2_{V2P2}-VO: 25
3_{V2P2}: 26

3_{V2P2}-VO: 27
4_{V2P2}: 28
4_{V2P2}-VO: 29
5_{V2P2}: 30
6_{V2P2}: 31
7_{V2P2}: 32
8_{V2P2}: 33
8_{V2P2}-2: 34
9_{V2P2}: 35
9_{V2P2}-2: 36
10_{V2P2}: 37
10_{V2P2}-2: 38
11_{V2P2}: 39
12_{V2P2}: 40
13_{V2P2}: 41
14_{V2P2}: 42
15_{V2P2}: 43
16_{V2P2}: 44
17_{V2P2}: 45
18_{V2P2}: 46
19_{V2P2}: 47
TS2-3_{V2P2}: 48
TS2-3_{V2P2}-VO: 49
TS2-5_{V2P2}: 50
TS7-8_{V2P2}: 51
TS7-8_{V2P2}-2: 52
TS8-9_{V2P2}: 53
TS8-9_{V2P2}-2: 54
TS12-13_{V2P2}: 55
TS12-15_{V2P2}: 56
TS15-16_{V2P2}: 57
TS12-17_{V2P2}: 58
TS17-18_{V2P2}: 59
TS18-19_{V2P2}: 60
Structures of $[V_3PO_{10}]^{•+}$ 61
1_{V3P}: 61

2_{V3P}: 62
2_{V3P}-VO: 63
3_{V3P}: 64
3_{V3P}-VO: 65
4_{V3P}: 66
4_{V3P}-VO: 67
5_{V3P}: 68
6_{V3P}: 69
7_{V3P}: 70
8_{V3P}: 71
9_{V3P}: 72
10_{V3P}: 73
11_{V3P}: 74
12_{V3P}: 75
13_{V3P}: 76
14_{V3P}: 77
15_{V3P}: 78
16_{V3P}: 79
TS2-3_{V3P}: 80
TS2-3_{V3P}-VO: 81
TS2-5_{V3P}: 82
TS7-8_{V3P}: 83
TS8-9_{V3P}: 84
TS12-13_{V3P}: 85
TS12-15_{V3P}: 86
TS15-16_{V3P}: 87
Structures of $[V_4O_{10}]^{\bullet+}$ 88
1_{V4}: 88
2_{V4}: 89
3_{V4}: 90
4_{V4}: 91
5_{V4}: 92
6_{V4}: 93
7_{V4}: 94
8_{V4}: 95
9_{V4}: 96

10_{V4}: 97
12_{V4}: 98
13_{V4}: 99
14_{V4}: 100
15_{V4}: 101
16_{V4}: 102
$TS2\text{-}3_{V4}$: 103
$TS2\text{-}5_{V4}$: 104
$TS7\text{-}8_{V4}$: 105
$TS8\text{-}9_{V4}$: 106
$TS12\text{-}13_{V4}$: 107
$TS12\text{-}15_{V4}$: 108
$TS15\text{-}16_{V4}$: 109

Structures of $[P_4O_{10}]^{\bullet+}$

1_{P4}:

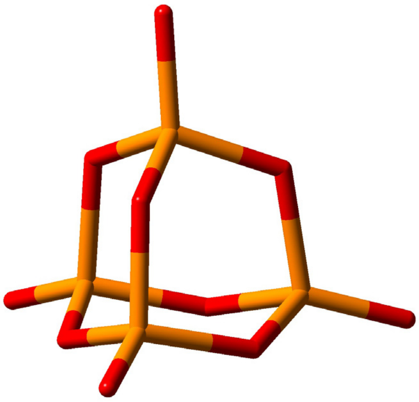

charge = 1, multiplicity = 2

P	0.81483600	-0.66830700	1.46199700
P	0.81483600	-0.66830700	-1.46199700
P	-1.71230500	-0.49898200	0.00000000
P	0.10735300	1.66013200	0.00000000
O	1.46383700	-1.15281700	-2.66450800
O	1.46383700	-1.15281700	2.66450800
O	-3.13028400	-0.82911400	0.00000000
O	0.11903800	3.22911400	0.00000000
O	1.39621600	-1.10340200	0.00000000
O	0.81483600	1.01841700	1.26812300
O	-0.79074700	-0.94119700	1.26487000
O	0.81483600	1.01841700	-1.26812300
O	-1.40717200	1.18359100	0.00000000
O	-0.79074700	-0.94119700	-1.26487000

2_{P4}:

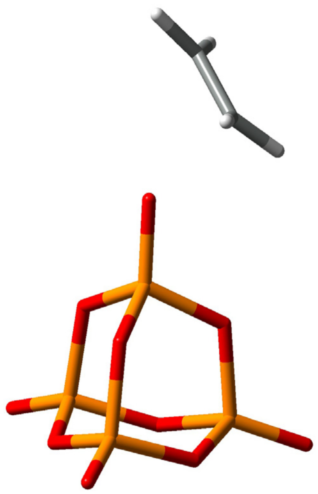

charge = 1, multiplicity = 2

P	0.78762100	-0.74851400	1.60076900
P	-1.13793800	0.07233200	-0.36460400
P	1.39134700	-0.95769500	-1.24110700
P	1.21828400	1.66030600	0.02053300
O	0.93371000	-1.36801700	2.90684200
O	-2.59351500	0.13966000	-0.68798300
O	-0.79318400	-0.59458800	1.06272200
O	1.83436900	0.60594600	-1.06556600
O	2.04637300	-1.75231500	-2.26519100
O	1.72941000	3.01965800	0.03486700
O	-0.41242300	1.50622700	-0.31401000
O	-0.26114900	-0.77566900	-1.41354600
O	1.30415400	0.78835700	1.40462900
O	1.45441100	-1.48887600	0.30688700
C	-5.13033400	0.51662800	-0.04609500
H	-5.65489600	0.75749500	-0.96259600
H	-5.22719600	1.19041900	0.79640200
C	-4.31688200	-0.62991700	0.03553600
H	-3.91474300	-0.93868800	0.99184600
H	-4.34682000	-1.36898600	-0.75336600

3_{P4}:

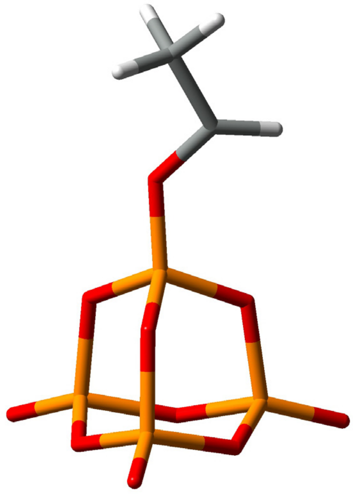

charge = 1, multiplicity = 2

P	0.90952900	-0.37117900	1.71540700
P	1.05920200	1.66283600	-0.37538500
P	1.37835600	-1.16274300	-1.05901300
P	-1.14139300	-0.14065200	-0.24708600
O	1.44301600	3.02380600	-0.69812300
O	1.17388500	-0.67742700	3.10821400
O	2.02899900	-2.11360600	-1.93938500
O	1.27665200	1.11780100	1.14902200
O	-0.71745700	-0.44527400	1.25872000
O	1.55086700	-1.32932800	0.55695000
O	-0.59015200	1.32271200	-0.55606000
O	-0.31237400	-1.14042800	-1.15718700
O	1.67653700	0.43260700	-1.25559600
O	-2.62986800	-0.31697200	-0.53349500
C	-3.76268700	0.47665800	-0.03102200
H	-3.65766000	1.51829300	-0.30271100
C	-5.00477700	-0.29152300	0.05015000
H	-4.90068000	-1.17629700	0.68243900
H	-5.77698000	0.35336700	0.47312200
H	-5.34615800	-0.62124400	-0.94094200

4_{P4}:

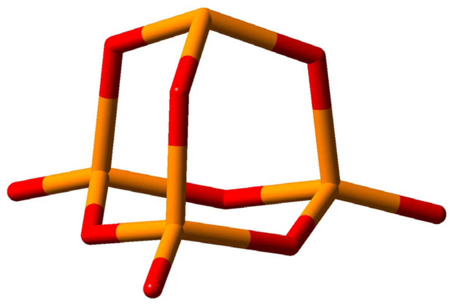

charge = 1, multiplicity = 2

P	0.00000000	1.69152200	-0.42142200
P	-1.46490100	-0.84576100	-0.42142200
P	1.46490100	-0.84576100	-0.42142200
P	0.00000000	0.00000000	1.88206800
O	-2.66301300	-1.53749100	-0.85925200
O	0.00000000	3.07498300	-0.85925200
O	2.66301300	-1.53749100	-0.85925200
O	-1.26623000	0.73105800	-0.80010300
O	0.00000000	1.48042800	1.27322800
O	1.26623000	0.73105800	-0.80010300
O	-1.28208800	-0.74021400	1.27322800
O	1.28208800	-0.74021400	1.27322800
O	0.00000000	-1.46211600	-0.80010300

5$_{P4}$:

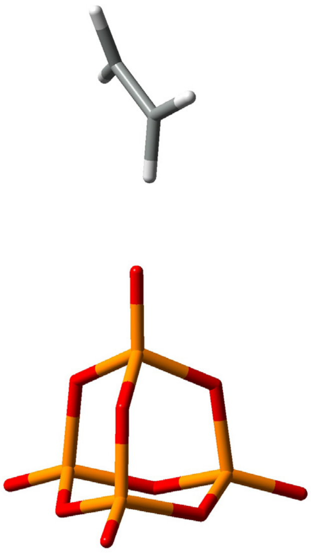

charge = 1, multiplicity = 2

P	-1.07551000	-0.04371100	-0.02490400
P	1.31438000	-1.17242700	-1.17022700
P	1.22657300	1.63754200	-0.43762900
O	-0.38849500	-0.39381700	1.40120900
O	1.72122100	-1.37635400	0.40164600
O	-0.33699800	-1.06653400	-1.04287500
O	1.64568100	1.07371300	1.04050500
O	1.69624700	0.40180800	-1.40162500
O	-0.41353300	1.38625500	-0.40366800
O	-2.54684700	-0.07973900	-0.04526700
O	1.81439400	-2.14165100	-2.13227700
O	1.70513500	-0.73738600	2.97214000
O	1.65318200	2.98052800	-0.79767200
C	-5.44142900	-0.61603600	0.02958500
H	-4.34378100	-0.55341500	-0.04912800
H	-5.90115100	-1.59904800	0.13358900
C	-6.21155100	0.52969100	-0.01496300
H	-7.21698100	0.52731200	-0.43557900
H	-5.82578200	1.47717100	0.36017800
P	1.25493500	-0.40197200	1.63051200

6_{P4}:

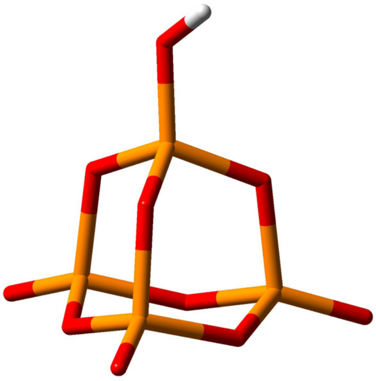

charge = 1, multiplicity = 1

P	-0.02064100	1.79630500	-0.15820900
P	0.92267300	-0.42829300	1.49440600
P	0.88551400	-0.68640000	-1.41879100
P	-1.53202900	-0.58537900	0.07121000
O	1.59157400	-0.81117700	2.72203700
O	-3.00148300	-1.03299200	0.13079700
O	1.52340300	-1.27949000	-2.57743600
O	-0.11481900	3.23677000	-0.28501700
O	0.66900100	0.93082100	-1.36066600
O	-0.72420700	-1.17741900	-1.16068000
O	-1.51365200	0.98792900	-0.06758000
O	0.70199500	1.15491000	1.15986700
O	1.48862100	-0.99004600	0.06853800
O	-0.69159800	-0.95352000	1.36671300
H	-3.26344600	-1.96977300	0.19815200

7$_{P4}$:

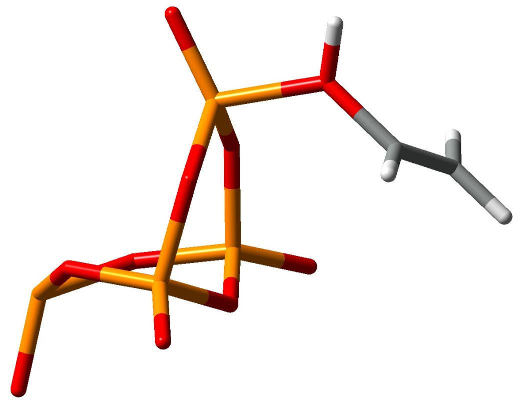

charge = 1, multiplicity = 2

P	1.41240900	-0.65348500	1.11323000
P	-0.63252700	-1.20542800	-0.95805000
P	-2.82972900	0.01299300	0.70846100
O	0.07429300	2.86672200	-0.41455100
O	2.99462400	-0.31853900	0.35109700
O	0.75426900	0.78311200	1.00322200
O	-1.76012100	-1.19599300	0.19160800
O	-0.72814100	-2.06563300	-2.12834400
O	-3.98139200	0.24726600	-0.20878800
O	1.75278400	-1.23437100	2.40716900
O	0.74260100	-1.51843100	-0.04404000
O	-1.72963600	1.30266900	0.74617100
O	-0.41300400	0.39858400	-1.21821300
C	3.27090900	0.06782600	-1.05078200
H	3.28629700	-0.79694500	-1.69549200
C	3.44539000	1.34099100	-1.31048900
H	3.40170900	2.11459100	-0.55446200
H	3.64484000	1.63551000	-2.33384900
H	3.74049100	-0.72381800	0.83658600
P	-0.35157700	1.52556600	-0.03482800

9_{P4}:

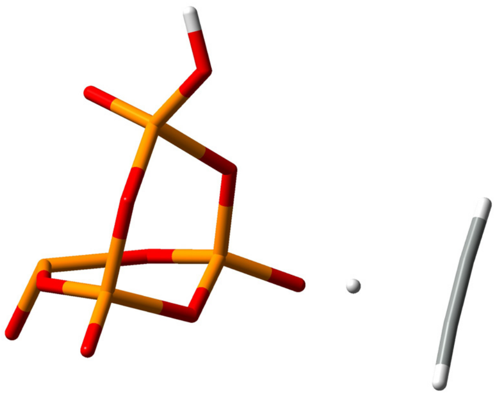

charge = 1, multiplicity = 2

P	-0.23989000	1.95962700	-0.63846900
P	-1.02356700	0.18465000	1.57479600
P	-2.04868700	-1.35171100	-0.89707200
O	2.13191900	-1.49232000	-0.58346700
O	0.59126700	3.28125500	-0.47457400
O	1.00798000	0.82339100	-0.80555700
O	-2.06307000	-0.50351000	0.54276900
O	-1.33799700	0.18768700	2.99469900
O	-2.58138500	-2.73577200	-0.80433400
O	-1.28455500	1.76638900	-1.65437300
O	-0.68213200	1.62750000	0.90062300
O	-0.31973700	-1.38059500	-1.17555900
O	0.37730500	-0.65023700	1.15490300
C	4.90762100	-0.59049900	-0.14081500
H	5.20022500	-0.17088800	-1.07730800
C	4.64044800	-1.06625700	0.93032700
H	3.04214500	-1.20656600	-0.21997800
H	4.45948000	-1.49100000	1.89262600
H	0.35096700	4.00488900	-1.07673000
P	0.84161100	-0.69831400	-0.37370200

10_{P4}:

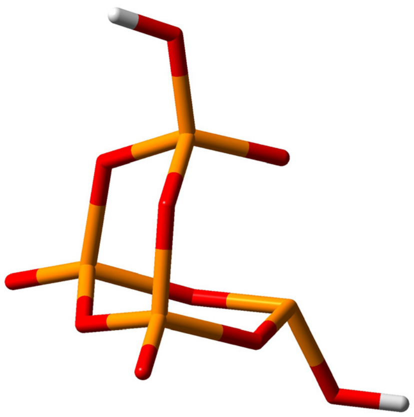

charge = 1, multiplicity = 2

P	0.11930200	-1.42757800	1.00103200
P	1.51722900	0.00017800	-1.19736400
P	0.06201800	1.48762100	0.93156400
P	-1.59544500	-0.04825700	-0.83788900
O	3.11158900	-0.06644300	-1.29364100
O	-3.08979600	-0.08669300	-1.25559800
O	0.16362100	2.70613600	1.71547800
O	0.27453400	-2.60331600	1.83885400
O	0.22756000	0.04950800	1.69072700
O	-1.35181600	1.24562600	0.12029500
O	-1.30382000	-1.28201800	0.18392600
O	1.22650100	-1.26067200	-0.22363400
O	1.18270500	1.30408900	-0.27750200
O	-0.60393500	-0.05816900	-1.97462100
H	-3.78954900	-0.08443200	-0.58006500
H	3.54583200	0.32058200	-2.07435900

11_{P4}:

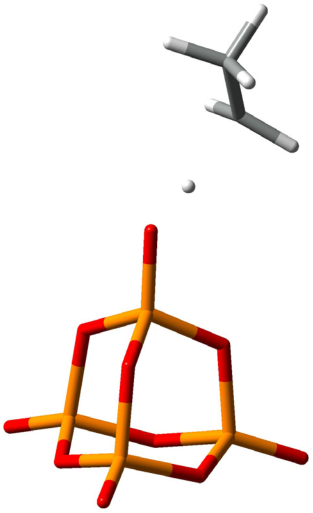

charge = 1, multiplicity = 2

P	0.84529100	-1.26626600	-1.25307600
P	-0.97619700	0.34825200	0.23810500
P	1.51966300	1.48402200	-0.53729300
P	1.39671600	-0.57326400	1.52840000
O	-0.72728400	-0.78381700	-0.86302200
O	1.50896500	0.19574800	-1.54680300
O	-0.13381100	1.61390900	-0.24042700
O	1.40776900	-1.58926400	0.24546200
O	1.99653900	0.79421800	0.86523400
O	-0.24163700	-0.17908700	1.55477200
O	-2.43100600	0.70324800	0.45305500
O	2.16985100	2.70495900	-0.97444300
O	0.93129400	-2.29622300	-2.27179300
O	1.94189700	-1.03991600	2.78938500
C	-4.72771800	-0.62114800	-0.00452200
C	-5.71669600	0.47751900	0.01648200
H	-3.32468300	0.10690700	0.24767100
H	-4.65309700	-1.27662100	0.86113100
H	-4.45963000	-1.06943100	-0.95936600
H	-6.71602900	0.03029200	-0.13456600
H	-5.76139300	0.99583800	0.97526800
H	-5.58139700	1.19343100	-0.79528900

12_{P4}:

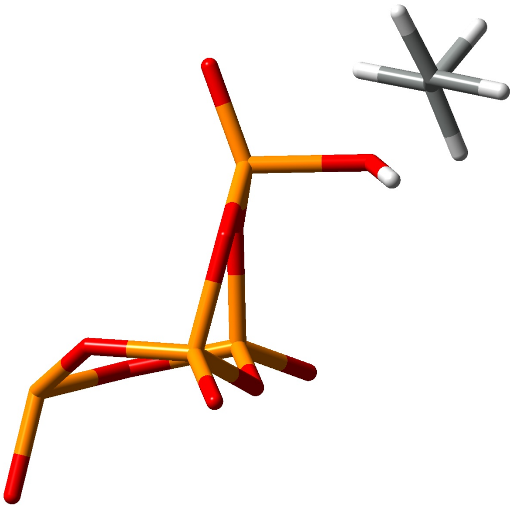

charge = 1, multiplicity = 2

P	1.42751900	-0.59884300	0.76747700
P	-1.05457000	-1.39276400	-0.64550300
P	-2.80825100	0.44892100	0.98709700
O	0.06502700	2.57458200	-1.28229500
O	2.63277000	-0.61362100	-0.53940000
O	0.76745800	0.82810500	0.54258600
O	-1.94851600	-0.96652800	0.62030400
O	-1.42860600	-2.49281900	-1.52272600
O	-4.11631200	0.57172900	0.28361300
O	2.10013100	-0.86841600	2.03387400
O	0.43680200	-1.65397600	0.09533800
O	-1.65926700	1.57610500	0.46426600
O	-0.74308000	0.05706200	-1.34418000
C	4.11911300	-0.14456300	-0.28050500
H	4.60049100	-0.46037600	-1.20255700
C	4.13412800	1.33351400	-0.05871900
H	3.71892100	1.88114600	-0.90404800
H	5.18558800	1.61936500	0.04302800
H	2.59753100	-1.42700000	-1.07687300
P	-0.40092900	1.42748300	-0.51377400
H	3.63364800	1.62423200	0.86464800
H	4.43657300	-0.74080500	0.57066600

13_{P4}:

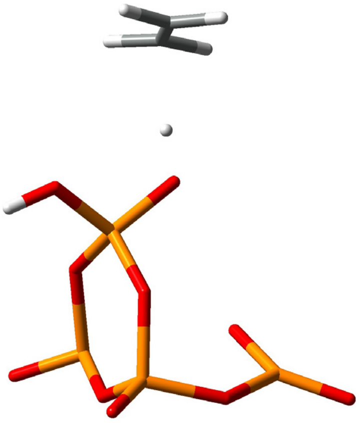

charge = 1, multiplicity = 2

P	0.61322700	-1.61981500	-1.48453100
P	1.63823000	-0.41041700	1.14623700
P	-1.31212200	-0.42304000	0.53801400
P	1.89273100	1.95526200	-0.59032400
O	2.21067600	-0.99017200	2.35457500
O	2.66949100	3.19107500	-0.63552500
O	-2.28333600	-1.22021500	1.48105900
O	0.68537700	-3.02294600	-1.96563500
O	-0.89308000	-1.36173600	-0.65918300
O	-2.01692000	0.88693600	0.14158100
O	0.83339600	1.28672100	-1.38319200
O	1.63114900	-1.31880000	-0.18716000
O	0.01978000	-0.00369200	1.28902300
O	2.27045600	1.01808700	0.69357100
H	-2.06627100	-2.13513800	1.72526100
C	-4.81925900	1.14023700	-0.94720500
H	-5.04342600	0.14110000	-1.30513400
H	-4.53270800	1.87128600	-1.69520600
C	-4.96656100	1.47313200	0.33737100
H	-5.31142500	0.75719200	1.07529300
H	-3.02438800	0.94571400	0.00091900
H	-4.80376700	2.48771200	0.68402900

14_{P4}:

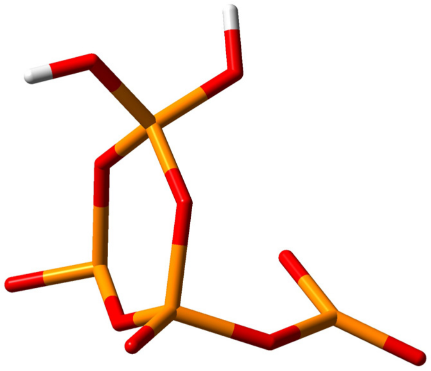

charge = 1, multiplicity = 2

P	0.87326100	1.71925300	-0.88448300
P	-0.54746000	0.31161600	1.45474500
P	1.56598500	-1.14171300	-0.12134800
P	-2.44158000	-0.31341400	-0.71307900
O	-0.48935200	0.47584700	2.90072100
O	-3.85655300	-0.64156200	-0.85605000
O	2.87551600	-1.66647200	0.55931800
O	1.77009500	2.90198200	-0.86024400
O	1.83589200	0.26055600	-0.77333100
O	1.11423000	-2.25816300	-1.11187100
O	-1.24823400	-0.14046400	-1.57602000
O	0.01522300	1.51569200	0.53955900
O	0.36589200	-0.98733500	0.88957600
O	-2.00443700	-0.05859100	0.84379200
H	3.42734900	-1.06744700	1.09025000
H	1.79338200	-2.78061600	-1.57136600

16$_{P4}$:

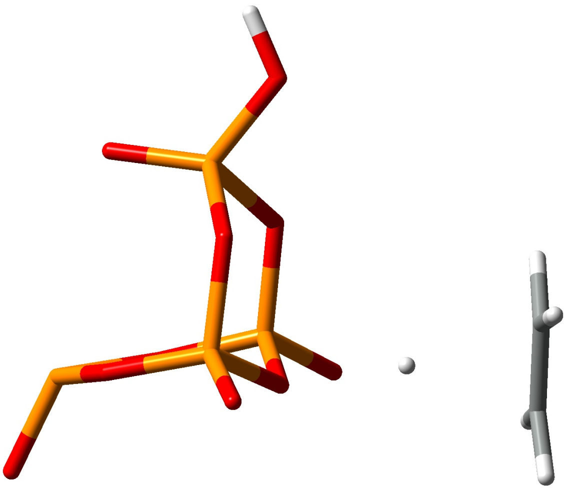

charge = 1, multiplicity = 2

P	-0.59293400	1.93022400	-0.64638300
P	-1.06346300	0.08473500	1.59671700
P	-1.94125600	-1.56466500	-0.85733000
O	2.23154900	-1.15650100	-0.64730300
O	0.04000300	3.36100200	-0.51534600
O	0.79944600	0.98564300	-0.83604600
O	-2.02712500	-0.75343900	0.60372100
O	-1.33366600	0.05903800	3.02559500
O	-2.29776300	-3.00485200	-0.77962700
O	-1.62568200	1.57580200	-1.63132600
O	-0.94560800	1.55613000	0.90600500
O	-0.22872500	-1.37571900	-1.17211400
O	0.42773400	-0.55032800	1.14045400
C	5.00297700	-1.11552700	0.36471400
H	5.39586500	-1.66808400	-0.48162400
C	4.73376100	0.19101300	0.28507000
H	3.12187100	-0.81512600	-0.25832800
H	4.91846800	0.75380200	-0.62356300
H	-0.31475200	4.03358200	-1.12006000
P	0.85466900	-0.54484000	-0.40023100
H	4.40248900	0.75426100	1.15117300
H	4.87910400	-1.66737000	1.28999000

$TS2\text{-}3_{P4}$:

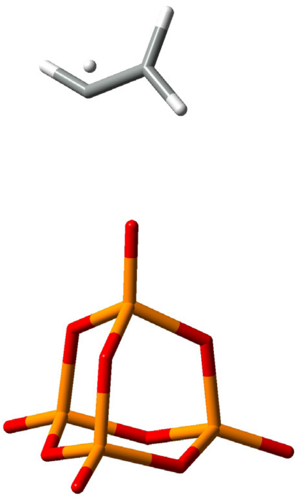

charge = 1, multiplicity = 2

P	-1.25621100	-0.42222200	1.60714400
P	1.12632100	-0.11685800	0.01433400
P	-1.10268600	1.65998300	-0.41283000
P	-1.27789200	-1.12812300	-1.21095900
O	-1.75083300	-0.76826900	2.93025300
O	2.59675100	-0.21404800	0.02760700
O	0.39101300	-0.47017700	1.41598800
O	-1.59494900	0.46510100	-1.41538500
O	-1.46767700	3.02663200	-0.75144300
O	-1.79133400	-2.05496800	-2.20698100
O	0.37248600	-1.08648700	-1.04382000
O	0.52680400	1.34688800	-0.34717100
O	-1.72950800	-1.35043500	0.34589800
O	-1.57686000	1.08031800	1.04175800
C	5.53487200	-0.55945900	-0.05689900
H	6.52304700	-0.75855700	-0.48506900
H	4.87250700	-1.40318000	0.13934900
C	5.02369700	0.69242200	-0.01889300
H	5.71081900	-0.02771800	1.03832100
H	5.39207000	1.70353600	-0.17680300

$TS7\text{-}9_{P4}$:

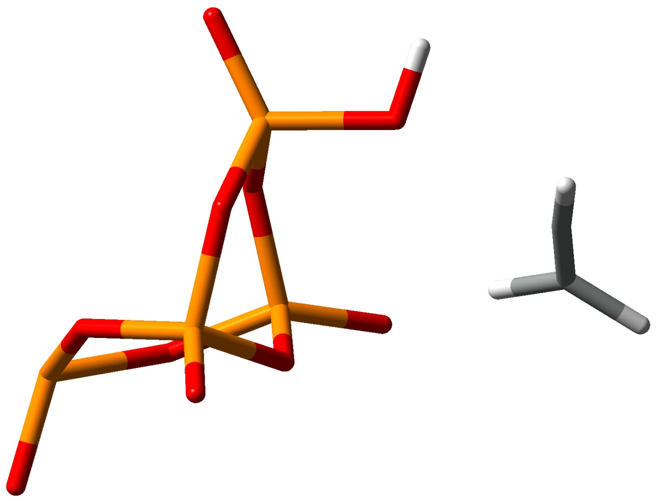

charge = 1, multiplicity = 2

P	-1.33972500	-1.29109900	-0.86562500
P	0.69458000	-0.91910700	1.24475600
P	2.82710400	0.17842300	-0.64086400
O	-0.71753300	2.52512300	-0.51605800
O	-2.72151600	-0.66461100	-0.21598500
O	-0.54646500	0.05655600	-1.34125900
O	1.91351500	-0.96887900	0.17534500
O	0.90606400	-1.33588100	2.62492400
O	3.95266100	0.77148200	0.13311900
O	-1.56573000	-2.29709000	-1.90225800
O	-0.50112400	-1.71627500	0.45397500
O	1.60074300	1.33680100	-0.95788500
O	0.17094300	0.63590000	1.01878500
C	-3.63188100	1.09001000	1.20387400
H	-4.10754400	0.19798500	1.56255700
C	-3.13857800	2.20685900	0.92427500
H	-2.25928100	2.38811800	0.23218200
H	-3.53291400	3.10749900	1.41352300
H	-3.48086600	-1.03042700	-0.70202500
P	0.08943500	1.28582400	-0.47538400

TS12-13$_{P4}$:

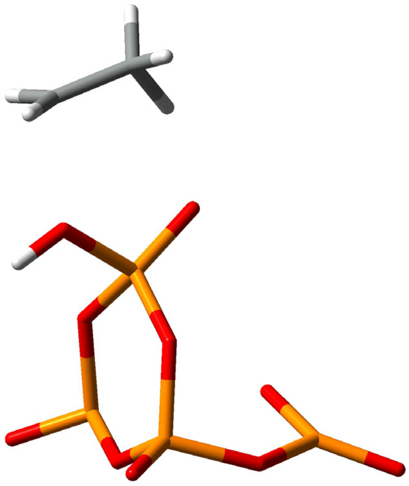

charge = 1, multiplicity = 2

P	-2.51522100	-1.41362400	-0.66633500
P	-0.66244900	2.03588400	-0.90613700
P	1.38344300	-0.10304200	-0.23953200
O	-0.54123300	3.51745700	-0.89918200
O	-1.15452900	0.30777200	2.87834000
O	2.69600500	0.30449200	0.67231700
O	-3.55197200	-2.43967500	-0.76182800
O	1.80693300	-1.15763700	-1.17736200
O	0.27122700	-0.52896300	0.84672800
O	-2.23314400	-0.95031800	0.87196500
O	-1.62827800	-0.66012600	-1.58302400
O	0.88246800	1.31449200	-0.77748100
O	-1.32701400	1.43494000	0.51525600
H	2.58847200	1.03827700	1.29901700
C	4.75477500	-0.50179700	0.34253200
H	4.85940700	-0.41529500	1.41821800
H	4.94286800	0.39460000	-0.23798200
C	4.61208300	-1.75892800	-0.30488800
H	4.65068200	-2.62840300	0.34424100
H	3.62317200	-1.73119400	-0.84028500
P	-1.13401700	0.11975600	1.43010000
H	5.29420400	-1.81769900	-1.16634900

TS12-16$_{P4}$:

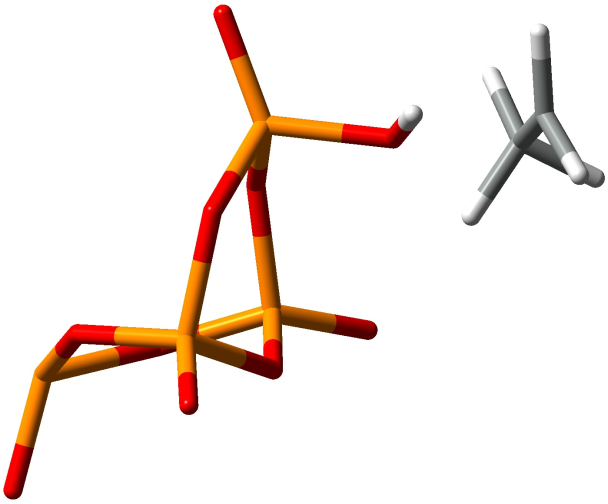

charge = 1, multiplicity = 2

P	1.26437200	-1.20521200	0.81475000
P	-1.19088200	-1.23586900	-0.83703400
P	-2.74041400	0.75484400	0.86081700
O	0.86027100	2.37173400	-0.69696800
O	2.42468900	-1.03835900	-0.35749000
O	0.79506500	0.34449500	0.91514400
O	-2.17194500	-0.73780700	0.35339800
O	-1.71461100	-2.05576000	-1.92203000
O	-3.91699200	1.28000800	0.11239400
O	1.69463700	-1.84998500	2.05443700
O	0.04393600	-1.91214200	0.01077000
O	-1.33780800	1.68975300	0.59027200
O	-0.49051500	0.19819500	-1.27201800
C	4.17335100	0.57160300	-0.67592000
H	4.02984400	0.32054900	-1.72225400
C	3.80916700	1.84995100	-0.20082400
H	2.74743000	2.09694100	-0.46858900
H	4.28329700	2.57311300	-0.90120600
H	2.76362000	-1.90366400	-0.64362300
P	0.00133500	1.29498800	-0.17669600
H	4.04117000	2.06806700	0.83605900
H	4.72955000	-0.13664500	-0.07075500

Structures of $[V_2P_2O_{10}]^{\bullet+}$

$\mathbf{1}_{V2P2}$:

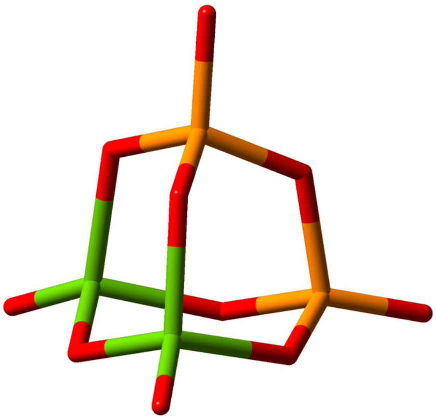

charge = 1, multiplicity = 2

P	0.00000000	1.21204900	-1.48836200
P	0.00000000	1.08204400	1.42209500
V	1.53494400	-0.98746800	-0.01071100
V	-1.53494400	-0.98746800	-0.01071100
O	0.00000000	2.19330200	-2.56433000
O	0.00000000	2.04874900	2.68878200
O	0.00000000	1.91912700	0.06477200
O	0.00000000	-1.91097800	0.00571600
O	2.83627000	-1.82424400	-0.11570200
O	-2.83627000	-1.82424400	-0.11570200
O	-1.28860200	0.18893700	1.44327500
O	1.28860200	0.18893700	1.44327500
O	-1.26758200	0.19846400	-1.33212200
O	1.26758200	0.19846400	-1.33212200

2_{V2P2}:

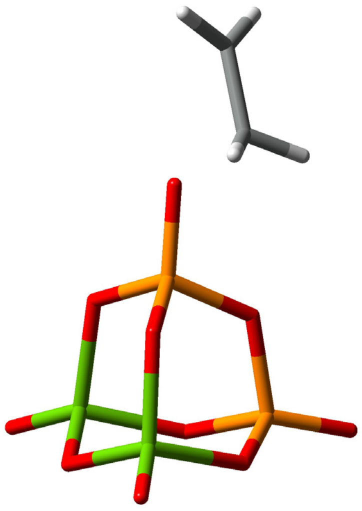

charge = 1, multiplicity = 2

P	-0.57706700	-0.02952100	1.93036100
P	1.29860700	-0.22083500	-0.31897600
V	-1.03141900	1.61246200	-0.59755100
V	-1.40177500	-1.40445700	-0.66671200
O	-0.56831800	-0.06034300	3.38890600
O	2.78965600	-0.46195700	-0.56403800
O	0.96645000	-0.20288900	1.25781700
O	-1.91157300	0.20470800	-1.26883800
O	-1.50282600	3.01542200	-1.07568800
O	-2.20600500	-2.62287600	-1.20109400
O	0.43057600	-1.37637400	-0.96482800
O	0.74462900	1.15797200	-0.89102600
O	-1.38280400	-1.18910400	1.12114100
O	-1.07507400	1.32700400	1.17915900
C	5.23637400	-0.34827800	-0.40527300
H	5.69365600	-0.15486000	-1.36533700
H	5.64060700	-1.13223500	0.21949000
C	4.05163100	0.37814300	0.00385500
H	3.87556000	0.39790500	1.07519300
H	3.92485300	1.34874300	-0.46565300

2$_{V2P2}$**-VO:**

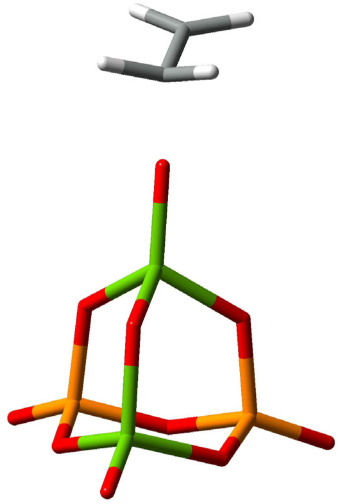

charge = 1, multiplicity = 2

P	1.01352000	-1.09361300	-1.48633600
V	1.50576500	1.50166800	-0.00062900
O	1.32729100	-1.94097400	-2.63172800
O	-2.96400600	0.38606800	-0.00053400
O	-0.58500100	-0.64731900	-1.32924500
O	1.73274500	0.36732500	1.37088600
O	2.41327800	2.76412700	-0.00094300
O	1.32721300	-1.93842200	2.63352800
O	-0.58496600	-0.64592100	1.32960400
O	-0.33630100	1.71300500	-0.00081500
O	1.28465500	-1.73551100	0.00081100
O	1.73269600	0.36596600	-1.37112500
C	-5.22097100	-0.47376000	0.00023200
H	-5.47508700	-0.96234500	0.93135800
H	-5.47487900	-0.96245100	-0.93089500
C	-4.46006300	0.76617000	0.00017200
H	-4.56686000	1.36610800	-0.90062100
H	-4.56613400	1.36585300	0.90120900
V	-1.28906300	0.26875800	-0.00027600
P	1.01342500	-1.09226600	1.48725700

3_{V2P2}:

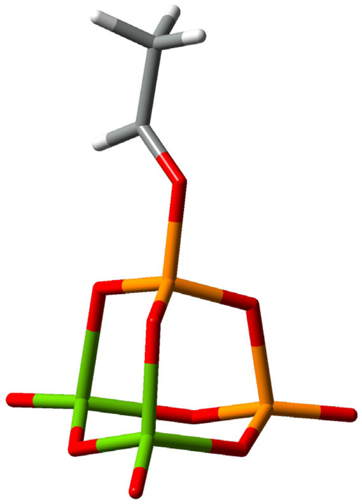

charge = 1, multiplicity = 2

V	0.83810500	1.91929100	-0.18848600
V	1.39822100	-1.07655200	-1.15534500
P	-1.17764700	-0.33027800	-0.08794600
P	0.94922800	-0.51120500	1.85273200
O	2.27078800	-1.94699200	-2.11545700
O	1.09630100	-1.02437500	3.21296300
O	-2.86604700	-0.71852300	-0.13537900
O	0.80310400	3.46280800	-0.43928500
O	-1.07402200	1.16467500	-0.37790200
O	-0.78450000	-0.74057000	1.36213700
O	1.11428300	1.00103700	1.47255500
O	1.59681000	0.60176600	-1.38877700
O	-0.54609100	-1.25558600	-1.15014800
O	1.62625400	-1.33693400	0.63902900
C	-3.78635800	0.14371400	-0.24807600
H	-3.49184700	1.19427800	-0.33398100
C	-5.17527400	-0.25992700	-0.26225600
H	-5.68936200	0.27707600	0.55035700
H	-5.63811900	0.14116300	-1.17675500
H	-5.31513900	-1.33443700	-0.17919600

3_{V2P2}-VO:

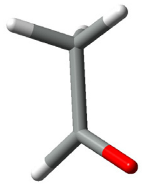

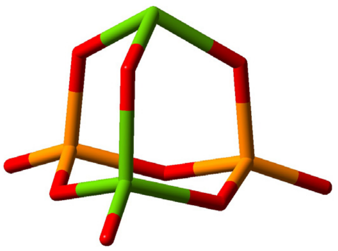

charge = 1, multiplicity = 2

V	-1.68319500	-1.38368400	0.00745300
P	-1.28483300	1.44253500	-1.02805900
O	-2.74324500	-2.52014300	0.07553100
O	-1.82292600	2.54920300	-1.81339200
O	3.10568600	-0.42743600	-0.68358600
O	-0.19897200	1.32119600	3.03404800
O	1.00284600	0.20534600	0.96951100
O	0.18321600	0.84316400	-1.51178400
O	-0.99466600	1.73665400	0.55769200
O	-1.31776000	-0.58880000	1.56385700
O	0.03457500	-1.77227000	-0.61958600
O	-2.14426000	0.04949500	-0.96416600
C	3.95185100	-0.13117700	0.18104300
H	3.59013700	0.15465800	1.17762800
C	5.38888800	-0.14979100	-0.08277000
H	5.79307700	0.84593300	0.14487400
H	5.86660200	-0.81648600	0.64767000
H	5.62848300	-0.44649200	-1.10049600
V	1.11551400	-0.44012700	-0.72284400
P	-0.36196800	0.73910300	1.70336900

4$_{V2P2}$:

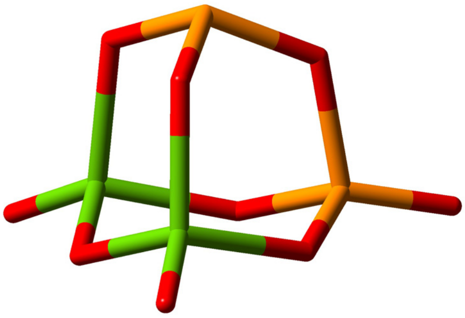

charge = 1, multiplicity = 2

P	0.00017800	1.80534600	-0.54925300
P	0.00051500	0.29908600	1.96568300
V	1.53271100	-0.83220800	-0.30632400
V	-1.53325100	-0.83190600	-0.30553800
O	0.00028500	3.19278900	-1.00296300
O	0.00077500	1.69400700	1.16855000
O	-0.00057500	-1.64868000	-0.74074100
O	2.83164700	-1.52475700	-0.79568900
O	-2.83178600	-1.52412700	-0.79641900
O	-1.28404500	-0.52533900	1.53075700
O	1.28425400	-0.52656900	1.52986500
O	-1.26685200	0.85079800	-0.89447900
O	1.26655000	0.85039900	-0.89558400

4_{V2P2}-VO:

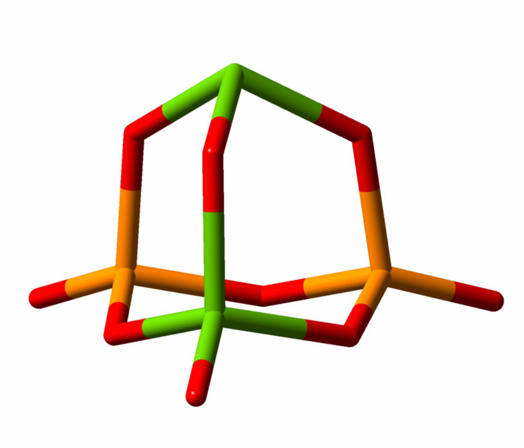

charge = 1, multiplicity = 2

V	1.69024100	0.00018800	-0.41827900
P	-0.94922100	1.48944600	-0.57899300
O	3.12195900	0.00048700	-1.01499900
O	-1.70083800	2.63844700	-1.06611500
O	-1.70056400	-2.63814300	-1.06717300
O	-0.88060300	-1.32050600	1.09214000
O	-0.88096000	1.31983300	1.09258900
O	-1.50840300	0.00017300	-0.97977300
O	0.64038200	-1.36744300	-0.91579300
O	1.45711700	-0.00028500	1.44155300
O	0.64014200	1.36768100	-0.91555300
V	-0.16988400	-0.00032900	1.98520300
P	-0.94905100	-1.48936100	-0.57929100

5_{V2P2}:

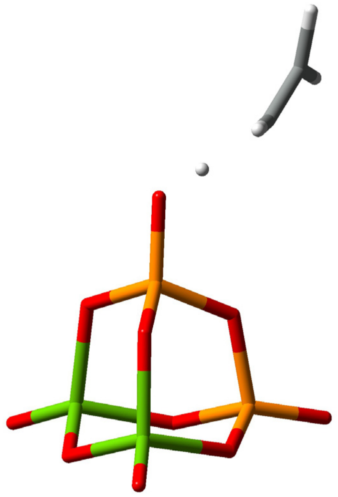

charge = 1, multiplicity = 2

P	-0.32947700	-0.30813300	1.89967400
P	1.12656100	-0.31011400	-0.64226800
V	-0.99895900	1.73215400	-0.26392200
V	-1.70264400	-1.19851100	-0.67691900
O	1.06059200	-0.50748100	0.95160200
O	-0.78899400	1.18570600	1.43760800
O	0.64770600	1.17772800	-0.92243200
O	-1.37574500	-1.26015400	1.09341600
O	-2.12078200	0.52116200	-0.95769800
O	0.05255800	-1.27893200	-1.27893500
O	2.52029200	-0.64425200	-1.18130100
O	-1.38850500	3.22524500	-0.45595600
O	-0.09163100	-0.56182900	3.31627600
O	-2.70990100	-2.24774800	-1.22501800
C	4.86116600	0.50320700	-0.38179400
H	3.40732500	-0.18427400	-0.84967100
H	4.91853800	1.49968800	-0.80173600
C	5.68625100	-0.42285700	0.02409200
H	6.73996800	-0.39437800	-0.26791200
H	5.38556900	-1.27876100	0.62330500

6$_{V2P2}$:

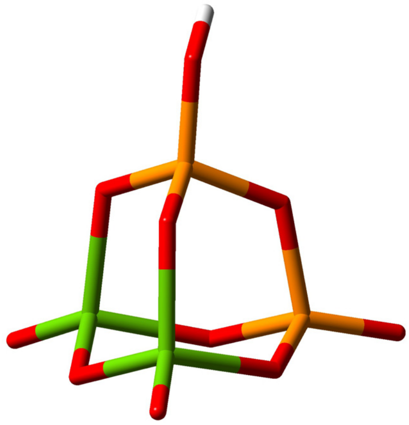

charge = 1, multiplicity = 1

V	-1.53071100	-0.99408900	-0.10106800
V	1.52124500	-1.00217600	-0.16406000
P	-0.03083600	1.28686200	-1.21984200
P	0.04276900	0.93737800	1.67650100
O	2.83305700	-1.82753800	-0.25862400
O	-0.14343300	2.42923600	-2.26599300
O	0.07230500	1.73806400	2.89356100
O	-2.84542600	-1.81889400	-0.13007000
O	-1.23414400	-0.03092400	1.38920800
O	0.01603200	1.88689500	0.26136200
O	-1.30626500	0.38131300	-1.34947900
O	-0.01028200	-1.92225500	-0.29000700
O	1.30211300	-0.03950300	1.33972600
O	1.25234500	0.38368200	-1.39287100
H	0.54831300	3.10989200	-2.32643400

7_{V2P2}:

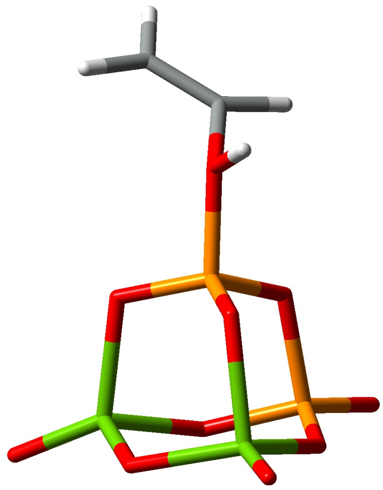

charge = 1, multiplicity = 2

V	0.65187800	1.73718000	-0.65695800
P	-1.11225700	-0.51767000	-0.30156500
V	1.79699800	-1.25619200	-0.57831300
O	0.92920700	3.17604900	-1.19923300
O	-2.71720100	-1.12176000	-0.60149700
O	-1.08246800	0.88042000	-0.95636700
O	1.48741000	-0.87603200	1.28028400
O	0.32942400	0.20712300	3.43259700
O	2.63092300	-2.41984900	-1.20362700
O	-0.21514600	-1.57363000	-0.92452900
O	-1.05223500	-0.42118100	1.25388200
O	1.73258000	0.56236000	-1.25284700
O	0.66007500	1.51689200	1.15809600
H	-2.74938500	-2.06782200	-0.85112100
C	-3.94704500	-0.60121100	0.08826100
H	-3.96852700	-0.92927100	1.11504300
C	-4.74908300	0.14818700	-0.62266100
H	-5.64381800	0.53207000	-0.14653300
H	-4.56607700	0.40176100	-1.65867100
P	0.52291700	0.13604000	1.98588700

8$_{V2P2}$:

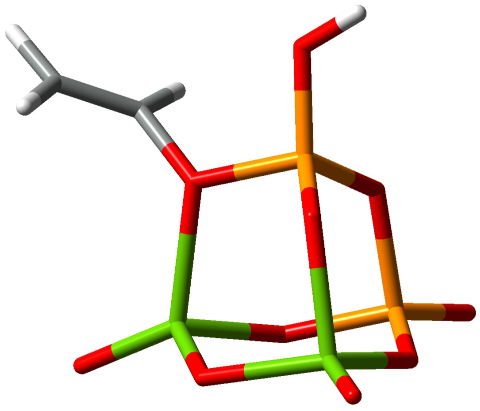

charge = 1, multiplicity = 2

V	-0.67239600	1.42292300	0.69645500
P	-0.61679500	-1.25828700	-0.87070800
V	1.89687500	0.62096200	-1.00978300
O	-1.44066200	2.63411200	1.31847500
O	-1.59860500	-2.24925800	-1.57712700
O	-1.67575000	-0.03709400	-0.37273600
O	2.10304800	-0.45936800	0.55575500
O	1.35271700	-1.89119000	2.68560700
O	2.94430000	0.94812400	-2.11939500
O	0.42524200	-0.57008500	-1.72758100
O	-0.05681800	-1.86671900	0.45866000
O	0.55783500	1.89390600	-0.35955200
O	0.05165600	0.25658500	1.86761800
H	-1.24358600	-3.06001400	-1.97853500
C	-3.10063500	-0.28835000	-0.11305100
H	-3.24552600	-0.99861400	0.68713200
C	-3.99185700	0.36999500	-0.81431000
H	-5.04052100	0.21735100	-0.59255700
H	-3.73035500	1.06312000	-1.60338700
P	1.04000800	-1.00792400	1.56539200

8_{V2P2}-2:

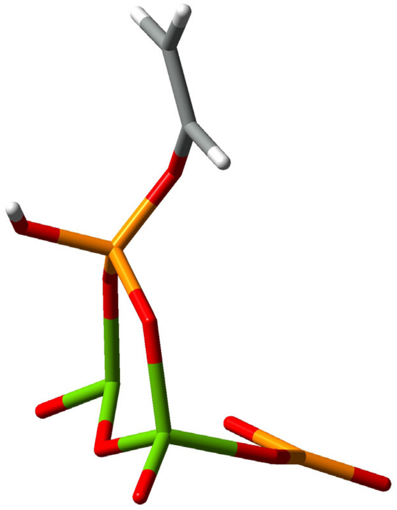

charge = 1, multiplicity = 2

P	-2.52935100	-0.86166200	-1.22536000
P	1.90026300	0.32427800	0.05585000
V	-0.60183200	-1.06316200	1.26466800
V	-0.97802200	1.78580700	-0.09614600
O	-3.46896800	-1.49755900	-2.14580100
O	2.83845100	1.01829400	1.12559000
O	2.70911500	-0.17844100	-1.20183900
O	-1.09189500	0.54494300	1.39062800
O	-0.81280700	-1.80612200	2.62050200
O	-1.45804300	3.18771500	0.37874500
O	0.88647300	1.37558900	-0.42905800
O	1.17924400	-0.91971600	0.72287500
O	-2.15972200	0.62148700	-1.20532600
O	-1.73937000	-1.66402800	-0.15979700
C	3.78667200	-1.11168300	-1.11323900
H	3.47139500	-2.09917000	-0.80556600
C	4.99715200	-0.74934600	-1.48037800
H	5.22274600	0.25363600	-1.81785800
H	5.79361700	-1.48093100	-1.46751000
H	3.52242500	0.47526500	1.54712100

9_{V2P2}:

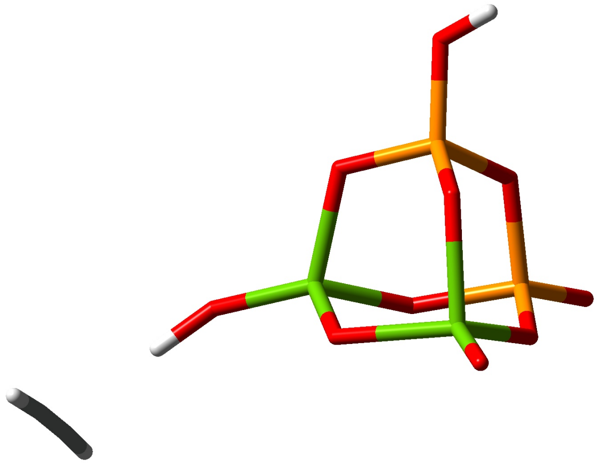

charge = 1, multiplicity = 2

V	1.28520400	-0.30560100	0.16008400
P	-1.36436300	-0.72372300	1.37286700
V	-1.08959100	1.87773500	-0.25138800
O	2.94239900	-0.63572200	0.25131800
O	-1.98821100	-1.48646700	2.58528900
O	0.21174600	-0.93338800	1.50195700
O	-1.48877200	0.54065700	-1.54124000
O	-1.63926400	-1.92263200	-2.57135900
O	-1.64127500	3.33470900	-0.18089700
O	-1.69916200	0.77126200	1.28149300
O	-1.78257100	-1.46665200	0.01430000
O	0.83206800	1.27827300	0.04798800
O	0.42731200	-1.07071900	-1.16402000
C	5.75701400	-0.10825100	-0.47942900
H	5.91000800	-0.81131300	-1.26705900
C	5.62941100	0.69103700	0.40923700
H	3.87532700	-0.24834400	0.17591700
H	5.57878800	1.41102400	1.19423500
H	-2.92481700	-1.33275900	2.78999100
P	-1.21237800	-1.00620800	-1.51757200

9_{V2P2}-2:

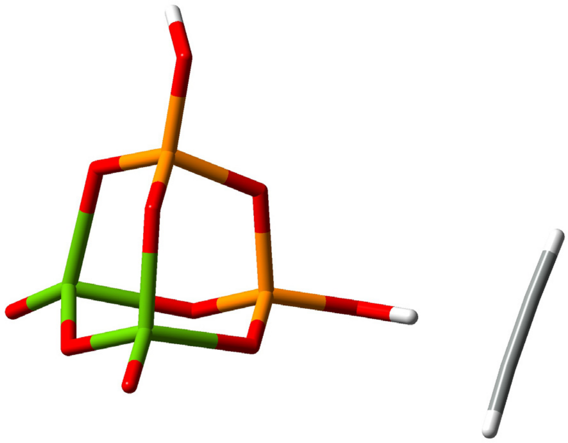

charge = 1, multiplicity = 2

P	-1.09634600	-0.65106900	-0.54015300
P	0.54771400	-0.44031700	1.76577900
V	0.42032900	1.86683700	-0.11704600
V	1.98482200	-0.80867200	-0.93628000
O	-2.44184800	-1.30830200	-0.92056100
O	0.44393900	-0.78765900	3.28658400
O	-0.84416700	-0.95383300	1.07056700
O	1.68625100	1.11045800	-0.96786500
O	0.41541200	3.42139400	-0.25658800
O	2.92085200	-1.59972900	-1.89936800
O	1.71947500	-1.10184100	1.01752700
O	0.53684500	1.11684800	1.61756200
O	0.08437600	-1.25158200	-1.30204800
O	-1.12440400	0.92689300	-0.62688300
C	-5.21167700	-0.58977800	0.02544100
H	-5.42731600	-1.36678400	0.72316300
C	-5.02113300	0.29566700	-0.76371500
H	-3.31511100	-0.85920800	-0.71578500
H	-4.90711100	1.09193200	-1.46362800
H	0.78356600	-1.64945200	3.57657200

10_{V2P2}:

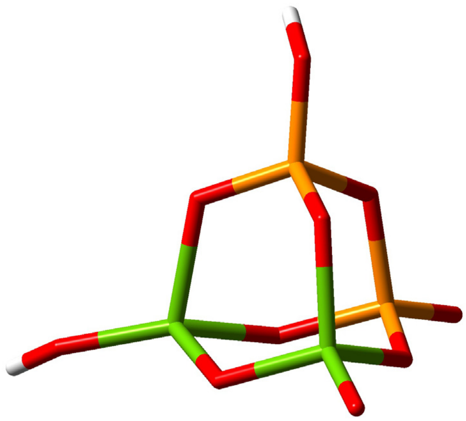

charge = 1, multiplicity = 2

V	0.89459800	1.60235000	-0.15062800
V	-1.86193500	0.13331100	0.15231700
P	0.50381800	-1.05206400	-1.33041000
P	0.72674200	-0.90894600	1.56003600
O	-3.55064800	0.33891200	-0.08311300
O	0.98015600	-1.96820900	-2.49756800
O	1.19172100	-1.69549000	2.69706800
O	1.58478200	2.99316600	-0.25081200
O	1.33946700	0.55283000	1.27159600
O	0.98859000	-1.67138600	0.07137100
O	1.19852500	0.34239100	-1.50613700
O	-0.85490700	1.64792500	-0.04842500
O	-0.88681500	-0.62947100	1.43616800
O	-1.04645300	-0.88472800	-1.24367300
H	0.67062900	-2.88802400	-2.53525800
H	-4.43561100	0.17548200	0.28020600

10_{V2P2}-2:

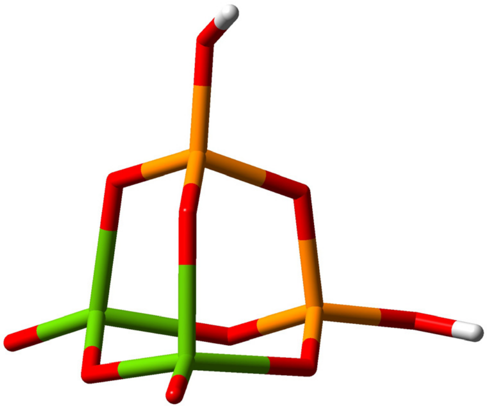

charge = 1, multiplicity = 2

V	1.21756300	1.41646000	-0.00965900
V	-1.87950800	0.59669100	-0.25038700
P	0.42815800	-1.15432900	-1.30798800
P	0.19107100	-0.91014600	1.50453100
O	-3.39478000	0.92835500	-0.10371900
O	0.94680200	-2.21699300	-2.32703300
O	0.48399800	-1.80056800	2.75257900
O	2.20128800	2.62543300	-0.03615700
O	1.23066900	0.26053800	1.47763300
O	0.55910000	-1.82928500	0.18501000
O	1.42166100	0.05227000	-1.30605300
O	-0.41630600	1.86841400	-0.17206000
O	-1.25844900	-0.44065000	1.34278000
O	-1.03885400	-0.72087100	-1.48732300
H	0.30603400	-2.84025500	-2.70578600
H	-0.25075400	-2.30821900	3.13342000

11_{V2P2}:

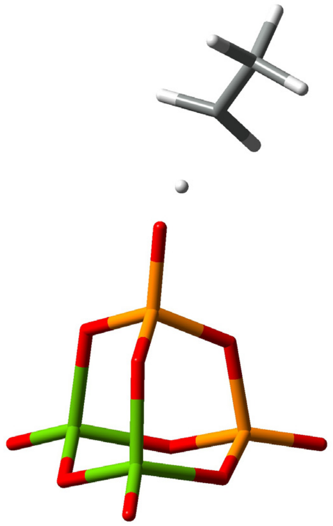

charge = 1, multiplicity = 2

V	1.65692400	-1.36911700	-0.55051100
V	1.20364300	1.63893400	-0.48220800
P	0.42807500	-0.06739700	1.92427600
P	-1.08773300	-0.25121200	-0.57434700
O	1.70796600	3.06034800	-0.85968600
O	-2.52247800	-0.52571600	-1.04385300
O	0.20482200	-0.12959600	3.36433000
O	2.56256000	-2.55557200	-0.98354400
O	1.36819400	-1.19267700	1.21950000
O	-1.00071000	-0.26142300	1.02958800
O	-0.11508600	-1.37416400	-1.11199200
O	2.20626100	0.26504700	-1.04134600
O	0.99453700	1.31544000	1.27686800
O	-0.49986300	1.14683000	-1.04124500
C	-4.85899300	0.61380200	-0.07009400
H	-4.50308300	0.87795300	0.92265300
H	-3.35971500	-0.04949300	-0.64215600
C	-5.82219800	-0.50133900	-0.21599100
H	-5.58677100	-1.35193400	0.42555700
H	-6.81870500	-0.14720200	0.09800200
H	-5.93033600	-0.83493400	-1.24917900
H	-4.86204200	1.41603500	-0.80372800

12$_{V2P2}$:

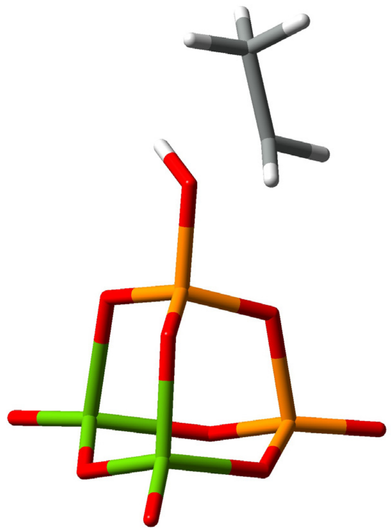

charge = 1, multiplicity = 2

V	1.64944500	1.55546400	-0.34930500
V	1.01915200	-1.53771800	-0.90245000
P	-1.10334400	0.31834900	-0.26905700
P	0.61825800	-0.39685200	1.94018700
O	1.52934300	-2.81510800	-1.64464800
O	0.43853600	-0.71708200	3.35516000
O	-2.76912000	0.65774000	-0.50801200
O	2.28094100	2.92048600	-0.77372800
O	-0.38451500	1.58557400	-0.70074700
O	-1.01590400	-0.00437500	1.25915900
O	1.40977600	0.85234100	1.42288000
O	1.89028800	-0.12493900	-1.28818000
O	-0.83148300	-0.94849700	-1.11409800
O	0.98792200	-1.59873600	0.92339000
H	-2.97700600	1.60967800	-0.58433200
C	-3.95501400	-0.22725300	0.06768900
H	-3.81473900	-0.16826000	1.14332400
H	-3.69976200	-1.20575400	-0.32815200
C	-5.23554200	0.34330200	-0.44850200
H	-5.42335300	1.35258700	-0.07705800
H	-5.29223400	0.31749700	-1.53626400
H	-6.03729800	-0.29186300	-0.05865200

13_{V2P2}:

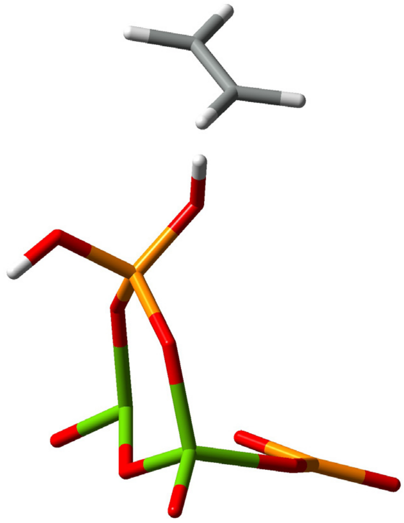

charge = 1, multiplicity = 2

V	-1.30176100	1.67428000	-0.43526400
V	-0.71931800	-0.80182000	1.45649700
P	1.72601800	0.59257700	0.07613700
P	-2.39312900	-1.32334500	-1.16322100
O	-0.94497700	-1.30792400	2.91572000
O	-3.14926400	-2.23509000	-2.01859600
O	2.82086000	1.39010500	0.89964300
O	-1.99832200	3.05803500	-0.27683300
O	0.61840500	1.49862600	-0.51746600
O	2.48865200	-0.08942300	-1.10081300
O	-2.20370400	0.17503100	-1.39765100
O	-1.42262600	0.71698400	1.24706200
O	1.06214600	-0.49508900	1.02041900
O	-1.63188700	-1.80575600	0.09768200
H	2.55430000	1.80527300	1.73354600
C	5.53022600	-0.56917600	-1.27242900
H	5.91022800	0.31459400	-0.77234300
H	5.53447900	-0.55262800	-2.35657000
C	5.13180600	-1.64320800	-0.59032900
H	5.17952500	-1.67900100	0.49258300
H	3.42459600	-0.41695200	-0.96381100
H	4.80190400	-2.54602400	-1.09226600

14$_{V2P2}$:

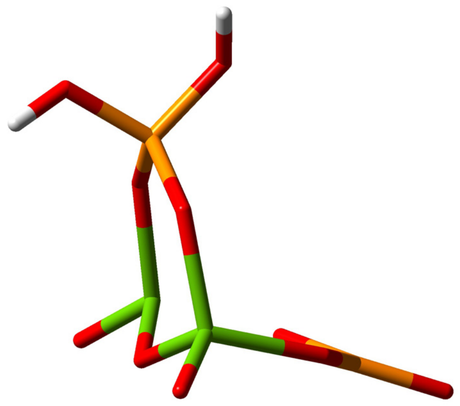

charge = 1, multiplicity = 2

V	0.17757200	-1.43684100	-0.79415800
V	0.23017000	1.72765000	-0.52213200
P	-2.21169900	-0.05232900	0.68674900
P	2.50190700	-0.15139700	0.90071700
O	0.30816400	2.92259500	-1.51548800
O	-3.69946300	-0.06354200	0.15091300
O	3.73115300	-0.30060600	1.67525400
O	0.22110400	-2.56684700	-1.86823600
O	1.69479100	-1.36749800	0.37606800
O	-2.34959700	-0.13256100	2.25447400
O	-1.44071400	-1.31514300	0.13339900
O	0.28555800	0.07428800	-1.53410900
O	1.82025500	1.16983600	0.54321100
O	-1.42189500	1.23400000	0.36572900
H	-3.85594700	-0.19248200	-0.79697300
H	-3.07008500	-0.67639700	2.60993200

15$_{V2P2}$:

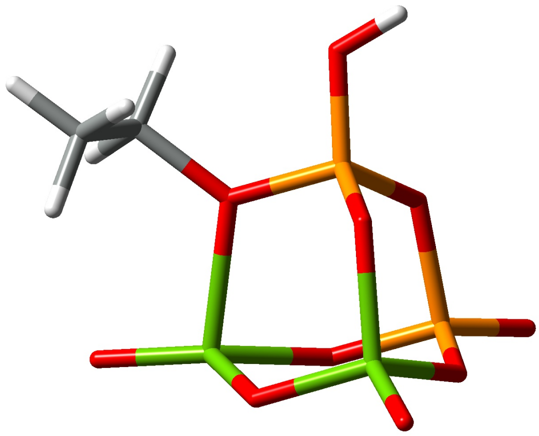

charge = 1, multiplicity = 2

V	1.21437100	-0.93481600	1.32606600
O	2.72188600	1.66093800	-1.84450700
O	-1.83240800	-1.88700100	-1.98746200
O	-1.44655700	2.56781700	1.13523800
O	1.95781200	-1.72190100	2.45042700
O	0.05861500	0.53394500	1.79217100
O	-1.56398400	0.43446200	-0.20538000
O	0.16904500	-1.90537100	0.36958000
O	2.24748500	-0.01865300	0.14295000
O	0.56274200	1.86789900	-0.33946700
O	0.75088200	-0.13620300	-1.90273400
H	-1.03061800	3.30464300	1.61338100
C	-3.11978200	0.56260900	-0.19012100
H	-3.37175900	0.22669700	-1.19105800
H	-3.30361100	1.62726100	-0.08730500
C	-3.70728300	-0.27591400	0.90396600
H	-3.47011600	-1.33312300	0.78529400
H	-3.40774500	0.06320800	1.89586100
H	-4.79454900	-0.17496700	0.83599200
P	-0.54538100	1.38268000	0.64641100
V	-0.63805800	-1.22321900	-1.21548900
P	1.75081100	0.81955200	-1.15074700

16_{V2P2}:

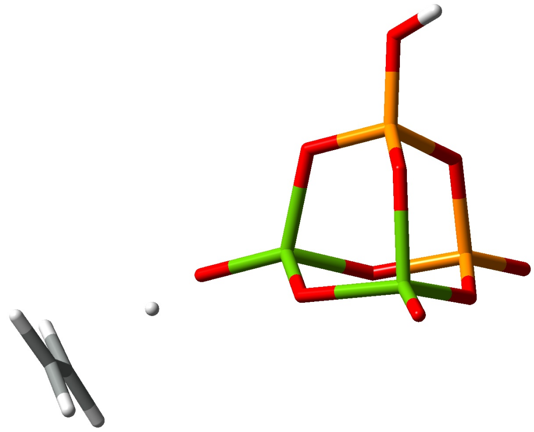

charge = 1, multiplicity = 2

V	-1.17550200	1.87321500	-0.22228300
P	-1.40953800	-0.74734400	1.37506200
O	-1.73045600	-1.90284900	-2.57837900
O	2.87907100	-0.60062600	0.19150600
O	-2.01208300	-1.52913500	2.58665200
O	-1.72152700	3.33126500	-0.12582900
O	-1.75815600	0.74572300	1.30578500
O	0.16915000	-0.94584600	1.48151600
O	0.75151400	1.29026700	0.04315300
O	-1.58558400	0.54948200	-1.52293400
O	-1.84089900	-1.47780900	0.01381900
O	0.34918500	-1.05092200	-1.19094500
H	-2.94870400	-1.38907400	2.80056400
C	5.78175200	-0.28912900	-0.38394900
H	5.74452500	-0.26708800	-1.46738500
H	6.06858800	-1.22953800	0.07307800
C	5.53947300	0.80188600	0.34902200
H	5.30755400	1.75563400	-0.11213300
H	3.82617300	-0.22498300	0.12550600
H	5.63187300	0.79162800	1.42941400
P	-1.29454800	-0.99524800	-1.52042100
V	1.21907700	-0.29280800	0.13124600

17$_{V2P2}$:

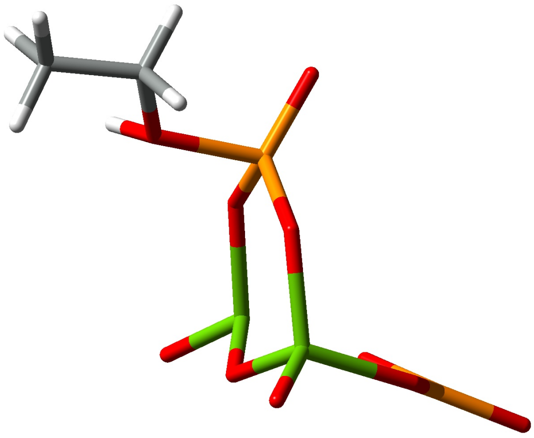

charge = 1, multiplicity = 2

P	3.08767100	-0.32887900	0.70098500
P	-1.54999900	0.15819200	1.06063300
V	0.50731800	-1.42373800	-0.72192700
V	0.82577100	1.71847100	-0.50540900
O	4.38188600	-0.55562800	1.33978300
O	-3.02207500	0.31017600	-0.00107700
O	-2.02553400	0.06988000	2.44695100
O	0.65850600	0.06686700	-1.50004900
O	0.30194800	-2.56540100	-1.76772200
O	0.90610600	2.90507100	-1.51250000
O	-0.78246100	1.40295000	0.51709100
O	-0.93887700	-1.14947500	0.40134200
O	2.45408500	1.03566400	0.42506700
O	2.16241200	-1.48790900	0.25385100
C	-4.28225400	-0.49357000	0.34045700
H	-3.93888900	-1.52078100	0.25655100
C	-5.35835400	-0.13445200	-0.64389900
H	-5.06442700	-0.35638800	-1.66954000
H	-6.23817800	-0.73734400	-0.40741800
H	-3.23698800	1.24168500	-0.18862600
H	-4.51433500	-0.24806900	1.37382300
H	-5.65761800	0.91290700	-0.56157800

18_{V2P2}:

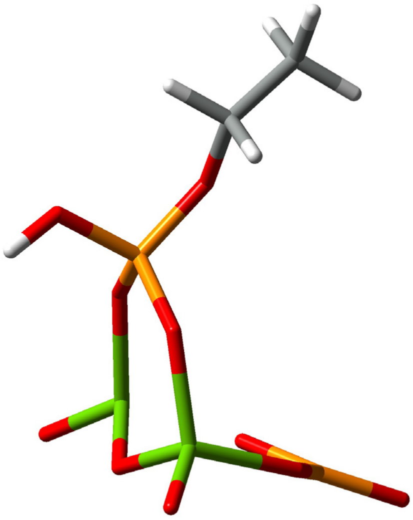

charge = 1, multiplicity = 2

V	1.08797500	-1.43801400	-1.04068400
V	0.74040400	0.18690100	1.65926200
P	-1.84876100	-0.57286600	0.06551900
P	2.27965900	1.59065400	-0.70667000
O	1.08004800	0.12223600	3.18154200
O	3.02669200	2.73466600	-1.22452500
O	-2.88206900	-1.62665600	0.64555500
O	1.72187700	-2.80111600	-1.44747600
O	-0.82138700	-1.19590300	-0.91290800
O	-2.69359600	0.47674200	-0.71397500
O	2.00618000	0.28680100	-1.45553900
O	1.35627500	-1.16624200	0.86210500
O	-1.07680600	0.10290300	1.27906500
O	1.61571600	1.59793900	0.69386400
H	-2.56108300	-2.29292200	1.27102000
C	-3.95872300	1.11017000	-0.21866000
H	-3.71084400	1.64268400	0.69895700
H	-4.65193600	0.29902500	-0.00474800
C	-4.45379800	2.02693400	-1.30390000
H	-3.73452800	2.81743600	-1.51689100
H	-4.66472200	1.47599000	-2.22001100
H	-5.38138000	2.49297900	-0.96468800

19$_{V2P2}$:

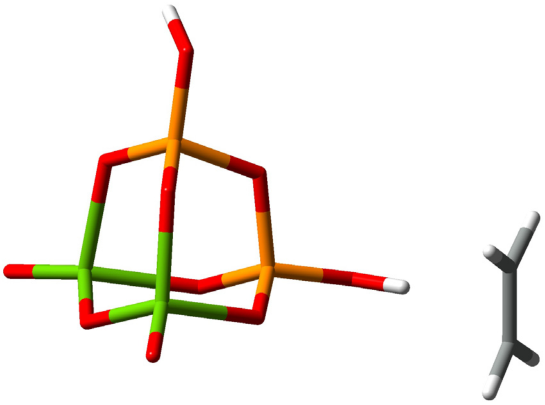

charge = 1, multiplicity = 2

V	1.95049900	-0.88225400	-1.02634000
V	0.47884300	1.85563500	-0.24061000
P	0.69615100	-0.31250700	1.75151400
P	-1.07719400	-0.69874500	-0.43536600
O	0.44929600	3.39434400	-0.50181100
O	-2.45196200	-1.35468300	-0.68705100
O	0.68565300	-0.58821200	3.29035000
O	3.21087200	-1.65474200	-1.52251500
O	1.82419000	-0.98893800	0.96701800
O	-0.73194800	-0.88290400	1.16943100
O	0.05017100	-1.38628700	-1.22360800
O	1.71678000	1.04557100	-1.08128700
O	0.62901400	1.23812600	1.53271400
O	-1.07257000	0.86353500	-0.65565100
H	1.09069500	-1.41420200	3.60019500
C	-5.06872100	-0.19501300	0.41036100
H	-4.75926600	0.66023600	1.00132100
H	-5.31185400	-1.10065300	0.95507600
C	-5.21918700	-0.11224000	-0.91259200
H	-5.02982300	0.80977500	-1.45092600
H	-3.31376200	-0.86749900	-0.49816800
H	-5.58373600	-0.94959700	-1.49720300

TS2-3$_{V2P2}$:

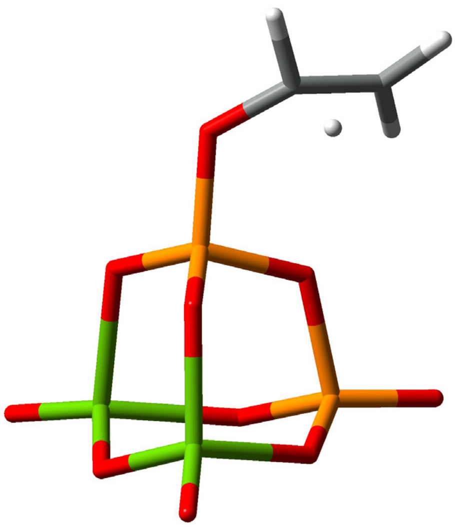

charge = 1, multiplicity = 2

P	0.07391400	0.03178600	1.91807200
P	1.06859400	-0.32116500	-0.81165600
V	-1.02035800	1.68758600	-0.43064500
V	-1.71454500	-1.40876800	-0.26306800
O	0.69225900	0.06782900	3.24664400
O	2.52280000	-0.63188300	-1.47318700
O	1.39410800	-0.26170800	0.75012700
O	-2.10295900	0.41440100	-0.77363400
O	-1.57801900	3.10744600	-0.77872900
O	-2.55319700	-2.65545100	-0.69479500
O	0.09759100	-1.42861900	-1.18103800
O	0.62088400	1.11026800	-1.25405300
O	-0.93523500	-1.07606100	1.46303800
O	-0.48003000	1.41577100	1.30068000
C	4.20082600	-0.36433600	0.27562700
H	5.18502500	0.01953300	0.53955700
H	3.59340200	-0.83830000	1.04502700
C	3.70891000	-0.08838300	-1.01993600
H	3.64513900	1.03656400	-0.77576400
H	4.45752200	-0.06957400	-1.82421100

TS2-3$_{V2P2}$-VO:

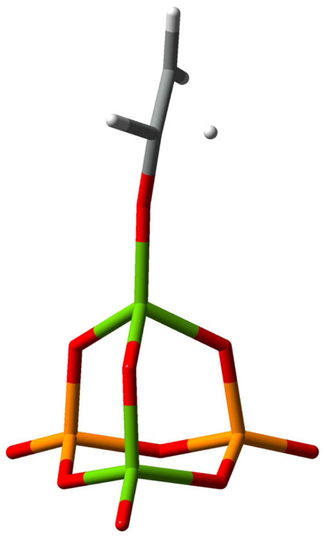

charge = 1, multiplicity = 2

P	-1.23372800	-1.07668000	-1.41734400
P	-0.99504500	-1.01666000	1.53831100
O	-1.68112600	-1.92793400	-2.51739900
O	3.09378700	0.24092200	-0.20483200
O	0.40493000	-0.77150800	-1.37988700
O	-1.62851600	0.47811700	1.44931600
O	-1.28092700	-1.81978900	2.72563700
O	-2.16745700	2.92425600	0.07553800
O	0.40520700	1.65043200	-0.11610400
O	0.60222900	-0.72778700	1.23401800
O	-1.83437800	0.42429100	-1.29546300
O	-1.44249000	-1.67601800	0.09700700
C	5.39544200	-0.41946900	0.03868000
H	6.43767000	-0.11989700	0.08580300
H	5.14726000	-1.47275200	0.13063400
C	4.39301500	0.59785000	-0.03135500
H	4.55859600	1.04296200	0.99798200
H	4.69360500	1.44566900	-0.67349300
V	1.31056900	0.12731200	-0.16179000
V	-1.39345400	1.57155600	0.03387600

TS2-5$_{V2P2}$:

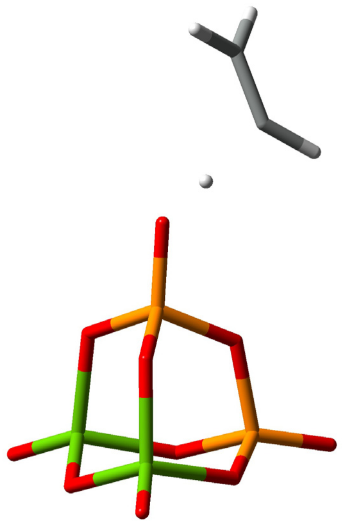

charge = 1, multiplicity = 2

P	0.30935400	-0.13365900	1.90911900
P	-1.15631000	-0.17351500	-0.63395800
V	1.57041700	-1.36262800	-0.58145000
V	1.19164600	1.64477400	-0.40341400
O	-1.09673300	-0.25504600	0.97528600
O	1.24265000	-1.25407800	1.18825500
O	-0.17848600	-1.29841900	-1.18260500
O	0.92884000	1.25897700	1.33754200
O	2.18075400	0.27131600	-0.98997900
O	-0.49740200	1.21939400	-1.02897600
O	-2.58385400	-0.34290400	-1.13156000
O	2.44826400	-2.55911700	-1.04721800
O	0.04491200	-0.24985300	3.33982800
O	1.73546800	3.06853500	-0.71512600
C	-4.80749100	-0.23158700	0.23485400
H	-3.52862700	-0.24722100	-0.50758100
H	-4.62882100	-0.33347300	1.30044100
C	-5.84099300	0.10607700	-0.49995800
H	-6.62191000	0.75654700	-0.09606100
H	-5.94815000	-0.17499400	-1.54531000

TS7-8$_{V2P2}$:

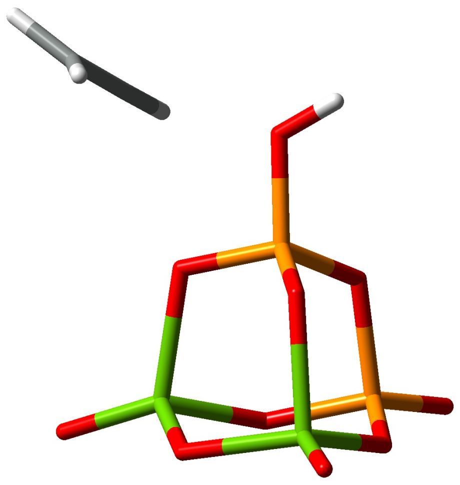

charge = 1, multiplicity = 2

V	-0.16040000	-0.72851400	1.60502700
P	-0.90818400	0.42332300	-0.98076700
V	1.71930100	1.43339100	0.21810900
O	-0.64944400	-1.33360500	2.96849100
O	-2.26171400	0.71523900	-1.80474900
O	-1.52731500	-0.13254600	0.36680900
O	2.16728700	-0.16121400	-0.73902500
O	1.76863600	-2.55336500	-1.85155800
O	2.49564400	2.79227700	0.25950100
O	-0.03661200	1.65827800	-0.77129900
O	-0.13990000	-0.74991700	-1.70864000
O	0.84176900	0.63702300	1.76419100
O	0.68600300	-1.84651700	0.44927200
H	-2.16950800	1.02531100	-2.72046700
C	-4.09676400	-0.13406700	-0.30236900
H	-3.73859000	-1.09545200	-0.62348500
C	-4.69644200	0.88330800	0.13360000
H	-5.72489900	0.69847500	0.48463400
H	-4.30911100	1.89905100	0.20025000
P	1.31430200	-1.45267400	-1.00086000

TS7-8$_{V2P2}$-2:

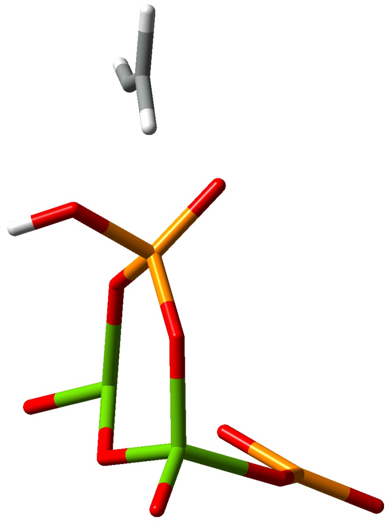

charge = 1, multiplicity = 2

P	2.72377500	-0.44305600	1.25100500
P	-1.80470700	0.13567500	0.32392500
V	0.66613700	-1.35474100	-0.94786300
V	0.92688600	1.74169000	-0.40219300
O	3.76368900	-0.75892300	2.22886400
O	-3.13699500	0.34559100	-0.63865900
O	-2.44955100	-0.08842000	1.65482700
O	1.06100400	0.18003400	-1.53556700
O	0.76831400	-2.42526000	-2.08303300
O	1.29729100	3.01206300	-1.23003400
O	-0.88121900	1.38672700	0.11623600
O	-1.02977800	-1.12730400	-0.27714900
O	2.22917300	0.96010000	0.90160000
O	1.95858500	-1.53822800	0.46987900
C	-4.68192700	0.24871300	0.90014000
H	-4.48361300	1.22819500	1.28471100
C	-5.34349700	-0.81505900	0.68331500
H	-5.01178900	-1.69458200	0.14596000
H	-6.34698400	-0.81348600	1.12391300
H	-3.07474000	0.81780400	-1.48368000

TS8-9$_{V2P2}$:

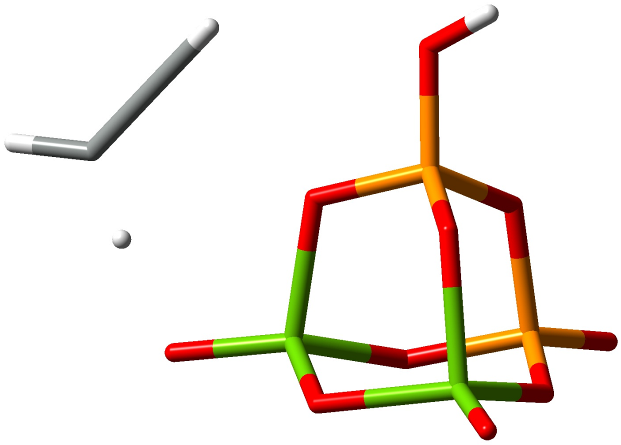

charge = 1, multiplicity = 2

V	-0.94480900	-0.78752100	-0.94622900
P	-0.20176400	0.84928600	1.36425200
V	1.89196200	-1.21546300	0.41857100
O	-2.30314700	-1.40010500	-1.57506800
O	-0.90361800	1.62380600	2.54520900
O	-1.42990300	0.14040100	0.60798400
O	2.16866500	0.32819400	-0.66839700
O	1.55775900	2.60805200	-1.91719100
O	2.96438500	-2.12345600	1.10216100
O	0.83616900	-0.18957900	1.78815700
O	0.40587400	1.89731800	0.33641200
O	0.23084600	-1.87780800	-0.43781000
O	-0.11011900	0.56631600	-1.76676700
C	-4.00041100	0.18133500	0.90508400
H	-3.64055300	0.75640300	1.73616600
C	-4.41484800	-0.48482000	-0.06083200
H	-3.57371800	-0.90411900	-0.75938800
H	-5.42351400	-0.74810700	-0.37455300
H	-0.32620300	2.05454000	3.19604000
P	1.15748000	1.42709400	-1.15392900

TS8-9$_{V2P2}$-2:

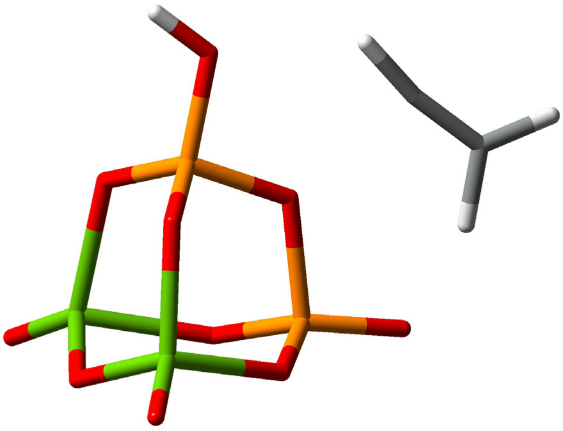

charge = 1, multiplicity = 2

P	-0.85956300	-0.10064500	-1.41714800
P	-0.57935500	-0.02521100	1.50500800
V	1.28169800	1.62038800	-0.13546100
V	1.61588100	-1.53022100	-0.02989300
O	-2.02701100	-0.15495200	-2.32828200
O	-1.62547900	-0.01047100	2.69646000
O	-1.58264200	-0.08133100	0.23007300
O	2.32050900	0.26520700	-0.17217200
O	2.02976300	2.98714400	-0.25360300
O	2.36028900	-2.87722000	-0.29232700
O	0.36317700	-1.23541000	1.50782900
O	0.21079600	1.32254600	1.41355700
O	0.14627700	-1.26034200	-1.29887500
O	-0.05357100	1.27607200	-1.36197900
C	-3.80778300	-0.10559100	0.47615600
H	-3.59938100	-0.06827000	1.52785200
C	-4.26709800	-0.15203100	-0.69314000
H	-3.53856200	-0.16559900	-1.55790900
H	-5.33717400	-0.17860900	-0.90649500
H	-1.27298800	-0.07774200	3.59822100

TS12-13$_{V2P2}$:

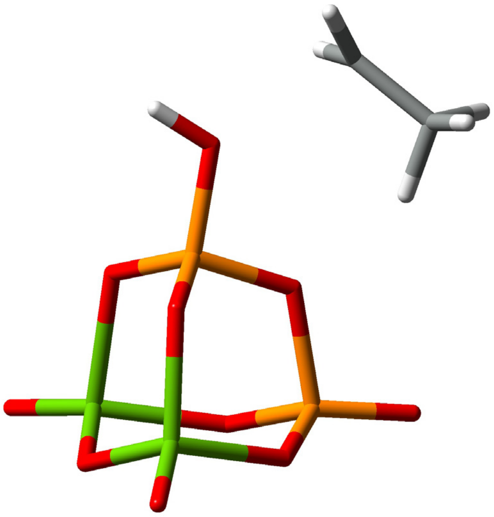

charge = 1, multiplicity = 2

V	-1.69512200	-1.53033400	0.07497900
V	-1.32581100	1.56337300	-0.52318900
P	0.88988800	-0.28965100	-0.98034000
P	0.22704100	0.28513900	1.80415300
O	-2.05748100	2.88436300	-0.93501500
O	1.02262300	0.52670900	3.01295200
O	2.30751300	-0.52838700	-1.74062600
O	-2.44834800	-2.88862500	-0.11882000
O	-0.02565200	-1.50826300	-1.08006100
O	1.40203200	-0.03799200	0.50353000
O	-0.73115600	-0.94080100	1.63671700
O	-2.32838100	0.18458000	-0.56342400
O	0.22460600	1.01877600	-1.51629800
O	-0.53171200	1.53854100	1.13074500
H	2.21914900	-0.78221000	-2.67447800
C	4.47379800	-0.57027100	-0.71312600
H	4.80571000	-0.27236900	-1.70279100
H	4.25785900	-1.62402600	-0.56685600
C	4.45676900	0.32265700	0.38408500
H	4.78656900	1.33772900	0.19058700
H	3.42223500	0.29833300	0.80696900
H	4.98026100	-0.15519400	1.23484300

TS12-15$_{V2P2}$:

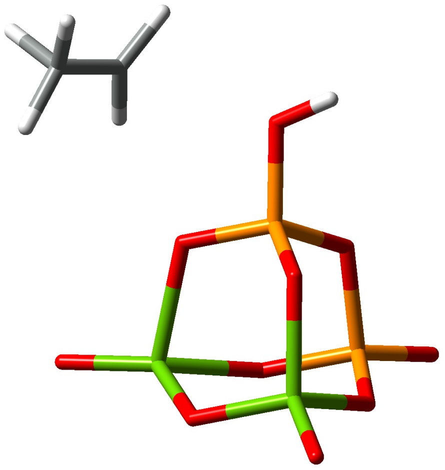

charge = 1, multiplicity = 2

V	-1.57528600	0.17378700	1.45586500
O	-2.19883400	-1.61680500	-2.47233400
O	1.06474800	2.94650800	-1.12154800
O	2.04789900	-2.06891800	0.48208500
O	-2.52392800	0.31057300	2.69396400
O	-0.05273800	-0.98088400	1.59210400
O	1.42852200	0.25977700	-0.12687500
O	-0.88221600	1.65572300	0.94201200
O	-2.28199800	-0.58235500	-0.04156800
O	-0.11674600	-1.68487000	-0.86829200
O	-0.86198500	0.58248200	-1.79254000
H	1.83628200	-2.98260900	0.73299100
C	4.19723000	-0.55096200	-0.27943500
H	3.57917900	-0.01654000	-0.99544400
H	4.48642400	-1.57061400	-0.51951900
C	4.74349900	0.11811300	0.82638400
H	4.27674200	1.05447700	1.12361300
H	5.19280600	-0.47892600	1.61665900
H	5.62074500	0.42590500	0.14625200
P	0.79213500	-1.08881600	0.28119400
V	0.15447300	1.74208000	-0.67530100
P	-1.52144400	-0.80914400	-1.45687900

TS15-16$_{V2P2}$:

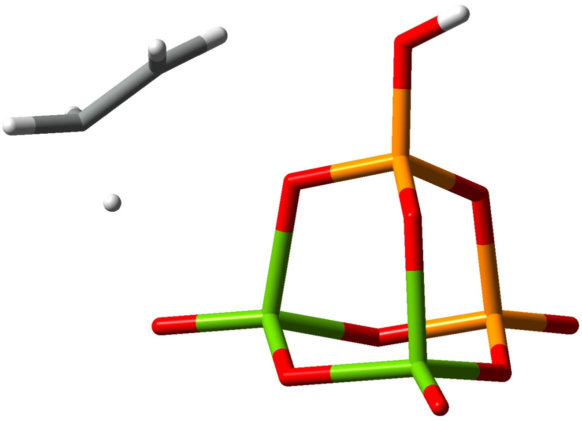

charge = 1, multiplicity = 2

V	1.92469300	-1.23585200	0.40590100
P	-0.19379100	0.79333000	1.37853500
O	1.66501900	2.68756800	-1.76935400
O	-2.17952000	-1.33845100	-1.71995700
O	-0.91874300	1.52311000	2.57487900
O	2.97284800	-2.17664500	1.08482000
O	0.82929000	-0.26631900	1.78713600
O	-1.40625300	0.11884000	0.56493400
O	0.29734000	-1.86521700	-0.51711300
O	2.23506800	0.35513800	-0.60271300
O	0.44069700	1.88150200	0.41197900
O	-0.00738400	0.64514800	-1.76541400
H	-0.35029000	1.93699900	3.24411700
C	-3.87603500	0.19878800	0.92518300
H	-3.68062100	1.26505000	0.88737400
H	-3.59394400	-0.31371900	1.83850300
C	-4.50451700	-0.47455100	-0.13338700
H	-5.05265500	0.15303600	-0.83631500
H	-3.58965100	-0.81572600	-0.76004800
H	-4.97555400	-1.42402800	0.12273500
V	-0.86246200	-0.74881300	-1.02186700
P	1.23831300	1.47219400	-1.07676900

TS12-17$_{V2P2}$:

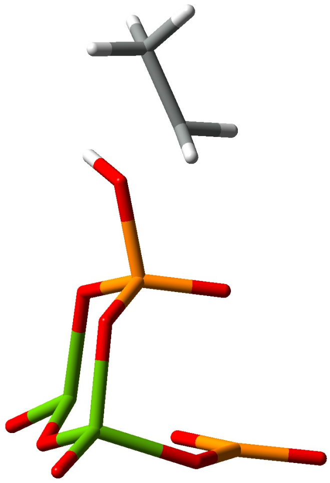

charge = 1, multiplicity = 2

P	-1.59619500	-1.12952900	-1.52335600
P	1.29617100	0.11622800	-0.15790100
V	-0.90656200	-0.91641300	1.50788500
V	-1.23780400	1.80043700	-0.14432800
O	-1.70749700	-1.94519800	-2.72906400
O	3.00268300	0.46628100	0.10408800
O	1.15079700	-0.70075300	-1.39355300
O	-1.56093700	0.63656400	1.36445900
O	-1.29074200	-1.59150500	2.86228300
O	-2.03635200	3.13090400	0.00349300
O	0.72472800	1.56246200	-0.13040400
O	0.92137700	-0.63711400	1.18171900
O	-1.74427900	0.39395800	-1.45145000
O	-1.47365600	-1.75583500	-0.10307800
C	4.13071600	-0.46333000	-0.41218000
H	3.90919800	-1.39853500	0.09346100
C	5.44526300	0.14513600	-0.02565600
H	5.54705700	0.24976400	1.05427900
H	6.23031700	-0.53199000	-0.37194600
H	3.21649400	1.41537100	0.03686200
H	3.95718500	-0.54334900	-1.48173600
H	5.61568800	1.10672800	-0.51481800

TS17-18$_{V2P2}$:

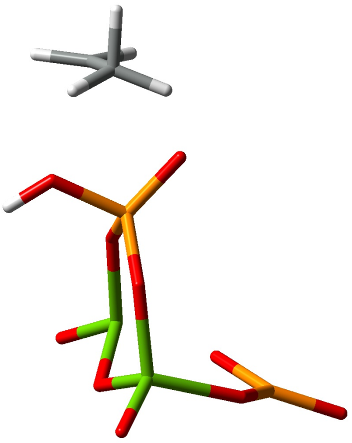

charge = 1, multiplicity = 2

P	2.71446400	-0.61532600	1.27962800
P	-1.74269700	0.20355700	0.25475300
V	0.71219300	-1.26886000	-1.06277700
V	1.07245100	1.75691500	-0.26489200
O	3.69294300	-1.05571500	2.27358000
O	-3.00281100	0.56854100	-0.73923800
O	-2.37331400	-0.12826600	1.56460700
O	1.19853700	0.28563800	-1.51751400
O	0.82468100	-2.26337000	-2.26636200
O	1.50781700	3.08174000	-0.97012800
O	-0.74376800	1.41868500	0.18668600
O	-0.99648400	-1.03394400	-0.45485800
O	2.31277400	0.83365000	1.01273600
O	1.92944500	-1.61192400	0.39471200
C	-4.91433000	0.31553800	0.81185500
H	-4.40942600	0.67390100	1.70228600
C	-5.31602500	-1.04637800	0.71772400
H	-4.72141900	-1.73554200	1.31385500
H	-6.29666700	-0.94046300	1.26103100
H	-2.79450500	0.92957700	-1.61366900
H	-5.24887600	1.04776700	0.08598500
H	-5.56887000	-1.39920700	-0.28005800

TS18-19$_{V2P2}$:

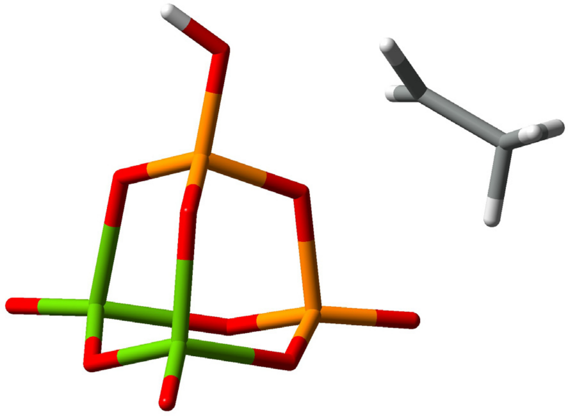

charge = 1, multiplicity = 2

V	1.70785500	-1.43708600	-0.39168100
V	1.26555200	1.66598100	0.09630800
P	-0.46321500	-0.28516000	1.48079700
P	-0.86487200	0.06980500	-1.41756100
O	1.96246500	3.06476600	0.14368900
O	-2.06714100	0.12867200	-2.27270500
O	-1.44988700	-0.53817800	2.69368500
O	2.59270300	-2.72576500	-0.38761100
O	0.54956200	-1.40756100	1.24959600
O	-1.51137900	-0.20475300	0.23736800
O	0.17653500	-1.07616100	-1.53589100
O	2.34780000	0.37332200	-0.15651900
O	0.22390900	1.12010300	1.58760100
O	-0.06901900	1.42903200	-1.16625300
H	-1.04246200	-0.72841600	3.55376300
C	-3.73318300	-0.60928500	0.43365000
H	-3.58906100	-0.39552200	1.48645800
H	-3.50654200	-1.61969100	0.11123300
C	-4.39046600	0.28094700	-0.45400300
H	-4.70625700	1.23095600	-0.03638200
H	-3.71493800	0.40905100	-1.34106200
H	-5.21029700	-0.26840900	-0.95053300

Structures of $[V_3PO_{10}]^{\bullet+}$

1_{V3P}:

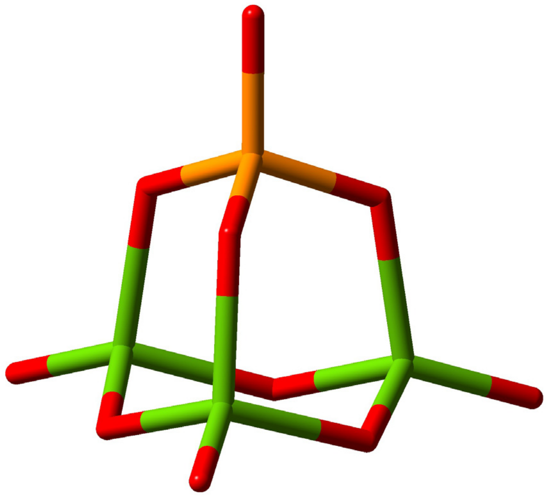

charge = 1, multiplicity = 2

P	-0.14464300	1.84477000	0.00000000
V	1.82230100	-0.39583400	0.00000000
V	-0.85547400	-0.61184000	1.54183000
V	-0.85547400	-0.61184000	-1.54183000
O	-0.16240100	3.44327900	0.00000000
O	3.32931800	-0.77533800	0.00000000
O	-1.56667800	-1.18638200	0.00000000
O	1.37416300	1.43835700	0.00000000
O	-1.55656600	-1.13653700	2.82562700
O	-1.55656600	-1.13653700	-2.82562700
O	-0.85547400	1.24550000	1.27402300
O	-0.85547400	1.24550000	-1.27402300
O	0.90037300	-0.97034200	-1.42355500
O	0.90037300	-0.97034200	1.42355500

2_{V3P}:

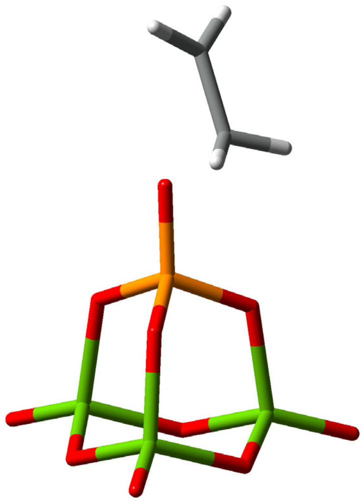

charge = 1, multiplicity = 2

V	0.82505600	-0.86663100	1.60297000
P	-1.39490100	0.31698300	-0.04962100
V	0.87781700	-1.06338900	-1.46468800
V	1.29199800	1.65877800	-0.09215200
O	1.11657300	-1.59452300	2.94944100
O	-2.89814200	0.64386400	-0.09497100
O	-0.95709200	-0.44432300	1.28303000
O	1.66819200	0.54680400	-1.44849700
O	1.20969100	-1.95784900	-2.69646200
O	1.98207800	3.05273400	-0.16921500
O	-0.56544200	1.66652100	-0.12322800
O	-0.91197100	-0.60659000	-1.25861100
O	1.62068600	0.72667200	1.40578800
O	1.23714500	-1.80291400	0.13029200
C	-5.32447300	0.40252400	-0.02695100
H	-5.78859400	0.65621300	-0.96931100
H	-5.74491100	0.79618000	0.88747400
C	-4.09981400	-0.38227900	0.00370000
H	-3.92886800	-0.90304500	0.94212300
H	-3.96417300	-1.03013400	-0.85799300

2_{V3P}-VO:

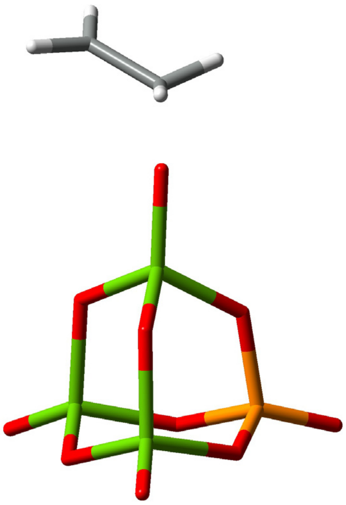

charge = 1, multiplicity = 2

P	1.21688300	-0.23923500	1.82991000
V	1.22907300	-1.33215400	-1.03744200
O	1.68103000	-0.43005400	3.20219900
O	-3.03956300	-0.23188600	0.29338600
O	-0.44305200	-0.31284000	1.62069700
O	1.61846400	0.32594000	-1.58717200
O	1.86535900	-2.47686300	-1.88036500
O	1.49334100	3.09740200	-0.99886200
O	-0.78909800	1.38991000	-0.47580900
O	-0.61794200	-1.31239600	-0.89881700
O	1.53176200	1.18791000	1.09938200
O	1.69365200	-1.32223200	0.69964800
C	-5.21285800	0.53894300	-0.46979000
H	-5.31363900	0.57060500	-1.54630000
H	-5.56742100	1.37925200	0.11136100
C	-4.52885100	-0.57298100	0.17894900
H	-4.81546600	-0.73234100	1.21649300
H	-4.54352700	-1.50264300	-0.38599100
V	-1.36985000	-0.13717400	0.13211600
V	1.03144800	1.67623100	-0.55960700

3_{V3P}:

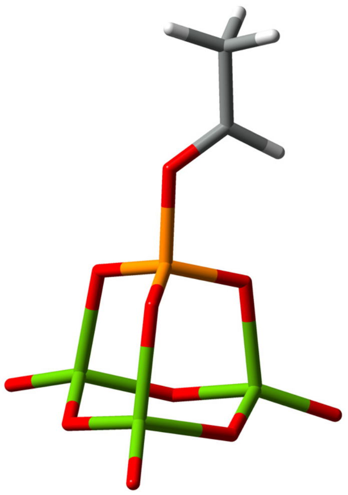

charge = 1, multiplicity = 2

V	-1.12616300	-1.56633300	-0.82230500
P	1.28201300	0.08655800	-0.38101100
V	-0.59141400	-0.13404500	1.87929900
V	-1.30219100	1.64237000	-0.74577100
O	-1.71759600	-2.83019100	-1.53204200
O	2.98580900	0.12024100	-0.75970100
O	0.78943000	-1.19907400	-1.07891100
O	-1.27292800	1.24122300	1.12915500
O	-0.77555500	-0.14791100	3.43599200
O	-2.21541100	2.87327900	-1.04860100
O	0.73381800	1.39080300	-0.94952900
O	1.25167000	-0.02663000	1.16730500
O	-1.81800600	-0.08272200	-1.35980400
O	-1.17536700	-1.57982500	0.97127200
C	5.30180200	0.14341400	-0.31432300
H	5.77204400	1.00856000	0.17727900
H	5.43061600	0.18242100	-1.39267600
C	3.91288900	0.09828900	0.09691300
H	5.81617000	-0.72493500	0.12309100
H	3.63056600	0.04600800	1.15273200

3_{V3P}-VO:

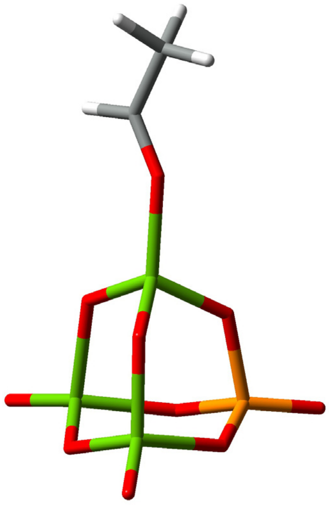

charge = 1, multiplicity = 2

V	1.37334300	-1.24742900	-1.10246000
P	1.07791100	-0.48271800	1.88060100
O	2.11514000	-2.27625000	-2.01694300
O	1.20972100	-0.93678800	3.26661900
O	-3.28751400	-0.22149600	-0.10921000
O	1.51204900	3.31237100	-0.64974500
O	-0.69343300	1.33728900	-0.44011900
O	-0.62600800	-0.49556600	1.40958000
O	1.45075700	0.97808800	1.43008200
O	1.80534800	0.37389300	-1.43236900
O	-0.54903600	-1.20696700	-1.12831500
O	1.60412400	-1.45414800	0.70007400
C	-4.34326000	0.42454800	-0.24674500
H	-4.27248800	1.48874000	-0.50514900
C	-5.66002300	-0.18068800	-0.07520900
H	-6.19779700	0.38345100	0.69991800
H	-6.24126700	-0.01259500	-0.99225400
H	-5.61098200	-1.23770600	0.17296400
V	-1.34629800	-0.13436100	-0.09141300
V	1.27053600	1.81102000	-0.27960600

4_{V3P}:

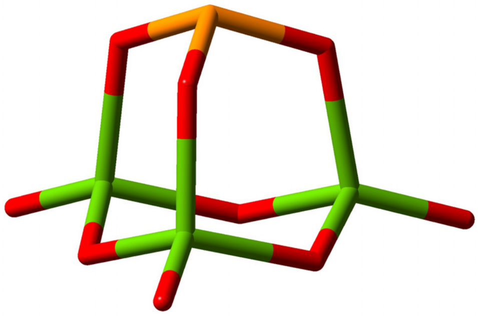

charge = 1, multiplicity = 2

V	0.71606600	1.63857700	-0.36543600
P	-0.00060400	-0.00007900	2.04131700
V	1.06171200	-1.43898000	-0.36561700
V	-1.77749900	-0.19955800	-0.36555500
O	0.59603000	1.36662900	1.49228100
O	1.63856200	0.18390200	-0.86435000
O	0.88449100	-1.20000200	1.49154900
O	-0.97799300	1.32599800	-0.86437100
O	-0.65912100	-1.50951900	-0.86391300
O	-1.48258600	-0.16626700	1.49291300
O	1.93765000	-2.62760500	-0.85156300
O	1.30695100	2.99130400	-0.85242000
O	-3.24365600	-0.36440300	-0.85485000

4_{V3P}-VO:

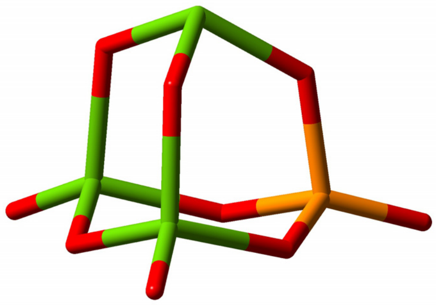

charge = 1, multiplicity = 2

V	1.53183500	-0.83272000	-0.42135700
P	0.00000200	1.81765700	-0.67055100
O	2.82016600	-1.52072800	-0.95322200
O	-0.00042900	3.18376500	-1.18396800
O	-2.82070000	-1.51976400	-0.95364500
O	-1.37310400	-0.61202200	1.42415000
O	0.00036000	1.68882600	1.01352400
O	-1.27195000	0.84924500	-1.00459300
O	0.00005500	-1.65467600	-0.85443600
O	1.37306400	-0.61274100	1.42351700
O	1.27230200	0.84994100	-1.00524800
V	0.00022600	0.25373800	2.00792000
V	-1.53198000	-0.83317600	-0.42092600

5_{V3P}:

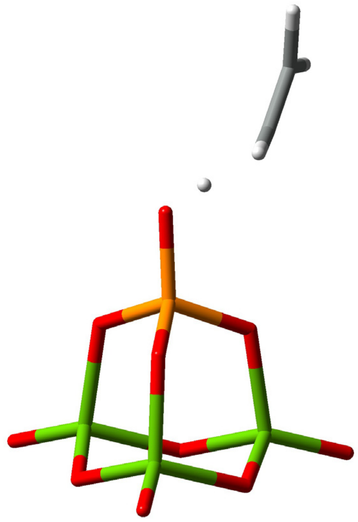

charge = 1, multiplicity = 2

P	1.23330000	-0.19446800	-0.62156400
V	-0.84725500	1.82296700	0.17381300
V	-1.64057700	-0.79415800	-1.24174300
O	1.04100000	-0.88568400	0.79953900
O	-0.95125400	0.89989200	1.70826400
O	0.84433800	1.33766300	-0.43094900
O	-1.68706400	-1.53121700	0.39363100
O	-1.90858200	0.96203100	-0.98658100
O	0.18125800	-0.83828800	-1.61369700
O	2.64228800	-0.39709300	-1.21140700
O	-1.10751800	3.35308500	0.30622300
O	-0.66814600	-1.58261900	3.03692800
O	-2.57304400	-1.46089800	-2.29545900
C	4.99795800	0.51422300	-0.03989100
H	3.49219900	-0.04498900	-0.74910900
H	4.98990800	1.59606500	-0.02075400
C	5.86914700	-0.45336200	-0.10064200
H	6.90974000	-0.24897000	-0.36785300
H	5.62301100	-1.49819100	0.07100000
V	-0.60882800	-0.85954900	1.65842700

6_{V3P}:

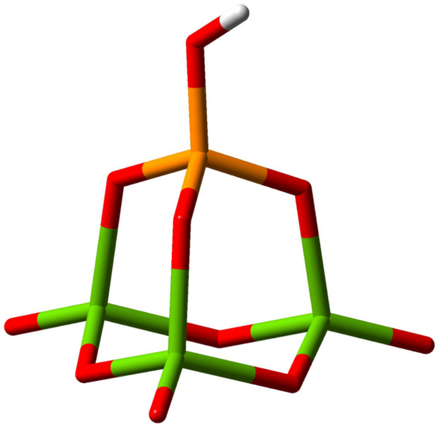

charge = 1, multiplicity = 1

V	0.87653300	1.55685800	-0.54471200
P	-0.04326100	-0.00544500	1.83888100
V	0.92828100	-1.52372100	-0.55267300
V	-1.76970200	-0.02788800	-0.59859000
O	0.70188800	1.28351900	1.29689800
O	1.67120100	0.03075300	-1.04922100
O	0.74520600	-1.26572100	1.29071100
O	-0.82626300	1.41763300	-1.08777400
O	-0.77904400	-1.43936900	-1.09400300
O	-1.50440500	-0.02792000	1.25384000
O	-0.17675700	-0.01151500	3.39282100
O	1.70506500	-2.79671200	-0.99624700
O	1.61014200	2.85760600	-0.98126500
O	-3.24496400	-0.05126900	-1.09041100
H	0.62479400	-0.01508500	3.94144500

7$_{V3P}$:

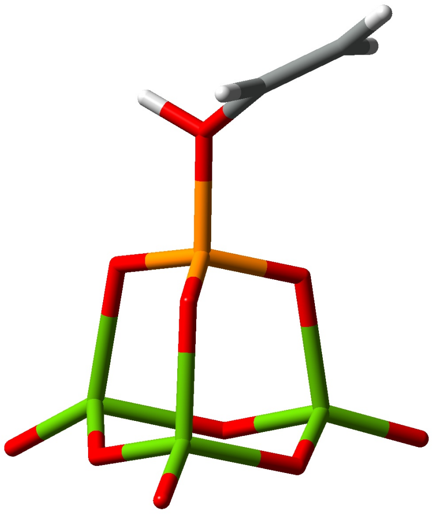

charge = 1, multiplicity = 2

V	0.71273100	-0.59232600	1.77510600
P	-1.23005000	-0.45785300	-0.42348100
V	0.49883800	1.90347400	-0.05199800
V	1.64637600	-0.82019400	-1.33373600
O	1.05982900	-1.15453600	3.19506100
O	-2.86394300	-0.90559600	-0.88012700
O	-1.07407800	-0.97570500	1.02303100
O	1.53252500	1.06958300	-1.15021300
O	0.59754800	3.46280500	-0.15245600
O	2.86541700	-1.43655600	-2.09081900
O	-0.39903800	-1.17427600	-1.48610900
O	-1.23544400	1.08357200	-0.50138100
O	1.69225000	-1.24161400	0.53179800
O	0.73970900	1.20318900	1.57969000
H	-2.91818700	-1.65146800	-1.50965400
C	-4.06138700	-0.70876200	-0.01367200
H	-4.11856200	-1.47767900	0.73980000
C	-4.81608400	0.33055200	-0.26539700
H	-5.68895900	0.49406000	0.35514900
H	-4.60963900	1.03326700	-1.06204100

8$_{V3P}$:

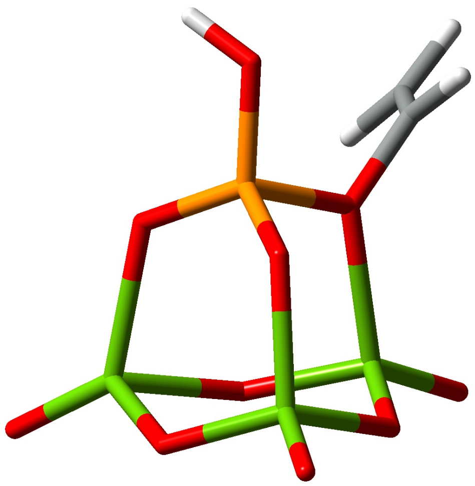

charge = 1, multiplicity = 2

V	-0.70267700	0.26400500	1.58148400
P	-0.75960300	-0.60974100	-1.47183200
V	1.61440900	-1.34223800	0.32945600
V	1.20916200	1.67745800	-0.63267900
O	-1.51773300	0.61017200	2.87482200
O	-1.73979300	-1.20280100	-2.54409500
O	-1.73702500	-0.39448800	-0.08774700
O	2.26955700	0.16256500	-0.19166400
O	2.69591400	-2.45108400	0.53838000
O	1.82891700	3.10074500	-0.79863300
O	-0.17363900	0.75841200	-1.81328400
O	0.25913700	-1.67257000	-1.01760900
O	0.03134700	1.62967100	0.88089600
O	0.52420800	-1.02040700	1.73508400
C	-3.18689400	-0.55523600	-0.12785900
H	-3.46550600	-1.58954100	-0.25783200
C	-3.96310600	0.49090200	0.03431800
H	-3.58839200	1.49839600	0.16383600
H	-5.03514800	0.34224900	0.05310400
H	-1.92458400	-0.66287400	-3.32958

9_{V3P}:

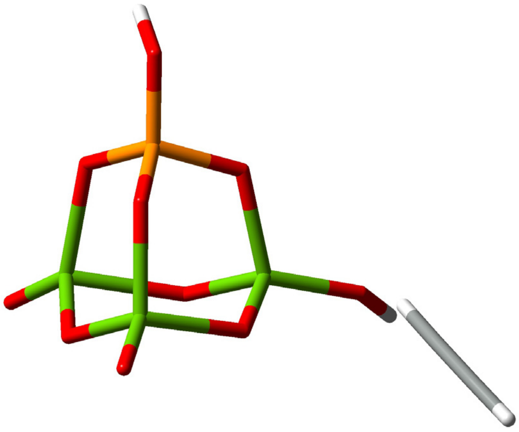

charge = 1, multiplicity = 2

P	-1.28657300	-0.22679000	1.67170800
V	-1.39890800	-1.16430600	-1.17678300
V	-0.85343300	1.90544300	-0.51330000
O	2.95153200	-0.83068800	0.34206600
O	-1.92013300	-0.55219700	3.06940500
O	0.27571800	-0.59155300	1.68994000
O	-1.52100400	0.49011800	-1.61150300
O	-2.15056500	-2.11451400	-2.16182900
O	-0.73916300	3.38456100	-1.00055300
O	-1.43910900	1.26088100	1.25374500
O	-1.94910200	-1.19393700	0.62391200
O	0.95112400	1.09678800	-0.30329900
O	0.41797500	-1.47082500	-0.90861100
C	5.81029000	-0.43222600	-0.43395200
H	6.01446200	-1.34229200	-0.95098700
C	5.62291300	0.60294800	0.14669300
H	3.87369400	-0.46076900	0.18671200
H	5.51619400	1.53332900	0.65594500
H	-1.90305400	0.14622400	3.74187500
V	1.30364000	-0.45105200	0.17130900

10_{V3P}:

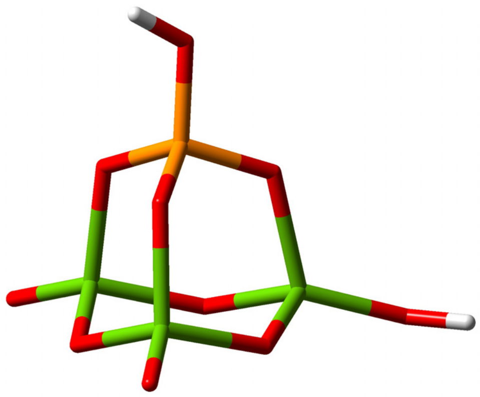

charge = 1, multiplicity = 2

V	0.97174100	-1.45270900	-0.70289400
V	-1.88174500	-0.25561400	-0.19863300
V	0.63153200	1.59925800	-0.75319800
P	0.41501800	0.08385700	1.79410400
O	-3.59348200	-0.30900400	-0.37699500
O	0.63173600	0.12306400	3.34340900
O	1.05331700	2.96058000	-1.38564700
O	1.75412100	-2.67286300	-1.27337800
O	1.44627500	0.10151200	-1.39854000
O	0.94504400	1.39589700	1.08872700
O	1.17989700	-1.13755200	1.12055700
O	-0.84282000	-1.53254100	-0.82702500
O	-1.08193100	1.25398300	-0.86630800
O	-1.11316100	-0.09774600	1.54033000
H	1.52177500	0.31433000	3.67994100
H	-4.37415700	0.25367400	-0.25387300

11_{V3P}:

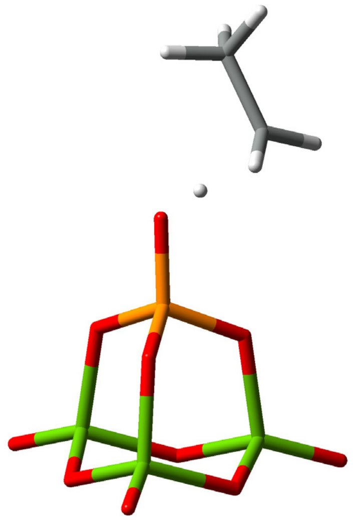

charge = 1, multiplicity = 2

V	-1.60881200	1.53823300	-0.22136300
V	-0.86761200	-1.21611200	-1.38164800
P	1.20656400	0.51370900	-0.06334700
O	-1.08272100	-2.23095700	-2.54371000
O	2.66227700	1.01809700	-0.12850600
O	-1.10838100	-1.44044300	3.05843900
O	-2.45327900	2.83305700	-0.40701200
O	-1.84903200	0.68721600	1.34033900
O	0.84684400	-0.18508400	1.32081600
O	0.23954700	1.75407000	-0.24209700
O	-1.83601200	0.28765400	-1.48991500
O	-1.16034300	-1.87064000	0.26291300
O	0.85835100	-0.54221900	-1.20242100
C	4.99666400	-0.54095500	-0.00754300
H	4.76914400	-1.05890000	0.92028600
H	3.46984300	0.38122000	-0.07330300
C	6.02685700	0.52377900	-0.00747400
H	5.96930500	1.16713000	0.87200900
H	7.02424000	0.05426700	0.01961000
H	6.00276100	1.13458500	-0.91142600
H	4.80649500	-1.09527400	-0.92270400
V	-0.88094100	-0.78610500	1.66323100

12_{V3P}:

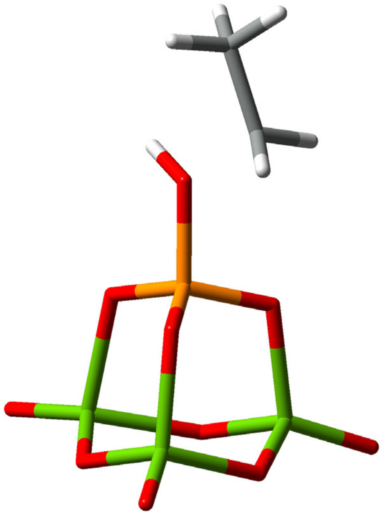

charge = 1, multiplicity = 2

V	-1.49720000	1.72400600	-0.05049900
V	-0.92266300	-0.98822500	1.57313200
P	1.21105100	0.31154800	0.04780500
O	-1.32451700	-1.79011400	2.85680100
O	-1.15618100	-1.86492300	-2.84913400
O	2.89889100	0.68122700	0.10408000
O	-1.97012400	3.21490000	-0.08661900
O	0.56510300	1.68944400	0.00317900
O	1.03523800	-0.55301500	-1.22929800
O	-1.58754200	0.51361400	-1.53962500
O	-1.67296900	0.55319500	1.46168500
O	0.96424500	-0.52152800	1.32878700
O	-1.21672700	-1.82375100	0.00570800
H	3.11254900	1.59828000	-0.15474400
C	4.05554200	-0.35037300	-0.12970100
H	3.90463900	-0.68832000	-1.15116100
H	3.81378800	-1.12286700	0.59447900
C	5.35253800	0.35254600	0.12332100
H	5.53195100	1.16138000	-0.58794000
H	5.42830300	0.72105600	1.14595200
H	6.14720800	-0.38417900	-0.02553700
V	-0.83387800	-1.02986800	-1.56369100

13_{V3P}:

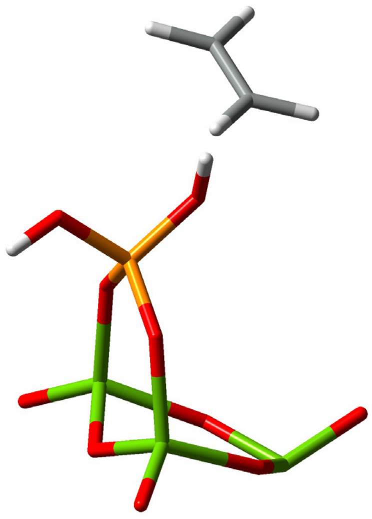

charge = 1, multiplicity = 2

V	-1.23745800	1.61451200	-0.74339700
V	-0.63899300	-0.34741600	1.68761600
P	1.78545700	0.62347100	-0.04932200
V	-2.45679600	-1.51859800	-0.75354800
O	-0.83141000	-0.45913900	3.23755300
O	-1.96235800	-2.58600600	-1.78941200
O	2.90484200	1.59076800	0.53216400
O	-1.87491000	3.03272900	-0.89119700
O	0.68020100	1.36916500	-0.83434600
O	2.53256200	-0.32549000	-1.04132700
O	-2.19878000	0.03159200	-1.37801400
O	-1.31722600	1.09943200	1.11286900
O	1.14467900	-0.18823700	1.14584300
O	-1.55078800	-1.65073000	0.71616000
H	2.65638200	2.16747500	1.26948100
C	5.59427000	-0.83200500	-1.12028500
H	5.97192200	0.14665300	-0.84637100
H	5.58213700	-1.06821400	-2.17841000
C	5.21272800	-1.71819200	-0.20102900
H	5.27233200	-1.49919100	0.85941900
H	3.46328500	-0.61205500	-0.83067700
H	4.88030100	-2.71368500	-0.47347700

14_{V3P}:

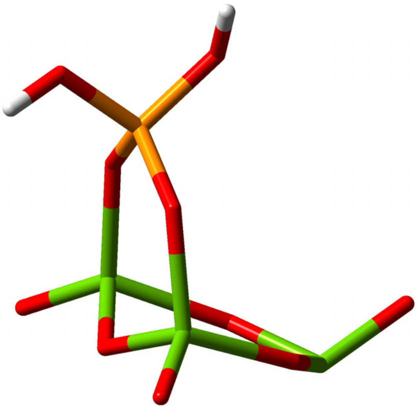

charge = 1, multiplicity = 2

V	0.11117700	1.74268600	-0.59920700
V	0.02439200	-1.42614300	-0.92479700
P	-2.22075400	-0.05620600	0.78239300
V	2.57402100	-0.16493400	0.67527600
O	-0.03943000	-2.52866400	-2.03328800
O	2.76542900	-0.29414900	2.22456700
O	-3.75032600	-0.04407300	0.36383900
O	0.06984700	2.95307400	-1.58356200
O	-1.45721800	1.23648500	0.43222000
O	-2.24829800	-0.16225700	2.35707300
O	1.76560000	1.28140500	0.33337300
O	0.06546300	0.11640900	-1.63218100
O	-1.51750000	-1.31348100	0.14613000
O	1.58151700	-1.46102400	0.09343600
H	-3.96806600	-0.17111300	-0.57163200
H	-2.92184300	-0.74260700	2.74362800

15_{V3P}:

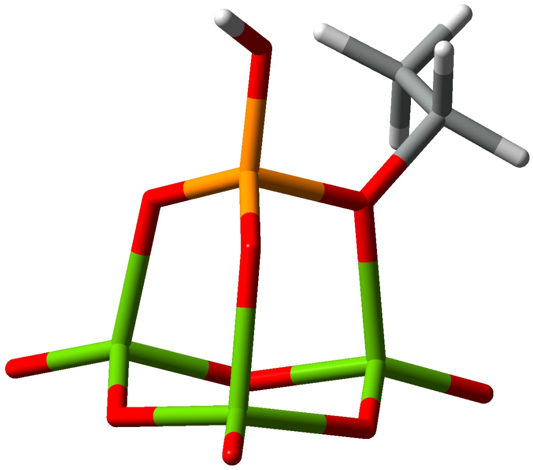

charge = 1, multiplicity = 2

V	-0.86498300	1.84224000	-0.11858400
V	-2.00582500	-1.12222700	0.13907800
O	-3.11330400	-2.12160700	0.60298200
O	1.47620600	-0.92951700	-2.80318200
O	1.67009600	-0.36955700	2.73373500
O	-1.27891800	3.34732200	-0.21665000
O	0.33020700	1.33439500	1.30410200
O	1.62946600	-0.59020800	0.21544600
O	0.10222200	1.19206700	-1.51524700
O	-2.19990200	0.78764000	0.09257500
O	-0.53305700	-1.08689000	1.52764500
O	-0.72497600	-1.50410200	-1.26620000
H	1.28712800	-0.53235400	3.61093600
C	3.16474000	-0.81653500	0.27913400
H	3.32176500	-1.52003400	-0.53382300
H	3.32599500	-1.30422700	1.23505400
C	3.90068200	0.48016400	0.11588600
H	3.68884500	0.95669600	-0.84179000
H	3.69698400	1.17729000	0.92869100
H	4.96968100	0.24982800	0.13836600
P	0.67551200	-0.16209800	1.53148200
V	0.62385400	-0.50496200	-1.55447000

16_{V3P}:

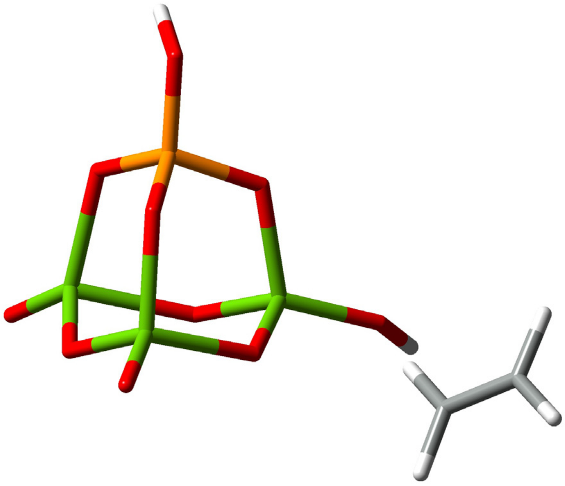

charge = 1, multiplicity = 2

V	-0.94288000	1.96268100	-0.10454000
V	-1.48830900	-0.91221600	-1.36720900
P	-1.30754300	-0.57178400	1.61044600
V	1.24284200	-0.45937500	0.03534600
O	-2.25982800	-1.65109500	-2.50638900
O	2.89561200	-0.84252300	0.08060100
O	-1.90498900	-1.17986100	2.92788300
O	-0.85130800	3.51083700	-0.28817000
O	-1.47738900	0.96828200	1.51046200
O	0.25518100	-0.91875900	1.51923200
O	0.86926300	1.15027600	-0.10565600
O	-1.63279500	0.79520200	-1.45093200
O	-1.99171800	-1.31138700	0.40349900
O	0.33541800	-1.24730300	-1.20974400
H	-1.89404700	-0.62225500	3.72108300
C	5.48948200	0.71566300	-0.33118900
H	5.33239500	1.53910900	0.35673100
H	5.37182400	0.93337000	-1.38698700
C	5.88658800	-0.48592500	0.09476400
H	6.05669800	-0.68978500	1.14596200
H	3.82651400	-0.45210800	0.01263000
H	6.09576900	-1.29443400	-0.59658700

TS2-3$_{V3P}$:

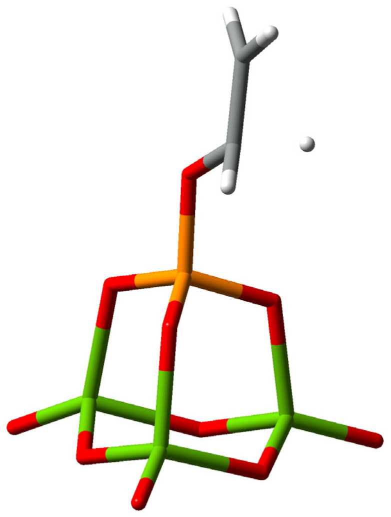

charge = 1, multiplicity = 2

V	-0.79513500	-1.15717300	-1.43993900
P	1.31135700	0.47492100	-0.09563200
V	-0.67642500	-0.89868600	1.63376200
V	-1.47817900	1.64096900	-0.13131700
O	-1.07710200	-2.08601900	-2.66957300
O	2.88773300	0.89328700	-0.19763100
O	1.01294200	-0.49559800	-1.28900700
O	-1.56204800	0.56742300	1.40591200
O	-0.92503200	-1.58090600	3.02285600
O	-2.49623700	2.82656700	-0.08590500
O	0.50867800	1.78431200	-0.17484400
O	1.12988800	-0.29805900	1.25518900
O	-1.70673600	0.32555700	-1.43970000
O	-0.97561000	-1.91647600	0.17602000
C	5.20818800	0.41396800	-0.12654600
H	6.02978900	-0.19680500	0.23447100
H	5.40904800	1.23222300	-0.81305800
C	3.88738300	0.01825500	0.15811100
H	4.08876600	-0.77457500	-0.68562700
H	3.70082300	-0.59623000	1.04512500

TS2-3$_{V3P}$-VO:

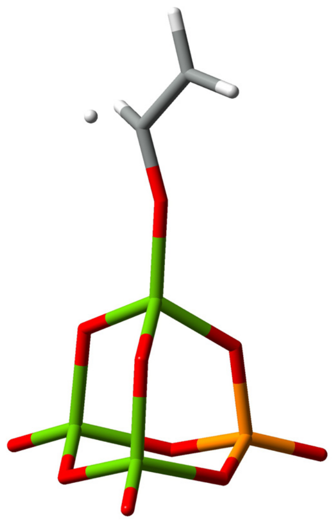

charge = 1, multiplicity = 2

P	1.08947000	-0.18948900	1.88658900
O	1.40457200	-0.32086800	3.31020700
O	-3.17521500	0.01038100	-0.03512800
O	-0.55026100	-0.17265100	1.54354900
O	1.80692600	0.14122000	-1.49002000
O	1.80053200	-2.68432400	-1.64961900
O	1.80390200	2.95061400	-1.09549800
O	-0.58922300	1.43984500	-0.63838300
O	-0.59770200	-1.31831400	-0.88539300
O	1.57463200	1.16412400	1.12323900
O	1.57360900	-1.37257100	0.87787100
C	-5.54377700	-0.35291200	0.13577100
H	-6.54682300	0.02765400	-0.02636500
H	-5.41641300	-1.36383100	0.51056900
C	-4.43378700	0.43226700	-0.30573200
H	-4.68035600	0.27478900	-1.41023700
H	-4.57679000	1.51933000	-0.19931400
V	-1.38236300	-0.00392000	-0.00451700
V	1.21997200	1.58797300	-0.60382800
V	1.22019800	-1.44454800	-0.89775600

TS2-5$_{V3P}$:

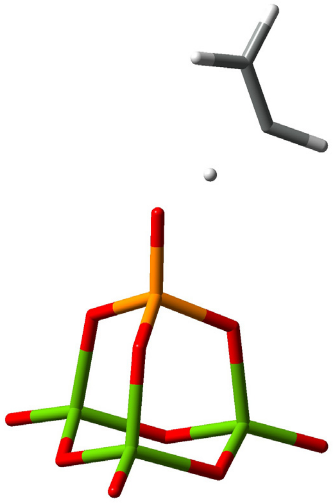

charge = 1, multiplicity = 2

V	0.60924500	1.83828000	0.27885600
P	-1.28882300	-0.42967300	-0.31577100
V	1.06131700	-0.93951000	1.51033000
V	1.48545000	-0.51099000	-1.49916000
O	-1.07462000	1.13538600	-0.05133500
O	1.14788900	0.84798600	1.67122400
O	-0.69005500	-1.17172000	0.96910300
O	1.54269000	1.24590500	-1.13108400
O	1.96822300	-1.34125000	0.01627900
O	-0.33661300	-0.81986200	-1.53493600
O	-2.73566400	-0.80274500	-0.60333500
O	1.48227800	-1.74036100	2.78027400
O	0.65619300	3.38053300	0.50893700
O	2.26965900	-0.94779000	-2.77362400
C	-4.94351400	0.35306100	0.06417100
H	-3.73607700	-0.21914800	-0.30645700
H	-4.84468000	1.41137400	0.28777000
C	-5.99314200	-0.42526200	-0.10935000
H	-6.92852000	-0.02014100	-0.50438500
H	-5.96658800	-1.50137900	0.04810200

TS7-8$_{V3P}$:

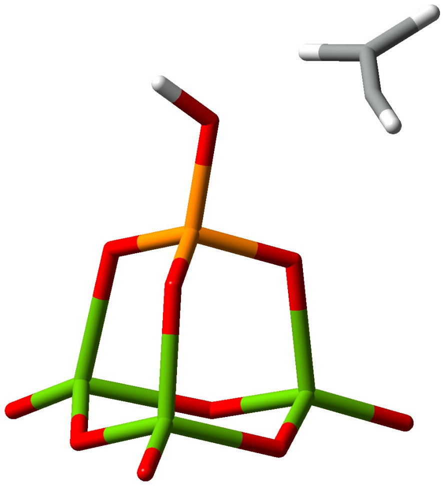

charge = 1, multiplicity = 2

V	-0.25159200	-0.07128400	1.76681900
P	-1.00635900	-0.18230300	-1.11219100
V	1.59336200	-1.43353400	-0.31082700
V	1.29494500	1.73350200	-0.42158700
O	-0.82054200	-0.04683500	3.23375900
O	-2.38463000	-0.40580500	-1.93332200
O	-1.58767700	-0.27170700	0.35704500
O	2.28712700	0.11958600	-0.60568000
O	2.56277000	-2.62792600	-0.61277700
O	1.92328500	3.16758700	-0.46466900
O	-0.37207500	1.18438800	-1.42100800
O	-0.02218700	-1.36606200	-1.37144300
O	0.49090400	1.38882500	1.29420600
O	0.86837100	-1.41519000	1.33432900
H	-2.33288000	-0.45758800	-2.90077700
C	-4.09612800	-0.56812600	-0.19174000
H	-3.73670000	-1.57463700	-0.08676600
C	-4.72506300	0.52428900	-0.19031400
H	-5.73456100	0.46704900	0.24664000
H	-4.38053500	1.48812900	-0.55871600

TS8-9$_{V3P}$:

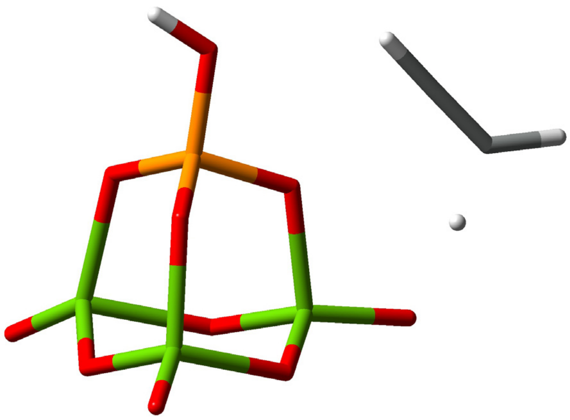

charge = 1, multiplicity = 2

V	0.94456900	0.27379900	-1.25586300
P	0.41461200	-0.32703300	1.61932600
V	-1.40740200	-1.53141100	-0.45892200
V	-1.58522400	1.58416600	0.22850400
O	2.29797000	0.55099900	-2.11162400
O	1.28462200	-0.63071400	2.90921800
O	1.53916100	-0.09904900	0.49693600
O	-2.32754100	-0.08982900	-0.30900700
O	-2.28028200	-2.78610600	-0.79440000
O	-2.41603000	2.90289900	0.11032500
O	-0.47121100	0.92927000	1.75562700
O	-0.43179600	-1.57335100	1.18513000
O	-0.07903000	1.59428000	-1.00555300
O	-0.02621700	-1.15949600	-1.60068400
C	4.03180900	-0.08790500	0.69694900
H	3.72000300	-0.33480000	1.69244600
C	4.41609100	0.19706800	-0.45119700
H	3.54464100	0.36814500	-1.23362000
H	5.41027100	0.31705800	-0.87470200
H	0.80665300	-0.72184300	3.74820200

TS12-13$_{V3P}$:

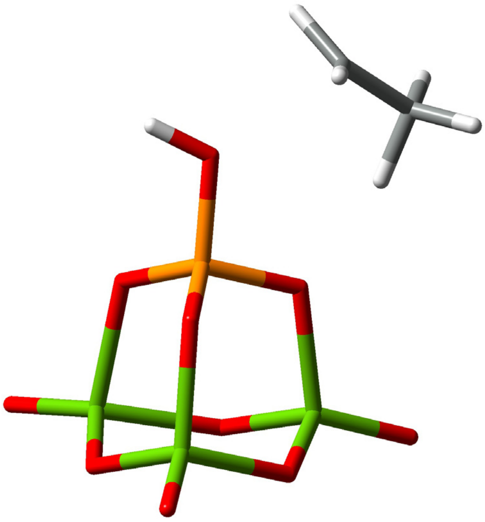

charge = 1, multiplicity = 2

V	-1.68613900	-1.55046500	-0.20804100
V	-1.29720200	1.58069200	-0.63392500
P	1.02281600	-0.24184600	-0.93526800
V	0.04093700	0.19819400	1.82898300
O	-1.98529200	2.88686700	-1.16236600
O	0.48265400	0.35452500	3.32950300
O	2.44186500	-0.46424000	-1.71662900
O	-2.37579600	-2.90590700	-0.58358700
O	0.12122700	-1.46281500	-1.12508900
O	1.49620900	-0.04074100	0.55792300
O	-0.94412900	-1.17356200	1.53038200
O	-2.25739100	0.16889000	-0.83529100
O	0.37432500	1.08318600	-1.45135200
O	-0.77463000	1.61652600	1.09560400
H	2.33937900	-0.80234900	-2.62145400
C	4.58650500	-0.07792600	-0.93718600
H	4.50260200	0.85076300	-1.49139100
H	4.82294600	-0.96813200	-1.50983500
C	4.54244400	-0.09089100	0.47980700
H	4.88971400	0.84868600	0.92250300
H	3.43663900	-0.05436400	0.70966300
H	4.93573100	-0.98952400	0.94963400

TS12-15$_{V3P}$:

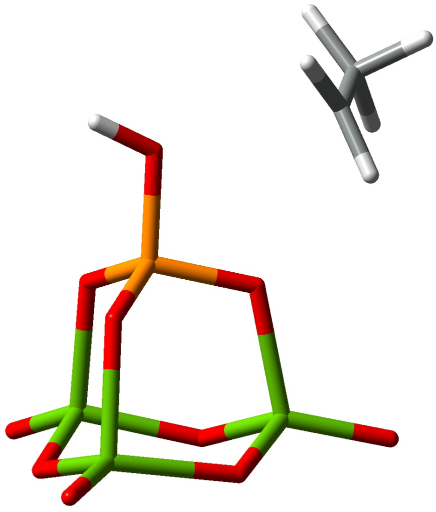

charge = 1, multiplicity = 2

V	1.24590700	1.69651800	-0.36895500
V	1.82839800	-1.42699800	-0.20255000
O	2.66208400	-2.69022900	-0.60979400
O	-0.92303600	-0.06256100	3.18227400
O	-2.18354700	-0.46552000	-2.05822600
O	1.88003000	3.09655000	-0.68339300
O	-0.26754300	1.18000600	-1.42544500
O	-1.52413500	-0.23005800	0.31082500
O	0.51985400	1.51884800	1.28032000
O	2.33744400	0.38347000	-0.57535000
O	0.14783900	-1.36146500	-1.31746800
O	0.86249600	-1.28657100	1.46129500
H	-2.00014100	-0.61465100	-2.99909500
C	-4.21096400	-0.77097700	-0.29219400
H	-3.65870700	-1.19800700	0.53911300
H	-4.38402500	-1.40441800	-1.15695500
C	-4.83304000	0.48675400	-0.17622300
H	-4.45364400	1.15391900	0.59488000
H	-5.17935700	0.95862600	-1.09327700
H	-5.76312500	0.00377100	0.28096200
P	-0.87728900	-0.23552400	-1.13846100
V	-0.25819700	-0.02259800	1.75417700

TS15-16$_{V3P}$:

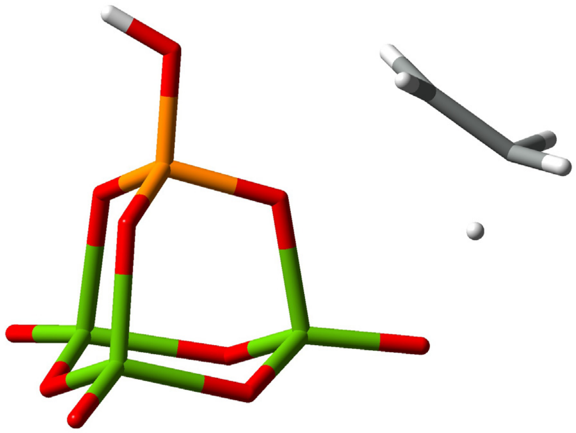

charge = 1, multiplicity = 2

V	1.66085100	-1.56152000	-0.24603300
V	1.43786100	1.61339500	-0.12979300
P	-0.32934300	-0.04352700	1.62549600
O	2.25820900	2.94394700	-0.22184000
O	-2.26589100	-0.06960300	-2.17862000
O	-1.14770100	-0.08122200	2.98174900
O	2.49259600	-2.87742100	-0.09252100
O	0.58229000	-1.26024500	1.41972100
O	-1.49972500	-0.03005600	0.51458800
O	0.08710800	-1.39173100	-1.38773800
O	2.40794200	0.20530600	-0.28463600
O	0.45486800	1.30886800	1.47176000
O	0.07042400	1.42844100	-1.33136100
H	-0.63008700	-0.19549200	3.79398000
C	-3.92455700	-0.05132000	0.85220500
H	-3.70852900	0.87791600	1.36698900
H	-3.69023000	-0.96507400	1.38590700
C	-4.54105300	-0.07002800	-0.40699100
H	-5.05863900	0.84516400	-0.69462300
H	-3.62163500	-0.06737300	-1.12598300
H	-5.03986300	-1.00074200	-0.67713600
V	-0.92645800	-0.03116600	-1.28642100

Structures of $[V_4O_{10}]^{\bullet+}$

1_{V4}:

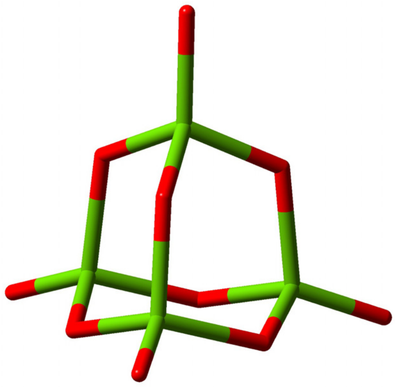

charge = 1, multiplicity = 2

V	-1.90148000	0.01631300	-0.15038200
V	0.73679700	-1.44959900	-1.00656800
V	0.51782300	-0.20212700	1.81947000
V	0.73436800	1.63678700	-0.66642200
O	-3.64717300	0.02776300	-0.27583200
O	-1.13617100	-1.26245200	-0.99861800
O	-1.13926800	1.45064600	-0.70058700
O	-1.29494500	-0.16318700	1.47057100
O	1.32254500	-2.63359500	-1.82977600
O	0.91148600	-0.36625500	3.31589700
O	1.32184800	2.97204500	-1.20915400
O	1.07576300	1.33545600	1.05970400
O	1.07533500	-1.53899900	0.74341300
O	1.25899500	0.17462700	-1.56440000

2_{V4}:

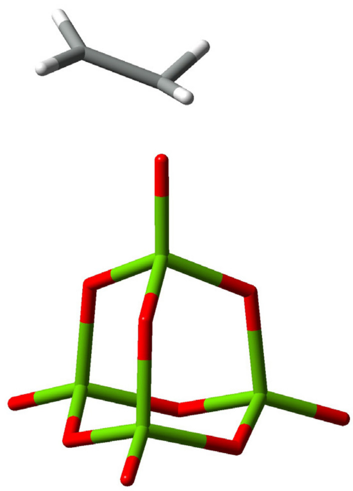

charge = 1, multiplicity = 2

V	1.37479900	1.53937100	-0.63557500
V	-1.42404900	0.24353100	-0.14669800
V	0.94883400	-0.30108000	1.81168300
V	1.00463800	-1.50041300	-1.03563300
O	2.10057400	2.81748600	-1.15921300
O	-3.09112800	0.45030200	-0.27433900
O	-0.47157000	1.60046100	-0.68969300
O	1.35138800	-1.66543000	0.72334400
O	1.32519700	-0.54504400	3.30642900
O	1.42173800	-2.73684400	-1.89119000
O	-0.79711900	-1.11512500	-1.05343000
O	-0.85654400	-0.03591200	1.47218300
O	1.75378200	0.04807200	-1.55821500
O	1.69479500	1.16276900	1.09308500
C	-5.24949500	-0.61800400	-0.00733200
H	-5.31366700	-1.23721600	0.87714600
H	-5.63885500	-1.00052100	-0.94077600
C	-4.55995500	0.66954900	0.03623400
H	-4.87450200	1.36909900	-0.73576800
H	-4.56229900	1.14109700	1.01742300

3_{V4}:

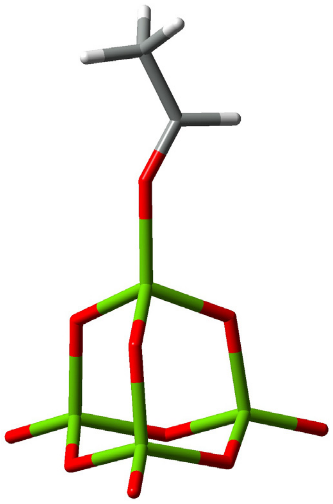

charge = 1, multiplicity = 2

V	0.99671200	1.82777900	-0.11432500
V	1.18605000	-0.92260400	-1.51051100
V	-1.41425300	-0.16672500	0.06329400
V	1.27378900	-0.75133300	1.56505300
O	1.69642200	-1.67867800	-2.78042700
O	1.85962200	-1.36812200	2.87692300
O	-3.35439500	-0.21950600	0.05320500
O	1.36238600	3.34534400	-0.21173300
O	-0.78948800	1.47880500	-0.03938300
O	-0.57148700	-0.86312800	1.42571900
O	1.64507000	0.98563000	1.34396300
O	1.56598400	0.82964800	-1.50145200
O	-0.63434100	-1.01821700	-1.26119400
O	1.80473700	-1.55931000	0.05578800
C	-4.43580100	0.39105600	0.02562900
H	-4.40974700	1.48812000	0.01333500
C	-5.73015100	-0.28964900	0.00854300
H	-6.31946000	0.06302500	0.86541800
H	-6.28792800	0.04942000	-0.87456200
H	-5.63607300	-1.37243000	0.01876200

4_{V4}:

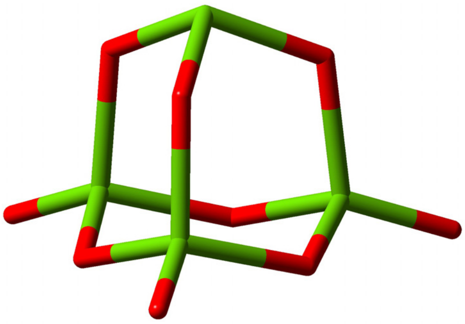

charge = 1, multiplicity = 2

V	0.00080800	-0.00205100	2.05862100
V	-1.78270700	-0.15472100	-0.47931900
V	1.02542100	-1.46524700	-0.48140300
V	0.75636000	1.62165100	-0.47919400
O	-1.58293000	-0.13962200	1.37329500
O	0.67296100	1.43865600	1.37277000
O	0.91190400	-1.30363900	1.37061100
O	-3.24284300	-0.28216400	-1.00682500
O	1.86594400	-2.66587000	-1.00881900
O	1.37674100	2.95009100	-1.00501900
O	1.65854000	0.14532200	-0.95827500
O	-0.70436400	-1.50700600	-0.95949800
O	-0.95561200	1.36528800	-0.95701700

5_{V4}:

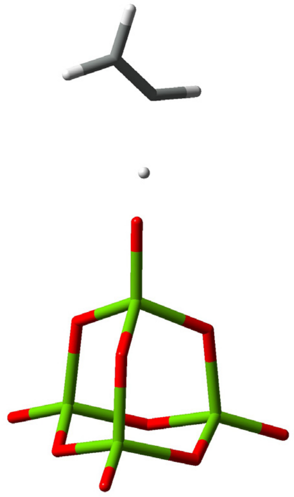

charge = 1, multiplicity = 2

V	1.34205100	-0.13098400	-0.22734900
V	-1.16045900	1.71854800	-0.52611700
V	-1.42273100	-1.31258300	-1.06675400
O	0.78759200	-0.36343700	1.40591400
O	-1.47833000	1.32911400	1.19910600
O	0.66126900	1.40826300	-0.66562700
O	-1.72799000	-1.49226900	0.69436500
O	-1.86169200	0.37964500	-1.49079500
O	0.42114400	-1.28533300	-1.15470700
O	3.00859600	-0.26153100	-0.43061800
O	-1.63644300	3.13940900	-0.96069800
O	-1.37008800	-0.52419900	3.32067400
O	-2.11392000	-2.39645400	-1.95042000
C	5.52688500	0.56932100	0.04028500
H	3.99133900	0.07134800	-0.26538700
H	5.51362600	1.64935300	-0.01881800
C	6.36560900	-0.42450700	0.04600600
H	7.39880500	-0.27368500	-0.28099900
H	6.10024800	-1.44017800	0.32817000
V	-1.01451700	-0.28982400	1.81942600

6_{V4}:

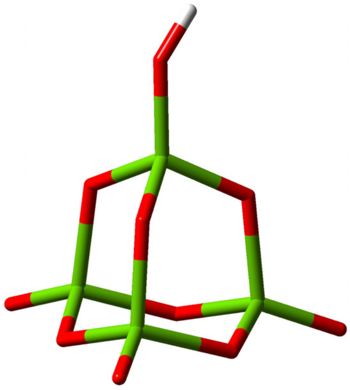

charge = 1, multiplicity = 1

V	-0.64088100	-0.39676200	1.75631600
V	1.88921300	0.07202600	-0.03725200
V	-0.76909400	1.67446900	-0.53979400
V	-0.65600000	-1.35081300	-1.18190500
O	1.19454300	-0.27815400	1.51221900
O	-1.19704600	1.19792500	1.12985900
O	1.10409800	1.52400500	-0.49527900
O	-1.10494200	-1.62497200	0.53425100
O	-1.21045100	0.31007900	-1.60752600
O	1.18192900	-1.12463300	-1.07309400
O	3.60372100	0.05296500	-0.04511300
O	-1.35627500	3.04738700	-0.98286900
O	-1.11258100	-0.72099700	3.20476400
O	-1.14045600	-2.46232100	-2.15914200
H	4.36520200	0.65453900	-0.08396700

7_{V4}:

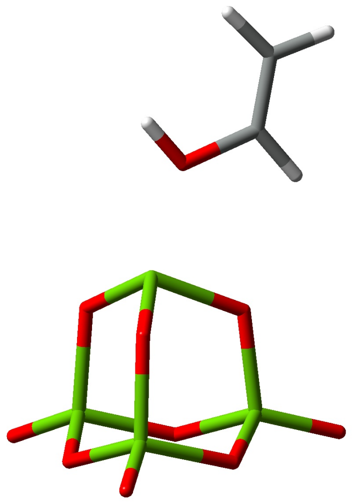

charge = 1, multiplicity = 2

V	-1.25963700	0.00151600	-0.79323200
V	1.42846000	-1.54862300	-0.59081900
V	1.42145100	1.56073500	-0.56982900
O	0.22127800	-0.02361300	3.42154500
O	-3.26645700	0.00126600	-0.94782900
O	-1.22241500	-0.01104200	1.01381100
O	2.17854200	0.01103900	-1.07731400
O	2.20799400	-2.81302700	-1.07381000
O	2.19497000	2.83514500	-1.03591400
O	-0.33851000	1.42886700	-1.14650500
O	-0.33276200	-1.41681600	-1.16556400
O	1.22528600	1.43953800	1.19586300
O	1.23119300	-1.45279400	1.17611600
C	-4.22242700	-0.00383100	0.12929300
H	-3.70392600	-0.01316000	1.07432000
C	-5.51575000	0.00240500	-0.09562100
H	-5.95874300	0.01212900	-1.08489100
H	-6.18920400	-0.00236200	0.75037100
H	-3.72497700	0.00646300	-1.80457300
V	0.37550800	-0.01288900	1.86603400

8_{V4}:

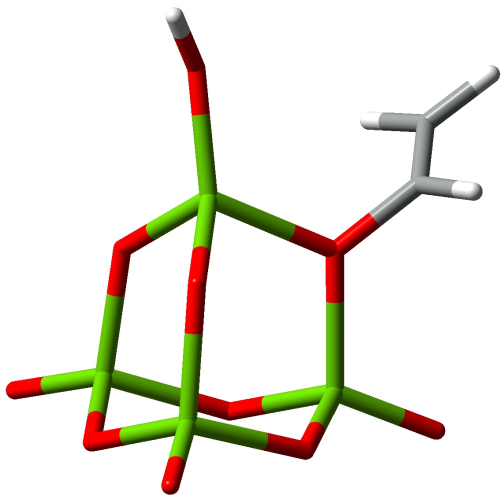

charge = 1, multiplicity = 2

V	0.97703200	-1.53927400	-0.27219000
V	-1.66411600	-0.10970900	-1.35561600
V	-1.18905700	-0.17723700	1.68915800
O	1.11479500	3.16779500	-0.14151100
O	2.10090900	-2.85086900	-0.16390600
O	1.73946100	0.31228500	-0.30166500
O	-2.33537100	-0.28681300	0.26806100
O	-2.73371500	-0.20071800	-2.48962000
O	-1.81443900	-0.40982000	3.10190200
O	0.07932900	-1.33990300	1.29369800
O	-0.27183800	-1.36397600	-1.44201000
O	-0.32271600	1.42732800	1.46564200
O	-0.72502400	1.42619400	-1.34003500
C	3.12584800	0.48304400	-0.58839600
H	3.30863000	0.69704500	-1.63298700
C	4.03393500	0.38715400	0.36278500
H	3.78362200	0.18252300	1.39609500
H	5.07676200	0.52352700	0.10910500
H	2.26050800	-3.72948500	-0.54119600
V	0.48312600	1.74157600	-0.06056000

9_{V4}:

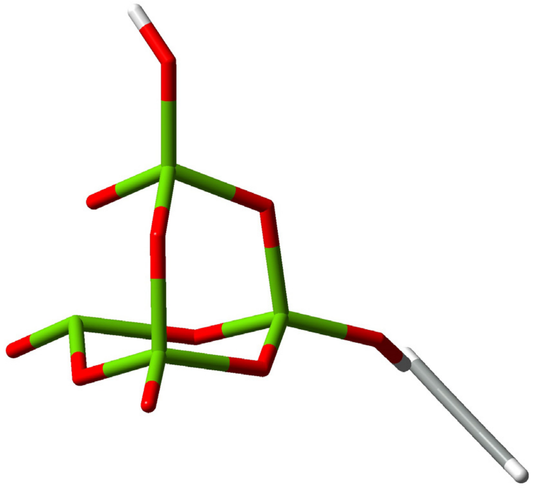

charge = 1, multiplicity = 2

V	1.39512700	1.64342200	0.22013800
V	1.28617600	-1.04370400	-1.42260400
V	0.65786700	-1.14743000	1.66825900
O	-2.98643800	0.60508500	-0.78893800
O	2.20560900	3.16215800	0.34203000
O	-0.32454500	1.75793300	-0.21264400
O	1.33786300	-1.89851200	0.09079700
O	1.96685700	-1.82303800	-2.59347800
O	0.50282000	-1.95110800	2.99830200
O	1.40163600	0.68253700	1.58826700
O	2.01055400	0.62296800	-1.03869500
O	-1.06952200	-0.56395600	0.97193600
O	-0.48444100	-0.56549700	-1.67294000
C	-5.65729700	0.27760500	0.64770300
H	-5.50211000	0.57749300	1.65865900
C	-5.89696800	-0.06530800	-0.47855300
H	-3.91215500	0.43094900	-0.44377600
H	-6.14756400	-0.37078500	-1.46891400
H	2.68236400	3.64860500	1.03243600
V	-1.35126100	0.29603400	-0.43407100

10_{V4}:

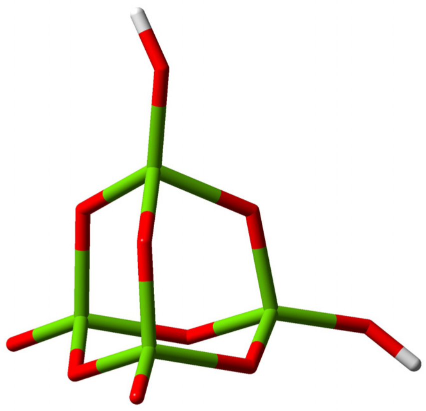

charge = 1, multiplicity = 2

V	-0.03725700	-1.06063300	1.60627400
V	-1.73937200	0.82927200	-0.28060100
V	0.34370400	-1.22249600	-1.45019400
V	1.40648300	1.26488000	0.09870200
O	-3.18244000	1.76816600	-0.25086600
O	2.59846600	2.50637300	0.10701700
O	0.62237400	-2.17271400	-2.65516700
O	-0.13804800	-1.86393200	2.94031600
O	0.30317000	-2.04741300	0.12395200
O	1.57936300	0.10274500	-1.20538900
O	1.30388400	0.25872800	1.47722300
O	-1.50808100	-0.17603700	1.22251800
O	-1.23832200	-0.28448200	-1.53033000
O	-0.18810700	1.86078400	-0.13852200
H	3.37151200	2.76639100	0.63271300
H	-3.98142400	1.96231400	-0.76488500

12_{V4}:

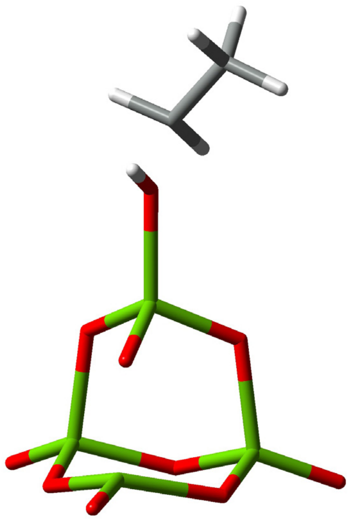

charge = 1, multiplicity = 2

V	-1.54962000	1.67717000	-0.41026200
V	-0.89397900	-0.45335600	1.83819500
V	1.35697000	0.19101000	-0.26116000
O	-1.18860100	-0.84123700	3.32701300
O	-1.77456100	-2.52591800	-2.06197800
O	3.26658200	0.50953800	-0.55266500
O	-2.01078000	3.11894600	-0.81119900
O	0.43072700	1.52479500	-0.58217300
O	0.67898900	-1.04730400	-1.20027900
O	-1.91436900	0.16126800	-1.47977500
O	-1.60229800	1.05697700	1.37298000
O	0.95369200	-0.25439000	1.32444500
O	-1.41222600	-1.67354400	0.62363400
H	3.58560200	1.34800400	-0.92469200
C	4.41473000	-0.45784800	-0.33773900
H	4.66755200	-0.82677900	-1.32975800
H	3.97369300	-1.25668100	0.25496100
C	5.54906700	0.23749900	0.36297300
H	5.95573300	1.05600700	-0.23512300
H	5.25376600	0.61013600	1.34362600
H	6.35366100	-0.48815900	0.50445400
V	-1.21728400	-1.38671400	-1.14263700

13$_{V4}$:

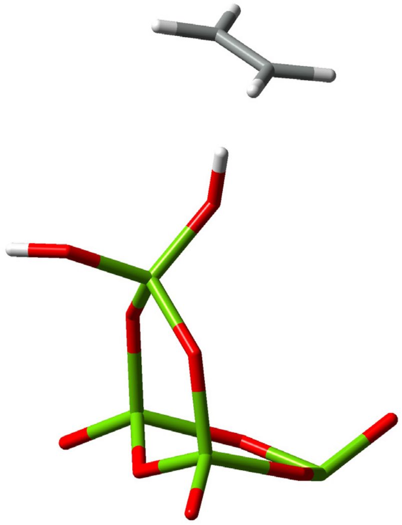

charge = 1, multiplicity = 2

V	1.27628800	-1.37507400	-1.13586700
V	0.91913600	-0.00366200	1.72805800
V	-1.79721400	-0.62019800	0.03968900
V	2.55945700	1.66902100	-0.55245900
O	1.27161200	-0.25639700	3.23430400
O	2.01532400	2.95905100	-1.25867900
O	-2.78396600	-1.91411300	0.63276200
O	1.86664700	-2.73227400	-1.63931300
O	-0.65318300	-1.15413200	-1.07317500
O	-2.75454400	0.60315700	-0.68618200
O	2.18714400	0.30721200	-1.49455600
O	1.49073300	-1.29338800	0.75974600
O	-0.87875600	0.05887700	1.35637900
O	1.80556200	1.47363300	0.98457600
H	-2.86636300	-2.86243100	0.45722900
C	-5.79571400	1.12877600	-0.98169500
H	-6.21048400	0.16093900	-0.72314600
H	-5.72904500	1.36235400	-2.03832300
C	-5.44106300	2.01009100	-0.04588700
H	-5.55274900	1.79419500	1.01078400
H	-3.70756800	0.89027100	-0.59086000
H	-5.07206900	2.99642000	-0.30376200

14_{V4}:

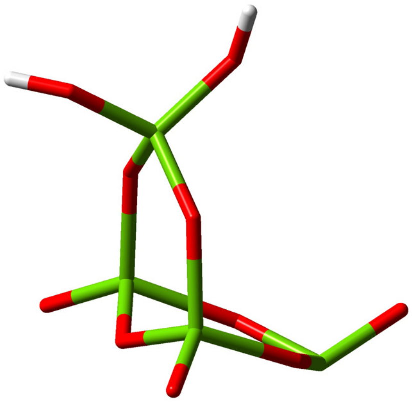

charge = 1, multiplicity = 2

V	0.28229100	1.75472200	-0.59725300
V	0.21958800	-1.41883700	-0.98154100
V	-2.26269800	-0.08507800	0.65925500
V	2.68151400	-0.15645600	0.76508600
O	0.24275800	-2.48595000	-2.12740000
O	2.80993700	-0.32985700	2.31674300
O	-3.80422300	-0.07117000	-0.11876900
O	0.26815800	2.97882100	-1.56708000
O	-1.36680100	1.29454700	0.34462400
O	-2.42190100	-0.24549700	2.38240200
O	1.86476800	1.29440400	0.42726000
O	0.26882100	0.15855400	-1.64105600
O	-1.32165000	-1.40189000	0.03562200
O	1.74272400	-1.44522400	0.10740600
H	-4.33533800	0.56013000	-0.62655400
H	-3.10136500	-0.70410100	2.90096500

15_{V4}:

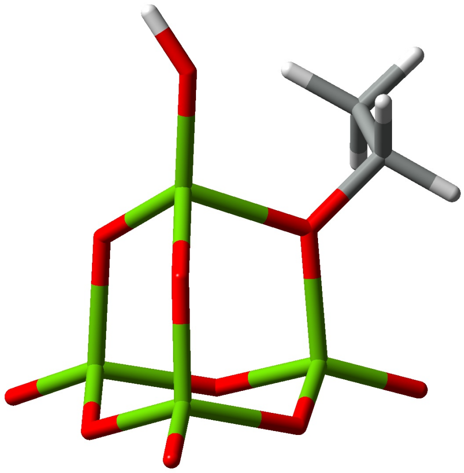

charge = 1, multiplicity = 2

V	1.14634700	-0.37887900	1.70852500
V	1.77800600	0.44086700	-1.19751900
O	2.87646100	0.89506600	-2.21146200
O	-1.21390500	-2.86251500	-1.15777600
O	-1.94499800	2.91846300	0.02581300
O	1.75059800	-0.68838700	3.11514800
O	-0.12639700	0.99729900	1.65040300
O	-1.69284100	-0.10983400	-0.47064800
O	0.20371500	-1.73264400	0.98947700
O	2.35244400	0.07069700	0.49936500
O	0.51157400	1.65054900	-0.95347300
O	0.81298700	-1.03755300	-1.69334000
H	-2.25233700	3.70143800	0.50714300
C	-3.17838700	-0.19637200	-0.69785100
H	-3.30898300	-0.99489100	-1.42559300
H	-3.45461000	0.75169600	-1.15694600
C	-3.90851000	-0.46206300	0.59376300
H	-3.60769900	-1.40979700	1.04132900
H	-3.76602700	0.34050600	1.31999100
H	-4.97744300	-0.52021300	0.37553800
V	-0.87888400	1.56278100	0.20812300
V	-0.49541000	-1.56943200	-0.64891000

16_{V4}:

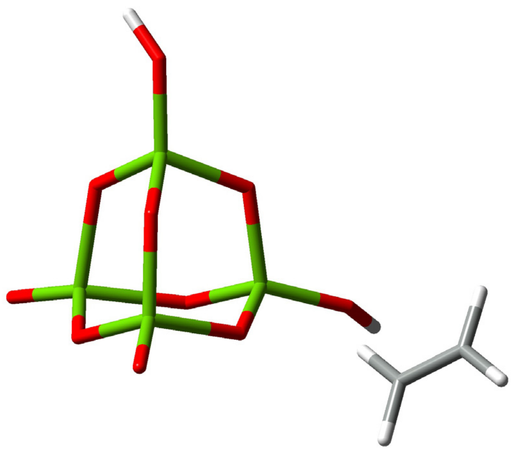

charge = 1, multiplicity = 2

V	-0.75517300	-1.44265800	-1.40164300
V	-1.37405000	-0.71277500	1.60134300
V	-1.39481800	1.60955000	-0.48929400
V	1.32147500	0.29997700	0.33856400
O	-2.01443500	-1.25079600	2.92137700
O	2.96127400	0.61162900	0.65702100
O	-2.15043800	3.09504400	-0.93937700
O	-0.98907100	-2.31428900	-2.67675600
O	-1.45036000	0.37887700	-1.61238100
O	0.34698600	1.73368900	-0.12042700
O	1.02789400	-0.79684700	-0.89767800
O	-1.51330600	-1.82330200	0.26927100
O	-2.02065100	0.90929600	0.96814000
O	0.42848900	-0.31312700	1.70241900
H	-2.60741900	3.43496300	-1.72438000
C	5.95498300	0.56002700	0.19388600
H	6.22621300	0.56013400	1.24354000
H	6.04649500	1.50411900	-0.33127800
C	5.57743600	-0.55673900	-0.43231400
H	5.53742400	-1.51044000	0.08219900
H	3.89371400	0.37217100	0.35539500
H	5.35703500	-0.56620700	-1.49408400

TS2-3$_{V4}$:

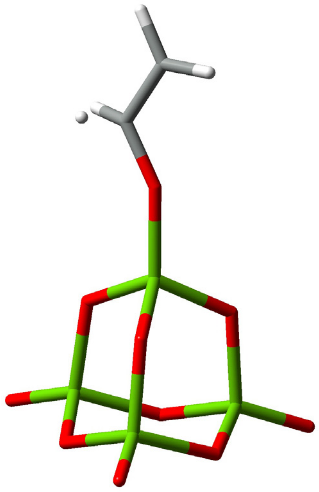

charge = 1, multiplicity = 2

V	-1.17021700	-0.60665400	1.66313700
V	1.43661300	-0.14459300	-0.01515900
V	-1.01682200	1.80217400	-0.26488000
V	-1.22505900	-1.06800900	-1.38834800
O	-1.68376900	-1.10616600	3.05417700
O	3.23511900	-0.20164700	-0.00033900
O	0.65103000	-0.69293200	1.46178100
O	-1.62555400	0.66984100	-1.53292200
O	-1.39672800	3.30526100	-0.47994500
O	-1.79282900	-1.95503900	-2.54520400
O	0.62635100	-1.09573700	-1.22731300
O	0.77983100	1.46759700	-0.24693800
O	-1.75141200	-1.56394600	0.24906600
O	-1.58695800	1.10078600	1.28813200
C	5.62648900	-0.38013500	-0.08477400
H	6.58612100	0.12139300	-0.15099500
H	5.59928800	-1.45690700	0.04969500
C	4.44218300	0.41288400	0.00650800
H	4.72025500	0.74216300	1.07053800
H	4.47782500	1.36561200	-0.54282600

TS2-5$_{V4}$:

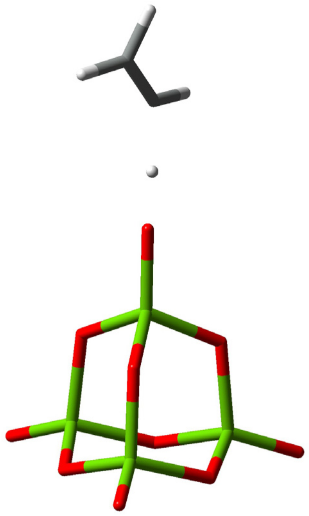

charge = 1, multiplicity = 2

V	1.01828500	-0.21032100	1.82579700
V	-1.34740000	0.15351900	-0.17685100
V	1.17803300	-1.49639100	-0.97762400
V	1.38206500	1.57156000	-0.67351000
O	-0.78641600	-0.05831200	1.46280200
O	1.50699100	-1.59089000	0.78636200
O	-0.64236700	-1.20182400	-1.02428900
O	1.70409400	1.26321700	1.06935800
O	1.85144200	0.06872800	-1.54104400
O	-0.45587200	1.54363800	-0.76149800
O	-3.00177400	0.28138400	-0.32136400
O	1.67151400	-2.73627600	-1.78830000
O	1.38137400	-0.38383800	3.33460700
O	2.04931000	2.86826800	-1.23081800
C	-5.41854500	-0.53564200	0.02497600
H	-4.09550800	-0.08291700	-0.21129800
H	-5.44056300	-1.62061000	0.00731300
C	-6.33599400	0.40460000	0.03610300
H	-7.31824700	0.22477000	-0.40967200
H	-6.15742800	1.40981300	0.41100100

TS7-8$_{V4}$:

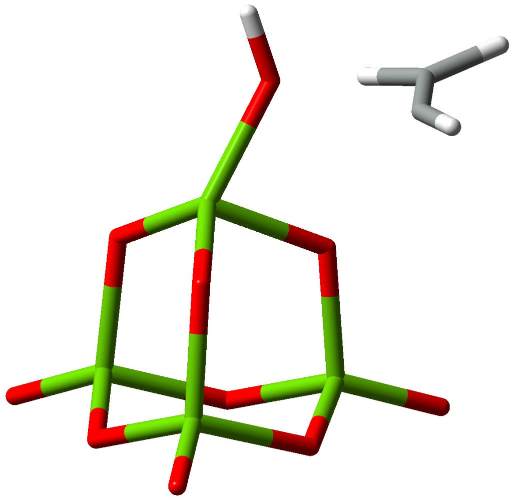

charge = 1, multiplicity = 2

V	-0.86470900	-0.34243300	-1.39134000
V	1.68570600	-1.40605200	-0.01144400
V	1.26940000	1.65655600	-0.39518900
O	-1.13944800	0.18271400	3.06924700
O	-2.59998700	-0.65818900	-1.87709600
O	-1.56938200	-0.21894100	0.31700300
O	2.35670000	0.23013000	-0.36400700
O	2.75202400	-2.54836000	-0.00358600
O	1.99741200	3.00546800	-0.70055400
O	-0.09616300	1.22141000	-1.55191700
O	0.30728100	-1.63933300	-1.19439200
O	0.41550800	1.62480000	1.18249700
O	0.80320700	-1.21212700	1.54089300
C	-3.70775200	-0.53191300	0.05017400
H	-3.58560400	-1.58827100	0.17357200
C	-4.36545200	0.55560700	0.17081300
H	-4.06008700	1.54183100	-0.15384700
H	-5.33873800	0.44800700	0.66003800
H	-3.02873300	-0.77381000	-2.73713800
V	-0.41060700	0.10625500	1.68435300

TS8-9$_{V4}$:

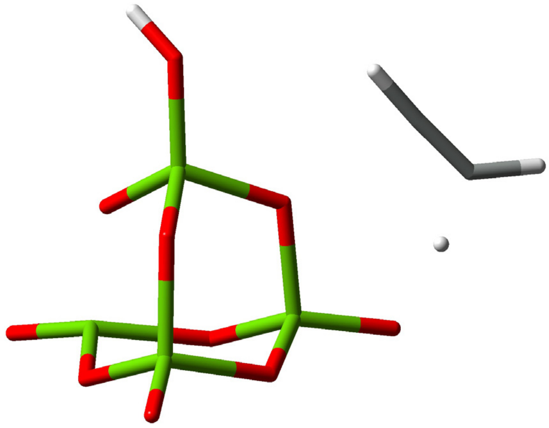

charge = 1, multiplicity = 2

V	0.97684800	-1.31832300	-0.15498600
V	0.40492600	1.72316600	0.06074000
V	-1.39767500	-0.32559200	1.61401800
V	-1.65543700	-0.24465000	-1.54161300
O	2.28025100	-2.28600200	-0.25865000
O	1.14570000	3.29757400	0.08382200
O	1.62865100	0.38961200	-0.04282400
O	-2.38682500	-0.38821800	0.19494800
O	-2.19957800	-0.59227900	2.93166400
O	-2.54766800	-0.08082700	-2.81623900
O	-0.63011700	1.41013400	-1.19769700
O	-0.47212800	1.32130300	1.47608200
O	-0.08701200	-1.34956900	-1.49724800
O	-0.00701500	-1.46566900	1.30891400
C	4.05773900	0.48744500	0.01967200
H	3.79971300	1.52113200	0.11478100
C	4.42767600	-0.69500900	-0.08392100
H	3.53142900	-1.47999900	-0.16887900
H	5.41055900	-1.15665200	-0.10872300
H	0.99254600	4.11656700	-0.41152400

TS12-13$_{V4}$:

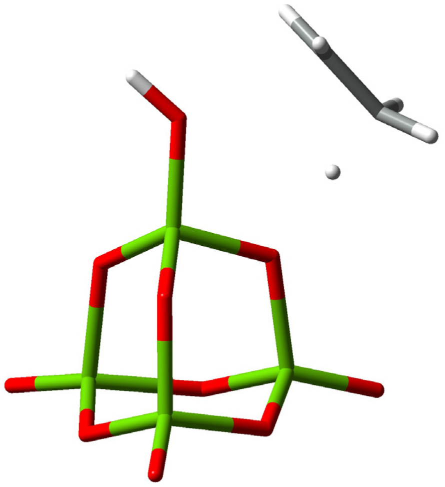

charge = 1, multiplicity = 2

V	1.69676400	-0.51857800	-1.48312700
V	1.46593500	-0.13458200	1.64275800
V	-1.06929000	-1.13778800	0.09663500
V	-0.06825800	1.81703900	-0.27940200
O	2.26659900	-0.21844900	2.98760500
O	-0.53175500	3.30404800	-0.49191600
O	-2.62660400	-2.02443200	0.11588700
O	2.42919800	-1.15827700	-2.71145500
O	0.00935500	-1.46976400	-1.12570300
O	-1.50589900	0.55439500	-0.11528600
O	0.89951200	1.19803400	-1.57367200
O	2.38410800	-0.65590900	0.26711600
O	-0.14706100	-1.14603700	1.53286900
O	0.82513100	1.50756500	1.24276700
H	-2.80477400	-2.94977500	-0.10825300
C	-4.68720200	-0.51041500	0.04781900
H	-4.80003600	-0.97677500	1.02021200
H	-4.89437800	-1.12999700	-0.81759700
C	-4.31519600	0.82560800	-0.08539000
H	-4.44046700	1.45791700	0.79305000
H	-3.13301800	0.68042700	-0.10876400
H	-4.51208400	1.29758900	-1.04681200

TS12-15$_{V4}$:

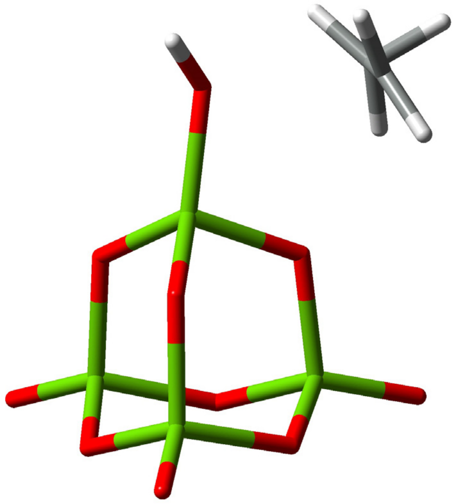

charge = 1, multiplicity = 2

V	1.16359600	1.17985600	-1.32316300
V	1.88651700	-1.09773700	0.63936700
V	-0.90744800	-1.11674200	-0.78065200
O	3.03476400	-2.02509000	1.16188000
O	-0.88866100	1.83122900	2.59495100
O	-2.46633200	-1.79162900	-1.36616500
O	1.68990400	2.14388800	-2.44056000
O	-0.14254800	0.09832900	-1.87710800
O	-1.43221200	-0.08311800	0.62442400
O	0.44807500	2.01485300	0.12086800
O	2.40398600	0.04805400	-0.61358100
O	0.43455700	-1.99492400	-0.11771200
O	1.09913600	-0.11711100	1.91468600
H	-2.63124000	-2.61535600	-1.84629300
C	-4.16278900	-0.52086900	0.42784100
H	-3.63449000	-0.56411800	1.37488800
H	-4.48640700	-1.45716900	-0.01285800
C	-4.56434000	0.71793500	-0.12396400
H	-4.01735300	1.59537600	0.21465300
H	-4.84041600	0.71061200	-1.17720700
H	-5.55098000	0.74668900	0.43620100
V	-0.22621700	1.00878400	1.42853000

TS15-16$_{V4}$:

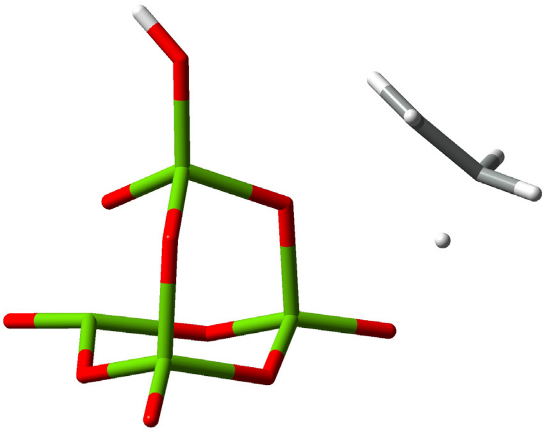

charge = 1, multiplicity = 2

V	-1.69943100	-0.25170500	-1.54211800
V	-1.44554300	-0.32919900	1.61306000
V	0.35576400	1.72353500	0.06017200
V	0.92843900	-1.32013200	-0.15518300
O	-2.24648100	-0.59396800	2.93266600
O	2.21492100	-2.28975300	-0.25488200
O	1.06981800	3.30967100	0.08118100
O	-2.59070600	-0.08842000	-2.81855300
O	-0.67815300	1.40460900	-1.19809400
O	1.58786900	0.39510800	-0.04318200
O	-0.13523300	-1.35218900	-1.50418400
O	-2.43451400	-0.39262200	0.19355500
O	-0.51944200	1.31921500	1.47572900
O	-0.05549800	-1.46543400	1.31570600
H	0.89478600	4.12985100	-0.40432000
C	3.97393800	0.57196000	0.03592900
H	3.76900100	1.01230500	1.00420100
H	3.80999000	1.20260800	-0.82915500
C	4.52822900	-0.70760100	-0.08368800
H	5.00709100	-1.10311200	0.81198400
H	3.56937400	-1.39192300	-0.15783300
H	5.03382200	-0.92310300	-1.02427100
